# Loneliness in pregnant and postpartum people and parents of children aged 5 years or younger: a scoping review

**DOI:** 10.1186/s13643-022-02065-5

**Published:** 2022-09-07

**Authors:** Jacqueline Kent-Marvick, Sara Simonsen, Ryoko Pentecost, Eliza Taylor, Mary M. McFarland

**Affiliations:** 1 University of Utah College of Nursing, University of Utah, 10 2000 E, Salt Lake City, UT 84112 USA; 2 Eccles Health Sciences Library, Salt Lake City, UT USA

**Keywords:** Loneliness, Maternal loneliness, Parental loneliness, Loneliness in parenthood, Loneliness in pregnancy, Perinatal loneliness, Scoping review

## Abstract

**Background:**

Despite evidence that loneliness increases during times of transition, and that the incidence of loneliness is highest in young adults, loneliness during pregnancy and new parenthood has not been developed as a program of research. Because loneliness research has primarily focused on older adults and other high-risk populations, the concept of loneliness and its effects on this population are not well understood, leaving a gap in our understanding of the psychosocial needs and health risks of loneliness on pregnant people and new parents. A scoping review has been completed in order to map and synthesize the literature on loneliness experienced during pregnancy and the first 5 years of parenthood prior to the COVID-19 pandemic.

**Methods:**

To address the aim of this review, a wide net was cast in order to detect experiences of perinatal or parental loneliness and/or instances where loneliness was measured in this population. Among the inclusion criteria were loneliness in people who were pregnant, who were parents in the postpartum period, or who had children aged 5 years or younger. A search for literature was conducted in December 2020 using nine databases: MEDLINE (Ovid), EMBASE (Elsevier), SCOPUS (Elsevier), Cochrane Library including CENTRAL (Wiley), CINAHL (Ebscohost), PsycINFO (Ebscohost), Dissertations & Theses Global (ProQuest) and Sociological Abstracts (ProQuest), and the Web of Science Core Collection (Clarivate).

**Results:**

Perinatal and parental loneliness studies are limited and have rarely been targeted and developed through a program of research. Loneliness inquiry in this population was frequently studied in relation to other concepts of interest (e.g., postpartum depression). Alternatively, the importance of loneliness emerged from study participants as relevant to the research topic during qualitative inquiry. Across studies, the prevalence of loneliness ranged from 32 to 100%. Loneliness was commonly experienced alongside parenting difficulties, with parents feeling as though they were alone in their struggles.

**Conclusions:**

As loneliness has been called a sensitive indicator of mental wellbeing, we believe screening will help healthcare professionals identify common difficulties and early signs of depression experienced during pregnancy and parenthood.

**Systematic review registration:**

The protocol is available on Open Science Framework at DOI 10.17605/OSF.IO/BFVPZ.

**Supplementary Information:**

The online version contains supplementary material available at 10.1186/s13643-022-02065-5.

## Introduction

A substantial number of loneliness studies focusing on older adults and other high-risk groups have associated loneliness with a variety of negative physical and mental morbidity and mortality risks [1–3] including increased systolic blood pressure; depression; impaired sleep [4]; and higher rates of mortality [1]. Little available research, however, addresses loneliness in pregnant and early parenthood populations. Within that small body of work, few studies have made loneliness the clear focal point of attention.

Loneliness is defined as a negative emotional experience related to an appraisal of deficiency within a person’s social network. For example, there may be a perceived deficiency in either the quantity or quality of one’s social contacts [5]. Loneliness is a *subjective* appraisal in contrast to social isolation, which is an *objective* condition of being physically alone. In other words, a person might live a socially isolated life and rarely or never feel lonely, while another might have a dense social network and still feel lonely for lack of a particular type of connection they sense is missing from their network.

Becoming a parent has been described as a major life event because of the significant life transitions with which it is associated [6]. Transitional loneliness may be particularly salient during pregnancy and new parenthood, as it is defined as the experience of loneliness during a period of crisis or developmental changes [7]. Loneliness in this population is common, with 2018 data indicating that just under one in three new parents always or often felt lonely and that 82% experienced loneliness at least some of the time [8]. This can be seen in relation to a study comparing loneliness both in new mothers (32% prevalence) and in a representative sample of the UK public (18%) [9]. And, it can be compared to a meta-analysis, using data from more than 100 countries, of prevalence estimates among young adults (aged 18 to 29) that reveals an overall pooled prevalence of 5.3% and, among middle-aged adults (aged 30 to 59), a prevalence of 6.9% [10]. As loneliness appears to impact pregnant people and new parents at greater rates and with more severity, a scoping review mapping the literature in pregnant people and parents of children aged 5 years or younger is warranted. Just one such related scoping review exists, and is complementary to this review, as it maps the literature on loneliness experienced by parents of children under the age of 16, but does not map the literature that has focused on pregnant people [11].

### Aims and review questions

The aim of our review was to map the literature on loneliness in the perinatal population. As our primary goal was to describe the evidence on the subject of loneliness among pregnant persons and new parents of children under the age of 5, the decision was made to focus on studies published before the COVID-19 pandemic. We excluded studies published in 2020 because these studies were likely to reflect loneliness from experiences of social isolation related to the global pandemic (e.g., loneliness related to lockdown, social-distancing measures, remote work). The entanglement of these factors combined with experiences of loneliness associated with the perinatal period would likely have distorted results due to our inability to distinguish between the various root causes. Records were searched for data that addressed our secondary questions: (a) Within study samples, what aspects of loneliness have been targeted in research on pregnant/parenting individuals? (b) What methodologies have been used and how has loneliness been measured and defined in this population? (c) What is already known about loneliness in this population?

## Methods

We conducted our scoping review under guidance from The Joanna Briggs Institute (JBI) [12–15]. We adhered to the PRISMA reporting guidelines for scoping reviews to ensure transparency and reproducibility [15]. EndNote (Clarivate Analytics) was used to manage citations and remove duplicates, and Covidence (Veritas Health Innovation) was employed to screen and review search results. Our protocol was published in *Systematic Reviews* (DOI: 10.1186/s13643-020-01469-5) [16] and Open Science Framework (DOI 10.17605/OSF.IO/BFVPZ).

### Eligibility criteria

In order to ensure concordance between the inclusion criteria and the research questions, JBI’s Population-Concept-Context (PCC) framework was used [15]. Inclusion criteria targeted a population of pregnant people or parents with children aged 5 years or younger. Parenting a child aged 5 or younger was selected as an age cut-off point because parenting demands are generally greater for younger children, and early parenthood is a period of life marked by rapid transitions in roles and responsibilities. Studies with a focus on loneliness experienced by children were excluded, as were studies with no English-language translation available. All types of publications addressing loneliness within the target population were included in the review process, including gray literature and dissertations.

### Search strategy

**Fig. 1:**
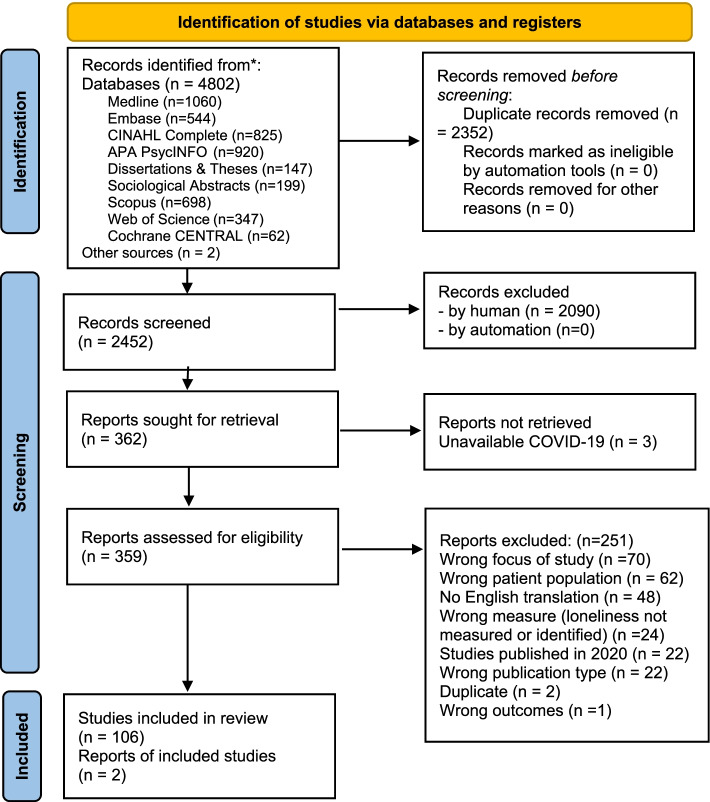
PRISMA 2020 flow diagram. *From:* Page MJ, McKenzie JE, Bossuyt PM, Boutron I, Hoffmann TC, Mulrow CD, et al. The PRISMA 2020 statement: an updated guideline for reporting systematic reviews. BMJ 2021;372:n71. doi: 10.1136/bmj.n71. For more information, visit: http://www.prisma-statement.org/

An information specialist (MMM) developed the searches utilizing a combination of keywords and database subject terms for parental loneliness across nine databases; the search was last updated in December 2020. Please see the supplemental Search Strategies file for full details of the search terms related to each electronic database; search terms included but were not limited to loneliness, lonely, pregnancy, pregnant, parenting, and parents. Although no filters or date limits were applied in database searches, studies published in 2020 were manually excluded to avoid the inclusion of studies conducted during the pandemic. Gray literature was searched via Google search by the first author. Peer review of strategies using the PRESS guidelines was conducted by a library colleague [17]. References of included studies were searched by the first author to identify additional sources. See PRISMA flow diagram of results (Fig. 1).

### Source of evidence screening and selection

The first author (JKM) and one of two additional reviewers (RP, ET) independently screened all titles and abstracts; then, all records were full-text screened by two reviewers. Reviewers reached consensus through discussion when disagreements emerged, obviating the need of an additional reviewer (SS) to resolve conflicts.

### Data extraction

Charting of data was done in the form of a literature matrix within REDCap electronic data-capture software hosted at the University of Utah [18, 19]. Preliminary data-extraction forms were created for a subset of relevant articles as an early check on reliability and thoroughness. Supplemental Tables 1 and 2 include all extracted data. Extracted data included publication details, such as author(s), year, title, journal, country of origin, study aim(s), study design, sample characteristics, study results/main outcomes, types of loneliness identified, definition(s) of loneliness used, factors associated with and protective of loneliness, and prevalence data. In order to maintain timeliness, all data were charted by the first author (JKM), then underwent assessment by either one or another of two additional reviewers (ET and RP), each of whom worked independently from the other; this deviated from our protocol to have two reviewers extract all the data independently from the other. Assessment of extracted data was determined when ET and RP reviewed records for all data-extraction elements found in our matrix (e.g., characteristics of the sample, definition of loneliness, etc.). Then, ET and RP compared their findings with the data extracted by JKM. All data elements were identified by at least two reviewers; only when a discrepancy arose between the first author’s assessment and that of one of the two second reviewers, was the data charted twice. During extraction, the team met when necessary to resolve conflicts and to obtain clarity. Extracted data were synthesized from the literature matrix and are summarized in the Results section that follows. See supplemental Tables 1 and 2 for full details on extracted data.

## Results

This scoping review aimed to cast a wide net using the outlined search strategy for mapping the literature on perinatal loneliness in order to describe the evidence on this subject among our target population. Our secondary questions allowed us to (a) identify which groups have been the focus of perinatal loneliness studies, (b) identify what methodologies have been used and how loneliness has been measured and defined in this population, and (c) summarize our results in order to determine what is already known about loneliness in this population. The results section begins by presenting results related to methodology (i.e., types of loneliness, definitions, measurement, prevalence), then presenting results related to specific groups of parents, and, last, including a section synthesizing the results. Included studies were limited to pre-2020 publication to support the authors’ goal to understand pre- COVID-19 pandemic experiences of parental loneliness. Among pregnant and postpartum people, pre-pandemic prevalence rates of loneliness saw increases in rates ranging between 32 and 42% [9, 20, 21], to rates ranging between 40 and 59% during the pandemic [22, 23], which likely reflects social-distancing measures to curb the spread of COVID-19. Therefore, to map loneliness experienced by pregnant persons and new parents of children under the age of 5, the decision was made to exclude studies reflecting loneliness experienced during the pandemic so as to understand loneliness experienced by this population under more typical conditions. We excluded 22 studies published in 2020 identified in our search results.

### Selection of sources of evidence

A total of 4804 records were identified through searches. After duplicates were removed, a total of 2452 records were imported into Covidence for screening. Title/abstract screening removed 2090 records and moved 362 records to full-text review, which was conducted by three reviewers (RP, ET, JMK) and resulted in 106 studies for inclusion in the review, and two reports from gray literature. See the PRISMA flow diagram (Fig. 1) for details of the selection process, including reasons for exclusion.

**Fig. 2:**
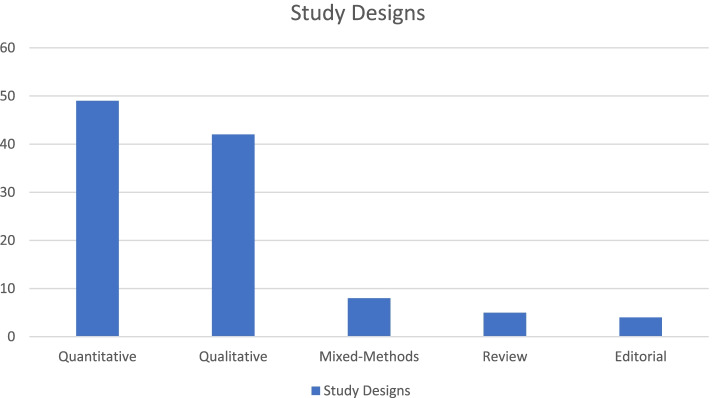
Types of articles included

**Fig. 3:**
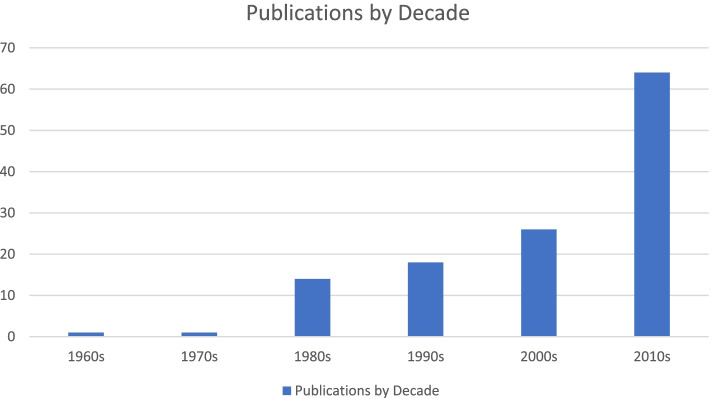
Publications on loneliness through the decades

Forty-nine records were quantitative investigations [6, 20, 21, 24–68], 42 records were qualitative inquiries [69–110], 8 records were mixed or multi methods investigations [9, 111–117], 5 records were review articles [118–122], and 4 were editorials [123–126]. See supplemental Table 1 for full details about each study’s specific aims, study design, and sample. See supplemental Table 2 for full details about the types of loneliness identified, study outcomes, use of loneliness definitions, means of measuring loneliness, factors associated with or protective against loneliness, and prevalence of loneliness. Figures 2 and 3 are provided to illustrate the types of perinatal loneliness studies, as well as the increase in the number of studies across the decades.

### Studies with a primary focus of perinatal or parental loneliness

A minority of the resulting records (*k* = 28, 25.9%) had loneliness as a main focus of interest [6, 9, 20, 24, 31, 33, 36, 39–41, 48, 49, 53, 56–59, 61, 66, 92, 114, 117, 120, 121, 123, 124, 126]. Records with loneliness as a main focus of inquiry originated from Canada [40, 57–59, 117], Finland [6, 20, 39, 61, 66], Japan [24, 48], Malaysia [53], the United Kingdom [9, 92, 120, 123, 126], and the USA [31, 33, 36, 41, 49, 53, 56, 114, 121, 124]. In most quantitative studies, however, loneliness data were collected as co-variates in studies focused primarily on other topics. For example, adolescent pregnancy and postpartum depression were common topics in studies that met inclusion criteria. Loneliness was investigated within these studies to determine its relationship with adolescent pregnancy or postpartum depression. Additionally, quantitative studies focused on unique groups including refugees and child abusers. Qualitative investigations focused primarily on people with postpartum depression and mothers and infants considered medically at high risk. In these qualitative reports, loneliness emerged from participants as a factor that was relevant to their health and wellbeing, and to the qualitative study’s primary concept of interest.

Eighteen of these 28 records were cross-sectional investigations without follow-up [9, 24, 31, 41, 48, 49, 53, 56–59, 92], editorials [123, 124, 126], or review articles [120, 121]. We identified an ongoing longitudinal program of research in Finland focused on mothers’ and fathers’ loneliness over time and in relation to depression, socio-emotional outcomes in children, respiratory infections in children, continuity of maternity care, and family-level influences on social competence in children [6, 39, 61, 66]. Additionally, Rokach [57–59] published three articles about loneliness during pregnancy and motherhood, investigating the antecedents of loneliness and coping techniques found in this population.

### Types of loneliness identified

Lee et al. (2019) qualitatively investigated experiences of loneliness in first-time, non-depressed mothers [92] where both *situational* and *transient* types of loneliness were identified. It should be noted that Lee et al. identified a discrepancy in Young’s (1982) conceptualization of *transient* loneliness [5]. The authors argued that Young’s notion of transient, or “everyday” loneliness, as a normal phenomenon that occurs periodically but resolves, *inadequately* captured the acute nature of the situational and transient loneliness experienced by new mothers. The authors state that the intensity of loneliness experienced by their participants was deeply felt and was threatening to the identities of the mothers. The authors found that loneliness was tied to difficulties experienced during new motherhood, such as struggles related to feeding their babies and feeling fearful and judged by others for not measuring up to the cultural narrative of the ideal mother.

The framework of social and emotional loneliness described by Weiss (1973) was used by several authors for investigating loneliness [6, 39, 66], with evidence that both the social and emotional dimensions of loneliness exist in mothers and fathers [39]. Weiss described two ways of viewing loneliness—the loneliness of social isolation (i.e., social loneliness) and the loneliness of emotional isolation (i.e., emotional loneliness) [127]. Weiss described social loneliness as often coinciding with large role shifts and life changes, such as those experienced during pregnancy and early parenthood [127].

### Definitions of loneliness used

Across studies, loneliness was generally described as a perception of quantitative or qualitative deficiencies in a person’s network. Most articles that used a formal definition of loneliness had roots in this definition, which stems from Perlman and Peplau: “the unpleasant experience that occurs when a person’s network of social relations is deficient in some important way, either quantitatively or qualitatively” (1981, pp. 31) [5]. Additionally, authors frequently sub-categorized loneliness following Weiss’s (1973) concept of social and emotional loneliness (described more completely in the previous section titled *Types of loneliness identified*) [127]. Often loneliness was not clearly defined by authors. To review all definitions used by the sources included in this review, please see supplemental Table 2.

### Measurements of loneliness used

The UCLA Loneliness Scale (adopted in various versions) was the most frequently used measure [128–130]. The UCLA Loneliness Scale includes questions encompassing both social and emotional aspects of loneliness as described by Weiss [39, 61]. Seven articles published between 1981 and 1996 used the 20-item Revised UCLA Loneliness Scale [29, 43, 49, 52, 85, 112, 114]. Russell (1996) updated the scale again in 1996, making Version 3 currently the most up-to-date iteration of the 20-item scale [130]. We note that after 1996, some published studies had a lack of clarity about which version of the UCLA Scale was used. For example, beginning in 1998 and continuing through 2016, six articles [35, 36, 40, 41, 48, 53] reported the use of the 20-item Revised UCLA Loneliness Scale, and often cited Russell et al. (1980), despite Version 3 being the most up-to-date version.

In addition to the loneliness scales described above, some studies used questions from other scales. For example, Santos (2018) assessed loneliness using item number 14 from the Center for Epidemiological Studies Depression scale, “I felt lonely.” [60] Another study investigated differences between child abusing and non-abusing parents using the Michigan Screening Profile of Parenting. This study found that parents who abused their children had a tendency toward isolation and loneliness [65].

Additionally, researchers often created their own items to assess loneliness within their studies [20, 21, 45, 55, 56]. An example of this type of loneliness assessment was the following item: “Do you feel lonely?” with the response items “always,” “often,” “sometimes,” “rarely,” “never,” and “cannot say.” The presence of loneliness was dichotomously categorized as a response of “always,” “often,” or “sometimes,” while not lonely was categorized as a response of “rarely,” or “never” [20]. To review all measures used by the sources included in this review, please see supplemental Table 2.

### Prevalence of loneliness

Although prevalence data were limited, with just six studies reporting the proportion of their sample who experienced loneliness [9, 20, 21, 56, 78, 82], these data illustrate how loneliness is common in this population, and that it increases in special cases, especially as stress increases. Note, the prevalence of loneliness found within individual studies is dependent upon studies’ research questions and the means of classifying people as lonely. Four of these six studies quantitatively assessed loneliness with a single item [9, 20, 21, 56], while the remaining two quantified loneliness described during qualitative interviews [78, 82]. Loneliness among pregnant women and mothers in general ranged from 32 to 42% [9, 20, 21]. Additionally, when parents had a child with a mental or physical health risk, this proportion increased to 70% [78]. All participants (100%) in a study investigating parenting experiences of non-binary and male gestational parents reported loneliness [82].

#### Factors associated with loneliness in mothers and fathers collectively

Rokach (2004) found no significant difference between loneliness scores among pregnant women and new mothers (i.e., women who were less than 1-year postpartum), but did find significantly higher loneliness scores among the study’s sample of women who were not pregnant or new mothers (called women from the “general population”). Notably, the general-population group of participants was composed of significantly more women who identified as single or divorced, compared to the pregnant and new-mother groups [59].

The findings of Junttila et al. (2015), which longitudinally investigated mothers’ and fathers’ loneliness during pregnancy, infancy, and early childhood, are more nuanced and reflect findings of both social and emotional loneliness [6]. Junttila’s sample consisted of approximately 2000 mothers and fathers at each timepoint and used latent class analysis to investigate loneliness during the transition to parenthood. The findings revealed that more than 90% of women were classified as having stable loneliness scores, while the remaining sample of women had increases in loneliness scores following childbirth. Among men, 62.8% were classified as having decreasing loneliness scores following the birth of their children, while the remaining 37.2% had slight increases in loneliness scores [6]. These findings are complementary with their 2013 study, which found that fathers were lonelier during pregnancy and that mothers’ loneliness increased after childbirth [39]. While most participants in the Junttila et al. (2015) study seemed to adapt to new parenthood, lonelier parents experienced more problems with their intimate partnerships, social functioning, and mental wellbeing [6].

#### Factors associated with and protective of loneliness in mothers

Across the literature, mothers frequently described motherhood as imbued with loneliness [70, 92, 124], lacking social support [80, 92], and, sometimes, unsatisfactory with respect to their partners’ or families’ contributions to parenting [48, 91, 92, 103]. Often-cited root causes of maternal loneliness were: a lack of recognition for the difficulties of being a mother [92, 103]; a lack of empathy from relations [92, 103]; childcare burden [24, 48, 73]; deficient social networks [24, 48, 92]; longing for friendships based on shared experience [70, 92]; and discrepancies between expectations and the realities of motherhood [92, 124].

Establishing friendships with other mothers sharing similar experiences was frequently cited as protective against maternal loneliness [70, 92, 101, 111, 123, 124, 126]. Participants from the Lee et al. (2019) study reported that connecting online or in person with other mothers with shared experiences helped them to normalize their difficult experiences of motherhood and helped to provide mothers with a sense of worth in their maternal role. It was found that these types of friendships may be facilitated by (a) mothers themselves [124], (b) trusted community leaders [9], or (c) members of the healthcare team who could help to facilitate opportunities for connection [123].

Mothers’ self-perception of poor health was associated with loneliness [20, 48], and higher loneliness scores (UCLA Loneliness Scale Version 3) were associated with unscheduled hospital use [33]. Mandai et al. (2018) found that as loneliness scores increased (revised UCLA Loneliness Scale), self-reported health status worsened [48], and Geller (2004) found that lonely pregnant women were twice as likely to make unplanned emergency-room or obstetric visits [33], with younger lonely women the most likely to seek unscheduled hospital use.

While there is conflicting data about whether younger or older pregnant women and mothers tend to be lonelier, evidence from this review suggests that both younger and older women experience loneliness. Younger mothers (aged 18 to 24 years old) shared that friends who were not parents had different priorities, making it difficult to stay connected to their existing social network. Additionally, younger mothers reported that visits from their network members dwindled over time as the excitement of the new baby diminished [9]. In comparison, older pregnant women (aged 35 or older) explained that they felt lonely because many of their friends had had children earlier in life and that, as a result, they had no one with whom to share their experience of pregnancy [110].

Breastfeeding and bottle-feeding were identified as sources of mothers’ loneliness. Lee et al. (2019) found breastfeeding limited mothers’ ability to socialize. Mothers felt their partners lacked empathy for the difficulties relating to breastfeeding and that the realities of breastfeeding did not match their expectation that breastfeeding would be easy [92]. Extreme difficulties with breastfeeding left mothers feeling inadequate and often alienated, as they felt they were not living up to expected standards and lacked confidants with whom to talk about these struggles [92, 105]. Communication with other mothers who had struggled to breastfeed was seen as essential to overcoming breastfeeding difficulties and to the successful continuation of breastfeeding [105]. Bottle-feeding was also a source of loneliness when mothers felt judged for not providing the gold standard of breastmilk to their children [92].

#### Factors associated with and protective of loneliness in mothers and fathers experiencing postpartum depression

Studies identified in this review illustrate the significant presence of maternal loneliness in experiences of postpartum depression [74, 95, 98, 100, 118]. Among these results are findings that (a) women with higher loneliness scores during pregnancy and during the postpartum period were more likely to be depressed post-birth [29, 68]; (b) loneliness was found to have a direct negative effect on postpartum depression and infant-mother bonding [44]; and (c) loneliness was positively correlated with postpartum depression [53]. A pattern of loneliness experienced in postpartum depression included the common belief that others did not understand the mothers’ experiences of depression [74, 95, 100, 118], and a sense that attempts to communicate struggles with depression were unsuccessful [74, 95]. These perceptions resulted in mothers feeling alienated from their relations; consequently, mothers isolated themselves from their support network [74, 95, 100, 118].

Women frequently reported that postpartum depression support groups, especially those that were tailored to mothers’ cultural or personal preferences, were helpful at reducing loneliness because these women were able to share mutual concerns while hearing about other women’s experiences of postpartum depression [95, 98, 100, 118]. Group support experiences were described as the first step in recovery, as they provided a place where women finally felt understood [100]. One peer-support intervention using phone and/or online contact was successful in buffering maternal loneliness, reducing postpartum depression and anxiety, and increasing perceived social support [62].

While most studies focused on maternal depression, a few provided data about depression in fathers, as well [6, 47, 66]. Data from the Lutz and Hock (2002) study found that the effect of loneliness on symptoms of depression was greater in fathers than in mothers [47].

#### Factors associated with and protective of loneliness in adolescent mothers

Sixteen studies focused on adolescent mothers as young as age 12 [31, 34–36, 41, 43, 46, 49, 54, 63, 67, 85, 87, 90, 108, 114], revealing conflicting data about whether loneliness is more common in pregnant than non-pregnant adolescents [34, 43, 49, 54, 85]. Studies that found a difference in loneliness scores between pregnant and non-pregnant adolescents [34, 43, 49] found loneliness was reported less often in pregnant adolescents [34], or more often [43], or was experienced more severely in pregnant adolescents [49]. Quantitative studies found that loneliness in adolescent mothers was positively correlated with problematic social support and depression and negatively correlated with self-esteem [35, 36]. Studies examining potential explanations for loneliness in this population found that difficulty with developing personal identities [31] and the effects of pregnancy on the daily lives of adolescents [41] were contributors to loneliness. Qualitative findings underscored the importance of social relationships in adolescents’ decisions about engaging in sexual activity and becoming parents, and illustrated the significance of parental relationships in particular [87, 90, 108].

#### Factors associated with and protective of loneliness in parents who are immigrants or refugees

Ten studies focused on immigrants and refugees [26, 37, 45, 97, 98, 104, 115–117]. Loneliness in pregnancy and the postpartum period was common for these parents, who found themselves isolated from their families and culture [26, 97, 98, 104, 107, 115, 117]. This lack of familial and cultural interaction left parents missing connections with others who shared their values and practices [98, 104]. These feelings were amplified when parents encountered difficulties with childrearing, leaving them with unfulfilled longing for absent family members or friends [98, 104].

An increased prevalence of loneliness was found among immigrant parents (39%) who spoke the country’s official language less than proficiently, compared to citizens of the country (17% in Australian-born women) [26]. Similar findings were found in a study of immigrant women living in Japan who had limited ability to speak the country’s official language [37]. The authors hypothesized that healthcare workers overestimated the immigrant parents’ levels of language proficiency, resulting in increased loneliness and parents feeling less supported in their care [26, 37].

Protective of loneliness in immigrant and refugee parents was the presence of family and friends [97, 107], especially female family members for mothers [97]. Faith and spirituality were also identified as important factors for mental wellbeing [97, 107].

#### Factors associated with and protective of loneliness in male and non-binary gestational parents

Two qualitative studies focusing on transgender male and non-binary parents revealed common experiences of social isolation and loneliness [76, 82], which included a sense of alienation [76] and marginalization [82], with participants describing themselves as struggling to engage with the external world [76, 82]. Participants from all three studies reported problems during pregnancy such as estrangement, deep isolation, and body dysphoria, which was described as a sense of disconnection between “how one feels and how one is perceived” (p. 68) [76]. Both during gender transition and during pregnancy, a lack of understanding and empathy within intimate social-support networks was commonly cited [76, 82].

### Synthesis of results

As of December 2019, there were 108 studies broadly focusing on loneliness among pregnant persons and new parents of children under the age of 5. Notably, loneliness in this population has frequently been studied in relation to other concepts of interest such as postpartum depression. This largely explains the reason that 108 records met our “wide-net” inclusion criteria and that, to a great extent, we are not able to synthesize results characterizing the nature of loneliness experienced in pregnancy and early parenthood. In the records meeting inclusion criteria, the UCLA scale was most often used to measure loneliness in this population, and there was some lack of clarity in reporting which version of this scale was used. These studies found that the prevalence of loneliness among this population ranged from 32 to 100%, and that parenting difficulties contribute to experiences of loneliness. This point is illustrated by prevalence findings from a study focusing on parents who had a child with a mental or physical health risk, which found prevalence of 70% [78], .and all participants (100%) in a study investigating parenting experiences of non-binary and male gestational parents reported loneliness [82].

## Discussion

The results of this review reflect pre-pandemic experiences of loneliness among the pregnant and new parent population. The results indicate that our knowledge of perinatal and parental loneliness remains in a relatively amorphous state and requires more focused research. Several aspects of loneliness were overlooked to a great extent by the studies included in this review: rarely considered were the varying types of loneliness experienced by study participants (e.g., transient, social, emotional; for exceptions see [6, 39, 41, 66, 92]); less often were definitions of loneliness used (for exceptions see [6, 9, 20, 24, 33–35, 39, 41, 44, 48, 52, 54, 61, 66, 85, 92, 120, 121]; and rarely was the prevalence of loneliness reported within samples [9, 20, 21, 56, 78, 82]. Additionally, we identified an inconsistency in the reporting of loneliness measurement when using the revised UCLA Loneliness Scale. Investigators wishing to measure loneliness using the UCLA Loneliness Scale should note that Version 3 of the scale is the most up-to-date iteration [130]. Further, as Junttila et al. (2013) point out, the 20-item UCLA Loneliness Scale is rooted in the theoretical position that loneliness is bi-directional with both social and emotional aspects [39]. This point, combined with Lee et al.’s (2019) finding that transient loneliness is significant, underscores the importance of the conceptual and theoretical foundations of empirical studies of loneliness. And, finally, while studies often reported factors associated with loneliness, less often were protective factors explored. These points illustrate important gaps in our ability to understand the nature of loneliness experienced during pregnancy and during the early years of parenthood.

Parenthood in general is associated with loneliness. The transition into parenthood has been viewed as a major life event which impacts daily activities and the composition of social networks, which in turn impact mothers’ and fathers’ feelings of loneliness; study results summarizing the changes in loneliness across the transition to parenthood have been described as “inconsistent” [131]. Buecker et al. (2020) found lower levels of loneliness during the first year of parenthood, compared to a propensity score-matched control group, then found higher levels of sustained loneliness than in those of the control group across the study’s 9-year duration [131]. While results of the present scoping review indicate parents per se are at risk for loneliness, we found that most studies have focused on sub-groups of parents (e.g., those experiencing postpartum depression, immigrants and refugees, adolescents, parents of children with a physical or intellectual disability), and have rarely attempted to characterize loneliness across pregnancy and stages of new parenthood (see Junttila et al. 2013, 2015 for exceptions). Additionally, loneliness was often not a primary focus of the studies included in this review; loneliness was often reported by perinatal participants of qualitative studies, and loneliness was often measured by a single item in quantitative cross-sectional studies focusing on other aspects of parenthood. As a result, little is known about the characteristics or consequences of loneliness during periods of pregnancy and early parenthood. This is reflected in the inconsistent results of articles attempting to assess changes in loneliness across pregnancy and early parenthood, which have found increases, decreases, and stable levels of loneliness. Conflicting data about how and when loneliness might ebb and flow in this population indicates a missed educational opportunity for the healthcare setting. As loneliness has been shown to be a sensitive indicator for mental wellbeing [61, 132], a focus on perinatal loneliness could help new parents to set realistic expectations and anticipate the potential for perinatal loneliness and related implications [92].

Because prevalence rates indicate loneliness within this population may be elevated, the results also suggest that loneliness might be particularly relevant to pregnancy and new parenthood. Information is lacking about prevention or amelioration of loneliness within this population. The results of this review do indicate two recommendations that relate to healthcare providers and parent groups. First, providers are encouraged to be sensitive to parents’ experiences of loneliness and parental difficulties (e.g., social determinants of health, emotional wellbeing, physical difficulties related to childbirth and/or breast/chest-feeding) and to provide individualized care that addresses parents’ concerns [36, 95, 100, 133]. Additionally, providers can help partners of primary parent caregivers to understand their experiences and needs (e.g., difficulties related to breast/chest-feeding) [91, 92]. Second, participants often voiced a desire for group-setting healthcare or interventions (e.g., postpartum depression or breastfeeding support groups, being connected with other parents) so that they may have the opportunity to share their experiences with other people from similar backgrounds and circumstances [92, 100, 105, 111, 120, 123]. Communicating with others with shared experiences was viewed as therapeutic by these participants. These opportunities could take the shape of informal playgroups [70, 126], telephone calls or online correspondence [48, 62, 81, 92, 101], interactions with supportive family members, friends, and trusted community members [9, 55, 84, 89, 97, 104, 117, 126]. An additional protective factor and promising direction of research relates to the density of maternal social networks. In their sample of pregnant women, Yu et al. (2020) found that social network density, or the degree to which mothers’ relations are connected to one another, was protective against maternal loneliness [134]. The authors hypothesized that more densely connected networks might provide more coordinated care for mothers, and might also create a greater sense of community. Last, information is lacking about how perinatal loneliness may impact family relations, leaving a major gap in our understanding of how loneliness could contribute to adverse childhood experiences [6, 39].

### Limitations

**Fig. 4:**
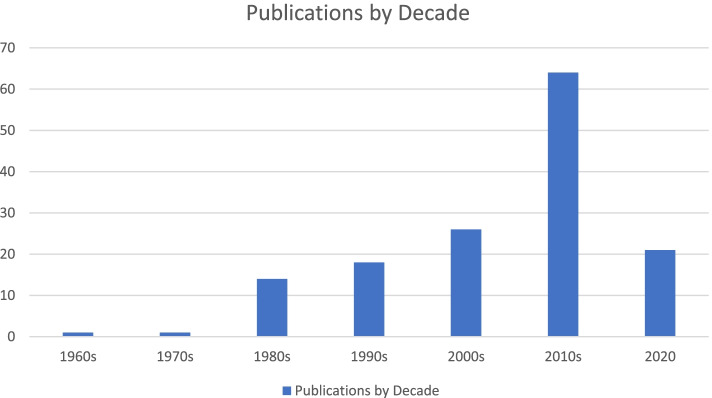
Publications on loneliness through the decades through 2020

The results of this review focused on pre-pandemic experiences of perinatal loneliness. While the authors believe focusing on pre-pandemic perinatal loneliness was essential for mapping current knowledge on the topic in general, we believe there may be important insights to gain from careful examination of loneliness experienced during the pandemic. Additionally, increased publication on this subject, and increased publication in response to maternal mental-health concerns spurred by the onset of the COVID-19 pandemic mean that by the time of publication of this review, there are most likely a number of new relevant articles that have not been captured (see Nowland et al. 2021 for an example [11]). This point is illustrated in Fig. 4, which shows publications by decade through the year 2020 (and which includes 22 new results identified for the year 2020). Last, our English language-only criteria may have limited our results.

## Conclusion

Limited studies are available assessing loneliness during pregnancy and early parenthood independently. Loneliness appears to be experienced at greater rates and with more intensity in sub-groups of pregnant people and new parents who are experiencing additional hardships during the transition to parenthood (e.g., parents who are immigrants or refugees, parents who identify as a gender-variant or male-gestational parent, parents with postpartum depression). Awareness of difficulties experienced during the perinatal period may be useful for addressing mental wellbeing. For example, identifying loneliness in this population may help with early detection of depression, thus screening for loneliness may prove helpful to healthcare professionals. This review of current literature has identified knowledge gaps and needs in this population as well as suggestions for future directions of research.

## Supplementary Information

**Additional file 1: Table 1.** Summary of Documents Included. Information about the records included in this review, as well as additional details including study aims, designs of included studies, and characteristics of the studies’ samples. **Table 2.** Data Extracted on Parental Loneliness contains data related to the research questions of this scoping review, including what type of loneliness was identified (if authors addressed loneliness type), study results, definition of loneliness used (if authors defined loneliness), means for measuring loneliness (if loneliness was measured), factors associated with and protective of loneliness, and prevalence of loneliness within the study sample.

## Data Availability

The protocol for this review was published in *Systematic Reviews* (DOI: 10.1186/s13643-020-01469-5) [16] and Open Science Framework (DOI 10.17605/OSF.IO/BFVPZ).
