# Supplementary material for: Loneliness in pregnant and postpartum people and parents of children aged 5 years or younger: a scoping review
Source: Syst Rev. 2022 Sep 7;11:196. doi: 10.1186/s13643-022-02065-5 (PMC9451126; doi:10.1186/s13643-022-02065-5)
Supplement: Supplementary file 1 — Additional file 1: Table 1. Summary of Documents Included. Information about the records included in this review, as well as additional details including study aims, designs of included studies, and characteristics of the studies’ samples. Table 2. Data Extracted on Parental Loneliness contains data related to the research questions of this scoping review, including what type of loneliness was identified (if authors addressed loneliness type), study results, definition of loneliness used (if authors defined loneliness), means for measuring loneliness (if loneliness was measured), factors associated with and protective of loneliness, and prevalence of loneliness within the study sample. [file 13643_2022_2065_MOESM1_ESM.zip › Excluded studies_254-ScR-Parental-LonelinessR4.docx]

Excluded Studies with reason for exclusion:

1. Did you miss it? Tesco Modern Motherhood survey reveals loneliness of modern mothers. MIDIRS Midwifery Digest. 2008;18(1):147. Exclusion reason: Wrong publication type (commentary).

2. Postpartum Depression and Anxiety. Journal of Midwifery & Women's Health. 2019;64(1):137-8. Exclusion reason: Wrong publication type (commentary).

3. Aching MC, Granato TMM. Role of a support network for refugee mothers. Estudos de Psicologia. 2018;35(2):137-47. Exclusion reason: Wrong measure (e.g. loneliness not measured or identified as theme).

4. Allahdadian M, Irajpour A, Kazemi A, Kheirabadi G. Social support: An approach to maintaining the health of women who have experienced stillbirth. Iran J Nurs Midwifery Res. 2015;20(4):465-70. Exclusion reason: Wrong focus of study.

5. Ansion M, Merali N. Latino immigrant parents’ experiences raising young children in the absence of extended family networks in Canada: Implications for counselling. Couns Psychol Q. 2018;31(4):408-27. Exclusion reason: Wrong measure (e.g. loneliness not measured or identified as theme).

6. Anstett R, Lewis B. The single-parent family. How an understanding physician can help. Postgrad Med. 1986;80(2):137-40, 43. Exclusion reason: Wrong publication type (commentary).

7. Arbona-Vidal C, Duclaux B, Arbona-Margain MF, Ginet D. [Social isolation of the mother and development disorders in young children]. Pediatrie. 1988;43(9):763-7. Exclusion reason: No English translation available.

8. Arnau Sánchez J, Martínez-Ros MT, Castaño-Molina MÁ, Nicolás-Vigueras MD, Martínez-Roche ME. Exploring women’s emotions in perinatal care: A qualitative study. Aquichan. 2016;16(3):370-81. Exclusion reason: No English translation available.

9. Asplin N, Wessel H, Marions L, Georgsson Ohman S. Pregnancy termination due to fetal anomaly: women's reactions, satisfaction and experiences of care. Midwifery. 2014;30(6):620-7. Exclusion reason: Wrong focus of study.

10. Atkins R. Descriptions of Depression in Black Single Mothers...28th Annual Scientific Session, June 2-6, 2017, Baltimore, Maryland. Nurs Res. 2016;65(2):E102-E. Exclusion reason: Wrong publication type (commentary).

11. Atkins R, Gage G, Kelly TA, Joseph PV, Johnson S, Ojo K, et al. Exploring Expressions of Depression in Black Single Mothers. Issues Ment Health Nurs. 2018;39(11):935-45. Exclusion reason: Wrong focus of study.

12. Au A, Chan Y-C, Cheung G, Yuen P, Lee T. Examining the correlation between parenting stress and social support and loneliness in Chinese mothers living in a socially impoverished community in Hong Kong. Journal of Psychology in Chinese Societies. 2008;9(2):169-94. Exclusion reason: Wrong patient population.

13. Auer JM, Bloom PJ. The impact of bed rest and hospitalization on high-risk pregnant women: An occupational therapy perspective. 1998(1388349):105. Exclusion reason: Wrong focus of study.

14. Baba C, Murayama H, Taguchi A, Murashima S. [Loneliness and social relations among mothers with infants]. Nippon Koshu Eisei Zasshi - Japanese Journal of Public Health. 2013;60(12):727-37. Exclusion reason: No English translation available.

15. Babcock GM. Stigma, identity dissonance, and the nonresidential mother. Journal of Divorce & Remarriage. 1997;28(1-2):139-56. Exclusion reason: Wrong focus of study.

16. Baken E, Bazzocchi A, Bertozzi N, Celeste C, Chattat R, D'Augello V, et al. Health and access to health services in immigrant mothers and children: A quantitative and qualitative analysis in the Cesena area Emilia Romagna, Italy. Quaderni ACP. 2007;14(2):56-60. Exclusion reason: No English translation available.

17. Barbosa-Martinez RdC, Benavides-Torres RA, Ramírez-Aranda JM, Benavides-Torres RA, Onofre-Rodríguez DJ, Márquez-Vega MA, et al. Prenatal care in pregnant adolescents. Sex education: Global perspectives, effective programs and socio-cultural challenges. 2018:139-54. Exclusion reason: Wrong measure (e.g. loneliness not measured or identified as theme).

18. Bass L, Jackson MS. A study of drug abusing African-American pregnant women. Journal of Drug Issues. 1997;27(3):659-71. Exclusion reason: Wrong focus of study.

19. Bayley P. Lonely labours and routine enemas: a view of Polish maternity services. British Journal of Midwifery. 2007;15(12):776-7. Exclusion reason: Wrong publication type (commentary).

20. Beck CT, Gable RK, Henshaw C, Elliott S. The Postpartum Depression Screening Scale (PDSS). Screening for perinatal depression. 2005:133-40. Exclusion reason: Wrong focus of study; Mary (SR Core) McFarland (2020-03-24 09:11:06)(Select): Ebook via ProQuest..

21. Bell EC. Isolation of mothers in the community. Lamp. 1981;38:25-6. Exclusion reason: Wrong publication type (commentary).

22. Bellhouse C, Temple-Smith MJ, Bilardi JE. "It's just one of those things people don't seem to talk about..." women's experiences of social support following miscarriage: a qualitative study. BMC Womens Health. 2018;18(1):176. Exclusion reason: Wrong measure (e.g. loneliness not measured or identified as theme).

23. Belot R-A, Vennat D, Moissenet A, Bluon-Vannier A, Herse V, de Montigny F, et al. Accès à la parentalité et isolement familial: La nouvelle solitude des parents = Access to parenthood and family isolation: The new loneliness of parents. Dialogue: Recherches sur le couple et la famille. 2013;199:7-18. Exclusion reason: No English translation available.

24. Belot RA, Vennat D, Moissenet A, Bluon-Vannier A, Herse V, De Montigny F, et al. Access to parenthood and family isolation : the new loneliness of parents. Dialogue. 2013;199(1):7-18. Exclusion reason: No English translation available; Mary (SR Core) McFarland (2020-03-24 09:14:03)(Select): French..

25. Berlincionì V, Broglia D, Bruno D, Gambini F, Lalli G, MarelliMarelli C, et al. Diventare madre nella migrazione: Una ricerca qualitativa sulla maternità nel contesto migratorio = Becoming a mother in a foreign country: A qualitative investigation on maternity in migration context. Interazioni. 2014;1:76-89. Exclusion reason: No English translation available.

26. Berry JO, Jones WH. The parental stress scale: Initial psychometric evidence. Journal of Social and Personal Relationships. 1995;12(3):463-72. Exclusion reason: Wrong patient population.

27. Bjork M, Wiebe T, Hallstrom I. Striving to survive: families' lived experiences when a child is diagnosed with cancer. J Pediatr Oncol Nurs. 2005;22(5):265-75. Exclusion reason: Wrong focus of study.

28. Black K. The deep metaphors of dads' discourse: Exploring conceptual frameworks of fatherhood. 2019;80. Exclusion reason: Wrong focus of study.

29. Bloom TL, Bullock LF, Parsons L. Rural pregnant women's stressors and priorities for stress reduction. Issues Ment Health Nurs. 2012;33(12):813-9. Exclusion reason: Wrong measure (e.g. loneliness not measured or identified as theme).

30. Boemer MR, Mariutti MG. [Women facing abortion: an existential approach]. Rev Esc Enferm USP. 2003;37(2):59-71. Exclusion reason: No English translation available.

31. Boman KK, Viksten J, Kogner P, Samuelsson U. Serious illness in childhood: the different threats of cancer and diabetes from a parent perspective. J Pediatr. 2004;145(3):373-9. Exclusion reason: Wrong patient population.

32. Bouychou M. [A mother facing her child's difficulties]. Soins Pediatrie, Puericulture. 2020;41(316):43-4. Exclusion reason: No English translation available.

33. Brady V, Lalor J. Space for human connection in antenatal education: Uncovering women's hopes using Participatory Action Research. Midwifery. 2017;55:7-14. Exclusion reason: Wrong measure (e.g. loneliness not measured or identified as theme).

34. Buecker S, Denissen JJA, Luhmann M. A propensity-score matched study of changes in loneliness surrounding major life events. J Pers Soc Psychol. 2020. Exclusion reason: Wrong date.

35. Burke TJ, Woszidlo A, Segrin C. Social skills, family conflict, and loneliness in families. Communication Reports. 2012;25(2):75-87. Exclusion reason: Wrong patient population.

36. Burnes DP, Antle BJ, Williams CC, Cook L. Mothers raising children with sickle cell disease at the intersection of race, gender, and illness stigma. Health Soc Work. 2008;33(3):211-20. Exclusion reason: Wrong focus of study.

37. Cagle CS. Women experienced communion, strength, and understanding of their partners' feelings by having their partners present at the birth of their babies [commentary on Bondas-Salonen T. How women experience the presence of their partners at the births of their babies. QUAL HEALTH RES 1998;8(6):784-800]. Evidence Based Nursing. 1999:59-. Exclusion reason: Wrong publication type (commentary).

38. Cameron G. Motivation to join and benefits from participation in parent mutual aid organizations. Child Welfare. 2002;81(1):33-57. Exclusion reason: Wrong focus of study.

39. Canaval GE, González MC, Tovar MC, Valencia C. The 'invisible' in pregnant wome's [sic] experience. Investigacion & Educacion en Enfermeria. 2003;21(2):32-46. Exclusion reason: No English translation available; Mary (SR Core) McFarland (2020-03-24 09:18:45)(Select): Spanish.

40. Cao X, Yang C, Wang D. The Impact on Mental Health of Losing an Only Child and the Influence of Social Support and Resilience. Omega - Journal of Death & Dying. 2018:30222818755284. Exclusion reason: Wrong patient population.

41. Capponi I, Carquillat P, Premberg A, Vendittelli F, Guittier MJ. [Translation and validation in French of the First-Time Father Questionnaire]. Gynecol Obstet Fertil. 2016;44(9):480-6. Exclusion reason: No English translation available.

42. Carraro TE, Knobel R, Frello AT, Gregório VRP, Grüdtner DI, Radünz V, et al. The health team's role in providing care and comfort during labor and childbirth: the puerperae's opinion. Texto & Contexto Enfermagem. 2008;17(3):502-9. Exclusion reason: No English translation available.

43. ClinicalTrials.gov [Internet]. Identifier NCT04485000, Online 1-Day CBT-Based Workshops for PPD Bethesda (MD): National Library of Medicine; 2020 [cited Excluded Last update: February 16, 2021:[Available from: <https://clinicaltrials.gov/show/NCT04485000>. Exclusion reason: Wrong publication type (commentary).

44. Cocozza B. [The silence afterwards. To give birth to a dead infant is a lonely task]. Fag Tidsskr Sykepleien. 1990;78(3):5-8. Exclusion reason: No English translation available.

45. Cook JA. A death in the family: Parental bereavement in the first year. Suicide Life Threat Behav. 1983;13(1):42-61. Exclusion reason: Wrong focus of study.

46. Curran T. Intergenerational Transmissions of Mother-Child Loneliness: A Moderated Mediation Model of Familial Social Support and Conflict Avoidance. Health Communication. 2019;34(10):1166-72. Exclusion reason: Wrong focus of study.

47. Dallay TG. Perinatal grieving of a stillbirth baby. Ann Med Psychol (Paris). 2013;171(3):182-8. Exclusion reason: No English translation available.

48. Darmody E, Bradshaw C, Atkinson SD. Women's experience of obstetric anal sphincter injury following childbirth: An integrated review. Midwifery. 2020;91:102820. Exclusion reason: Wrong date.

49. Dassoff NT. Parental attachment, network orientation and loneliness: Their relationship to social support. 1993;53:6544-. Exclusion reason: Wrong patient population.

50. Davis PB, May JE. Involving fathers in early intervention and family support programs: Issues and strategies. Child Health Care. 1991;20(2):87-92. Exclusion reason: Wrong publication type (commentary).

51. de Carvalho JB, Araujo AC, Costa Ido C, de Brito RS, de Souza NL. [Social representation of fathers regarding their premature child in the Neonatal Intensive Care Unit]. Rev Bras Enferm. 2009;62(5):734-8. Exclusion reason: No English translation available.

52. De'Ath E. Teaching parenting skills. J Fam Ther. 1983;5(4):321-35. Exclusion reason: Wrong focus of study.

53. Delphine V, Danaé P, Denis M. Idéologies hypermodernes, quels enjeux dans la construction de la parentalité? = Hypermodern ideologies and their impact on the construction of parenthood. Bulletin de Psychologie. 2018;556(4):749-57. Exclusion reason: No English translation available.

54. Di Giacomo E, Clerici M. Murderous mothers: Violence, psychopathology and social isolation. Minerva Psichiatr. 2009;50(2):117-21. Exclusion reason: No English translation available.

55. Díaz F EC, Espíndola H JG, Morales C FA. Las funciones yoicas en el embarazo adolescente = The ego functions in the adolescent pregnancy. Revista Chilena de Psicoanálisis. 2008;25(1):57-66. Exclusion reason: No English translation available.

56. Dib S, Rougeaux E, Vazquez-Vazquez A, Wells JCK, Fewtrell M. Maternal mental health and coping during the COVID-19 lockdown in the UK: Data from the COVID-19 New Mum Study. Int J Gynaecol Obstet. 2020;26:26. Exclusion reason: Wrong date.

57. Dorrell SE. The over-30 single mother. 1989(8923280):213. Exclusion reason: Wrong patient population.

58. dos Santos Monteiro JC, Azevedo Gomes F, Spano Nakano AM, O'Brien B. Women's feelings about early contact with their infants on the labour ward. Midwifery. 2011;27(4):484-8. Exclusion reason: Wrong focus of study.

59. Edell-Gustafsson U, Angelhoff C, Johnsson E, Karlsson J, Morelius E. Hindering and buffering factors for parental sleep in neonatal care. A phenomenographic study. J Clin Nurs. 2015;24(5-6):717-27. Exclusion reason: Wrong focus of study.

60. Einav M, Margalit M. Hope, Loneliness and Sense of Coherence among Bereaved Parents. International Journal of Environmental Research & Public Health [Electronic Resource]. 2020;17(8):18. Exclusion reason: Wrong patient population.

61. Ekas NV, Pruitt MM, McKay E. Hope, social relations, and depressive symptoms in mothers of children with autism spectrum disorder. Res Autism Spectr Disord. 2016;29-30:8-18. Exclusion reason: Wrong patient population.

62. Ekwochi U, Asinobi IN, Ifediora C, Ndu IK, Onah SK, Amadi OF, et al. Caring for the sick newborns in nurseries in a developing setting: Evaluation of the psychosocial burden on caregivers. Journal of Clinical Neonatology. 2020;9(1):69-76. Exclusion reason: Wrong date.

63. Engqvist I, Nilsson K. Men's experience of their partners' postpartum psychiatric disorders: narratives from the internet. Mental Health in Family Medicine. 2011;8(3):137-46. Exclusion reason: Wrong focus of study.

64. Ergun S, Ertem G. Difficulties of mothers living with mentally disabled children. JPMA - Journal of the Pakistan Medical Association. 2012;62(8):776-80. Exclusion reason: Wrong patient population.

65. Essex EL, Petras D, Massat CR. Predictors of Loneliness Among Court-Involved and Substance Abusing Mothers. Women & Criminal Justice. 2007;17(2-3):63-74. Exclusion reason: Wrong patient population.

66. Falkenburg JL, Tibboel D, Ganzevoort RR, Gischler SJ, van Dijk M. The Importance of Parental Connectedness and Relationships With Healthcare Professionals in End-of-Life Care in the PICU. Pediatr Crit Care Med. 2018;19(3):e157-e63. Exclusion reason: Wrong publication type (commentary).

67. Farewell CV, Jewell J, Walls J, Leiferman JA. A Mixed-Methods Pilot Study of Perinatal Risk and Resilience During COVID-19. J Prim Care Community Health. 2020;11:2150132720944074. Exclusion reason: Wrong date.

68. Fasullo S. L'angoscia e la solitudine in gravidanza e nel puerperio = Pregnancy anxiety and loneliness. Psichiatria e Psicoterapia. 2007;26(4):336-43. Exclusion reason: No English translation available.

69. Fathi Najafi T, Latifnejad Roudsari R, Ebrahimipour H. The best encouraging persons in labor: A content analysis of Iranian mothers' experiences of labor support. PLoS ONE [Electronic Resource]. 2017;12(7):e0179702. Exclusion reason: Wrong focus of study.

70. Fenner P, Mohamad ZB. "What We Have Shared Is Actually a Big Secret": Group Art Therapy With Divorced Single Mothers in Malaysia. Art Therapy: Journal of the American Art Therapy Association. 2019;36(2):59-67. Exclusion reason: Wrong patient population.

71. Ferguson L. Beating the baby blues. Practising Midwife. 2012;15(2):17-9. Exclusion reason: Wrong publication type (commentary).

72. Fischer J, Anderson VN. Gender Role Attitudes and Characteristics of Stay-at-Home and Employed Fathers. Psychology of Men & Masculinities. 2012;13(1):16-31. Exclusion reason: Wrong focus of study.

73. Fischer OJ. Non-binary reproduction: Stories of conception, pregnancy, and birth. International Journal of Transgender Health. 2020. Exclusion reason: Wrong date.

74. Florian V, Krulik T. Loneliness and social support of mothers of chronically ill children. Soc Sci Med. 1991;32(11):1291-6. Exclusion reason: Wrong patient population.

75. Forinder U, Lindahl Norberg A. "Now we have to cope with the rest of our lives". Existential issues related to parenting a child surviving a brain tumour. Support Care Cancer. 2010;18(5):543-51. Exclusion reason: Wrong patient population.

76. Francisco J. me to we. Good Housekeeping. 2017;264(3):8-. Exclusion reason: Wrong publication type (commentary).

77. Freda MC, Devine KS, Semelsberger C. The lived experience of miscarriage after infertility. MCN, American Journal of Maternal Child Nursing. 2003;28(1):16-23. Exclusion reason: Wrong outcomes.

78. Gaudin JM, Jr., Polansky NA, Kilpatrick AC, Shilton P. Loneliness, depression, stress, and social supports in neglectful families. Am J Orthopsychiatry. 1993;63(4):597-605. Exclusion reason: Wrong patient population.

79. Geadah RR. Some reflexions on the pharmacogenic and therapeutic impact of drug intake on lonely and deprived mothers. Psychol Med (Paris). 1982;14(12):1847-53. Exclusion reason: No English translation available.

80. Gomes VLO, da Fonseca AD, Roballo EC. Social representations of adolescent mothers about parturition. Anna Nery School Journal of Nursing / Escola Anna Nery Revista de Enfermagem. 2011;15(2):300-5. Exclusion reason: No English translation available.

81. Gosztyła T, Prokopiak A. Mediating role of lack of support for the relationship between extraversion and sense of loneliness in parents of children with autism spectrum disorder and parents of children with intellectual disability. Journal of Psychopathology. 2019;25(2):51-7. Exclusion reason: Wrong patient population.

82. Govender D, Naidoo S, Taylor M. "I have to provide for another life emotionally, physically and financially": understanding pregnancy, motherhood and the future aspirations of adolescent mothers in KwaZulu-Natal South, Africa. BMC Pregnancy Childbirth. 2020;20(1):620. Exclusion reason: Wrong date.

83. Greif GL. Lone fathers in the United States: An overview and practice implications. British Journal of Social Work. 1992;22(5):565-74. Exclusion reason: Wrong focus of study.

84. Grootenhuis MA, Last BF. Predictors of parental emotional adjustment to childhood cancer. Psychooncology. 1997;6(2):115-28. Exclusion reason: Wrong patient population.

85. Gulhati A, Minty B. Parental health attitudes, illnesses and supports and the referral of children to medical specialists. Child Care Health Dev. 1998;24(4):295-313. Exclusion reason: Wrong patient population.

86. Hall HK, Millear PM, Summers MJ. Modeling multivariate associations with parental loneliness via perceived closeness and support. Journal of Social & Personal Relationships. 2020;37(8/9):2651-73. Exclusion reason: Wrong patient population.

87. Halsa A. Trapped between madness and motherhood: Mothering alone. Social Work in Mental Health. 2018;16(1):46-61. Exclusion reason: Wrong patient population.

88. Harris P. ALL BY MYSELF. Community Practitioner. 2017;90(12):24-8. Exclusion reason: Wrong publication type (commentary).

89. Hastings-Tolsma M, Nolte AGW, Temane A. Birth stories from South Africa: Voices unheard. Women & Birth: Journal of the Australian College of Midwives. 2018;31(1):e42-e50. Exclusion reason: Wrong measure (e.g. loneliness not measured or identified as theme).

90. Hawthorne DM, Joyner R, Gaucher E, Liehr P. Death of an infant: Accessing the voices of bereaved mothers to create healing. A qualitative study. J Clin Nurs. 2020;28:28. Exclusion reason: Wrong date.

91. Hayashi H, Sayama M. Emotional processes during pregnancy among women successfully conceived via assisted reproductive technology. Journal of Japan Academy of Midwifery. 2009;23(1):83-92. Exclusion reason: No English translation available.

92. Hittner A. Feelings of well-being before and after an abortion. American Mental Health Counselors Association Journal. 1987;9(2):98-104. Exclusion reason: Wrong focus of study.

93. Hoban E, Liamputtong P. Cambodian migrant women's postpartum experiences in Victoria, Australia. Midwifery. 2013;29(7):772-8. Exclusion reason: Wrong measure (e.g. loneliness not measured or identified as theme).

94. Holliday L. Postpartum Loneliness – poems by doctors. Br J Psychiatry. 2020;217(1):389-. Exclusion reason: Wrong focus of study.

95. Holmlund S, Junttila N, Aromaa M, Raiha H, Makinen J, Rautava P. Induced abortion has no psychological effect on early parental self-efficacy and psychological well-being: The Steps to the Healthy Development and Well-being of Children study. Acta Obstet Gynecol Scand. 2020;22:22. Exclusion reason: Wrong date.

96. Honemeyer U, Kurjak A. Pregnancy and loneliness: The therapeutic value of 3D/4D ultrasound. Psychology. 2014;5(7):744-52. Exclusion reason: Wrong focus of study.

97. Horiuchi S, Ishi K, Ota N, Hiruta A, Horiuchi S, Arimori N. Grief care for families experiences perinatal loss: evaluation of bereavement booklet and angel kit. Journal of Japan Academy of Midwifery. 2011;25(1):13-26. Exclusion reason: No English translation available.

98. Horowitz JA, Chang SS, Das S, Hayes B. Women's perceptions of postpartum depressive symptoms from an international perspective. International Nursing Perspectives. 2001;1(1):5-14. Exclusion reason: Unavailable COVID-19.

99. Hu P, Li LY. Exploratory research of coping processes of mothers of children with austism. 2010(10351200). Exclusion reason: No English translation available.

100. Hu X, Li X, Li R, Dou X. [A study on loneliness among people who lost their children in disaster area of 5.12 Earthquake]. Zhong Nan da Xue Xue Bao Yi Xue Ban = Journal of Central South University Medical Sciences. 2014;39(12):1279-84. Exclusion reason: No English translation available.

101. Huang JW, Zhou XY, Lu SJ, Xu Y, Hu JB, Huang ML, et al. Dialectical behavior therapy-based psychological intervention for woman in late pregnancy and early postpartum suffering from COVID-19: a case report. Journal of Zhejiang University SCIENCE B. 2020;21(5):394-9. Exclusion reason: Wrong focus of study.

102. Hubert S, Aujoulat I. Parental Burnout: When Exhausted Mothers Open Up. Front Psychol. 2018;9:1021. Exclusion reason: Wrong patient population.

103. Hurt EA, Hoza B, Pelham WE, Jr. Parenting, family loneliness, and peer functioning in boys with attention-deficit/hyperactivity disorder. J Abnorm Child Psychol. 2007;35(4):543-55. Exclusion reason: Wrong patient population.

104. Husain N, Chaudhry N, Furber C, Fayyaz H, Kiran T, Lunat F, et al. Group psychological intervention for maternal depression: A nested qualitative study from Karachi, Pakistan. World Journal of Psychiatry. 2017;7(2):98-105. Exclusion reason: Wrong measure (e.g. loneliness not measured or identified as theme).

105. Ikonen R, Paavilainen E, Kaunonen M. Trying to Live With Pumping: Expressing Milk for Preterm or Small for Gestational Age Infants. MCN, American Journal of Maternal Child Nursing. 2016;41(2):110-5. Exclusion reason: Wrong measure (e.g. loneliness not measured or identified as theme).

106. Ikonen R, Paavilainen E, Kaunonen M. Trying to live with pumping: expressing milk for preterm or small for gestational age infants. MIDIRS Midwifery Digest. 2017;27(1):101-. Exclusion reason: Wrong focus of study.

107. Isaacs NZ, Andipatin MG. A systematic review regarding women's emotional and psychological experiences of high-risk pregnancies. BMC psychology. 2020;8(1):45. Exclusion reason: Wrong date.

108. Isabel Giraldo Montoya D, Margarita González Mazuelo E, Patricia Henao López C. Experiencias de las mujeres durante el trabajo de parto y parto. Avances en Enfermeria. 2015;33(2):271-81. Exclusion reason: No English translation available.

109. Jiang L, Hao LU, Ruijuan HAN. A qualitative study on psychological experience and emotional needs of infertility patients. Nursing of Integrated Traditional Chinese & Western Medicine. 2019;5(9):45-8. Exclusion reason: No English translation available.

110. Johansson M, Benderix Y, Svensson I. Mothers' and fathers' lived experiences of postpartum depression and parental stress after childbirth: a qualitative study. International Journal of Qualitative Studies on Health and Well-being. 2020;15(1):1722564. Exclusion reason: Wrong date.

111. Jones DP. Parental social isolation. Child Abuse Negl. 1996;20(3):239-40. Exclusion reason: Wrong publication type (commentary).

112. Jungeun K. Mixed Methods Socio-cultural Study of the Process of Maternal Stress Response in the Neonatal Intensive Care Unit (NICU) in South Korea. Mixed Methods Socio-cultural Study of the Process of Maternal Stress Response in the Neonatal Intensive Care Unit (NICU) in South Korea. 2017:1-. Exclusion reason: Wrong measure (e.g. loneliness not measured or identified as theme).

113. Junttila N, Vauras M, Laakkonen E. The role of parenting self-efficacy in children's social and academic behavior. European Journal of Psychology of Education. 2007;22(1):41-61. Exclusion reason: Wrong patient population.

114. Kapan M, Yanıkkerem E. Partner violence, depression and loneliness in pregnant women living in rural and urban areas. TAF Preventive Medicine Bulletin. 2016;15(5):431-9. Exclusion reason: No English translation available.

115. Kecir KA, Rothenburger S, Morel O, Albuisson E, Ligier F. Experiences of fathers having faced with termination of pregnancy for foetal abnormality. Journal of Gynecology Obstetrics and Human Reproduction. 2020:101818. Exclusion reason: Wrong focus of study.

116. Kegler MC, Bird ST, Kyle-Moon K, Rodine S. Understanding teen pregnancy from the perspective of young adolescents in Oklahoma City. Health Promotion Practice. 2001;2(3):242-54. Exclusion reason: Wrong measure (e.g. loneliness not measured or identified as theme).

117. Keizer R, Dykstra PA, Poortman AR. The transition to parenthood and well-being: the impact of partner status and work hour transitions. J Fam Psychol. 2010;24(4):429-38. Exclusion reason: Wrong focus of study.

118. Kent-Marvick J, Simonsen S, Pentecost R, McFarland MM. Loneliness in pregnant and postpartum people and parents of children aged 5 years or younger: a scoping review protocol. Systematic Reviews. 2020;9(1):213. Exclusion reason: Wrong date.

119. Kjelsvik M, Gjengedal E. First-time pregnant women's experience of the decision-making process related to completing or terminating pregnancy--a phenomenological study. Scand J Caring Sci. 2011;25(1):169-75. Exclusion reason: Wrong focus of study.

120. Klass D. An intimate loneliness: supporting bereaved parents and siblings. Mortality. 2000;5(3):331-2. Exclusion reason: Wrong publication type (commentary).

121. Klemets C, Bondas T. Fathers' experiences of their inconsolable crying babies. Hoitotiede. 2005;17(2):69-78. Exclusion reason: No English translation available.

122. Kugler KE, Hansson RO. Relational Competence and Social Support among Parents at Risk of Child Abuse. Family Relations. 1988;37(3):328-32. Exclusion reason: Wrong focus of study.

123. Kulkarni SJ. The Relational Consequences of Interpersonal Violence (IPV) for Adolescent Mothers. Youth and Society. 2009;41(1):100. Exclusion reason: Wrong focus of study.

124. Kumar S, Rathore P, Shweta, Krishnapriya V, Haokip N, Thankachan A, et al. Why I Can't Breastfeed My New-born Baby? Psychosocial Dilemma of a COVID-Positive Post-LSCS Mother. Indian J Palliat Care. 2020;26(Suppl 1):S150-S2. Exclusion reason: Wrong measure (e.g. loneliness not measured or identified as theme).

125. Lara MA, Letechipía G. Suicide ideation and behavior in pregnant women. Salud Mental. 2009;32(5):381-7. Exclusion reason: No English translation available.

126. Lara MA, Letechipia G. Suicidal ideation and suicidal behavior in pregnancy. Salud Mental. 2009;32(5):381-7. Exclusion reason: No English translation available.

127. Lavollay B, Levy A, Bursztejn C. The development of the mother-child link after an early separation. Experience in a neonatology unit. Neuropsychiatr Enfance Adolesc. 1982;30(4-5):241-5. Exclusion reason: No English translation available.

128. Lawford KM, Giles AR, Bourgeault IL. Canada's evacuation policy for pregnant First Nations women: Resignation, resilience, and resistance. Women & Birth: Journal of the Australian College of Midwives. 2018;31(6):479-88. Exclusion reason: Wrong measure (e.g. loneliness not measured or identified as theme).

129. Leahy-Warren P, McCarthy G. Postnatal depression: prevalence, mothers' perspectives, and treatments. Arch Psychiatr Nurs. 2007;21(2):91-100. Exclusion reason: Wrong publication type (commentary).

130. Levy SM. MOTHER-INFANT MUTUAL ATTACHMENT AS IT RELATES TO INFANT ILLNESS IN THE FIRST YEAR OF LIFE. 1983(8320980):194. Exclusion reason: Unavailable COVID-19.

131. Li H, Chen Y. The lived experiences of married women who request early medical abortion with RU486 (mifepristone). Journal of Nursing. 2003;50(4):50-8. Exclusion reason: No English translation available.

132. Lim HS, Choi G. The impact of parenting stress and social support on psychological well -being of Korean mothers of children with mental retardation. 2002(3059454):159. Exclusion reason: Wrong patient population.

133. Loos C, Julius L. The client's view of hospitalization during pregnancy. JOGNN - Journal of Obstetric, Gynecologic, & Neonatal Nursing. 1989;18(1):52-6. Exclusion reason: Wrong focus of study; Ryoko Pentecost (2020-04-07 06:42:09)(Select): Different article.

134. Lourenco MA, Deslandes SF. [Maternal care and breastfeeding experience of women suffering intimate partner violence]. Rev Saude Publica. 2008;42(4):615-21. Exclusion reason: No English translation available.

135. Luciano Marques S, Samantha Souza da Costa P. Experiences of women on the care received during the parturition process. Physis. 2012;22(1):77-97. Exclusion reason: No English translation available.

136. Luoma I, Korhonen M, Salmelin RK, Helminen M, Tamminen T. Long-term trajectories of maternal depressive symptoms and their antenatal predictors. J Affect Disord. 2015;170:30-8. Exclusion reason: Wrong patient population.

137. Luu AM. Early Postpartum Depression Screening. Early Postpartum Depression Screening. 2017:1-. Exclusion reason: Wrong focus of study.

138. Marchetti D, Fontanesi L, Mazza C, Di Giandomenico S, Roma P, Verrocchio MC. Parenting-Related Exhaustion During the Italian COVID-19 Lockdown. J Pediatr Psychol. 2020;45(10):1114-23. Exclusion reason: Wrong patient population.

139. Marin AH, Gomes AG, Sobreira Lopes RdC, Piccinini CA. A constituição da maternidade em gestantes solteiras = Maternity constitution in single pregnant women. PSICO. 2011;42(2):246-54. Exclusion reason: No English translation available.

140. Marscheider E. Ipa Exploration of the Experiences of Mothers of Infants with Congenital Heart Disease Who Have Undergone Surgery in the First Year of Life : and Clinical Research Portfolio. 2020(28126765). Exclusion reason: Wrong date.

141. Martins LA, de Camargo MJG. The meaning of the Occupational Therapy activities in the hospitalization context of high risk pregnancy. Cadernos de Terapia Ocupacional da UFSCar. 2014;22(2):361-71. Exclusion reason: No English translation available.

142. Massie-Taylor K. A MOTHER'S VOICE. Community Practitioner. 2017;90(3):15-. Exclusion reason: Wrong publication type (commentary).

143. Matthey S, Morgan M, Healey L, Barnett B, Kavanagh DJ, Howie P. Postpartum issues for expectant mothers and fathers. JOGNN - Journal of Obstetric, Gynecologic, & Neonatal Nursing. 2002;31(4):428-35. Exclusion reason: Wrong focus of study.

144. Mattila H. The social construction of stay-at-home fathering across everyday spaces. Qualitative Psychology. 2020;7(2):185-205. Exclusion reason: Wrong patient population.

145. Mattila HM. The social construction of stay-at-home fathering across social spaces and places. 2017;78. Exclusion reason: Wrong measure (e.g. loneliness not measured or identified as theme).

146. Mauthner NS. The darkest days of my life: Stories of postpartum depression. Cambridge, MA: Harvard University Press; 2002.

147. Mawn B. Integrating women's perspectives on prenatal human immunodeficiency virus screening: toward a socially just policy. Res Nurs Health. 1998;21(6):499-509. Exclusion reason: Wrong focus of study.

148. Mayers AM, Naples NA, Nilsen RD. Existential issues and coping: a qualitative study of low-income women with HIV. Psychol Health. 2005;20(1):93-113. Exclusion reason: Wrong patient population.

149. McCafferty P, McCutcheon J. Parenting a Child with Autism: Considering the Stresses, Supports and Implications for Social Work Practice. Child Care in Practice. 2020:1-17. Exclusion reason: Wrong patient population.

150. McCallum C, dos Reis AP. [Re-signifying pain, overcoming loneliness: childbirth experiences among working-class adolescents in a public maternity hospital in Salvador, Bahia, Brazil]. Cad Saude Publica. 2006;22(7):1483-91. Exclusion reason: No English translation available.

151. McKinney J, Hickerson L, Guffey D, Hawkins J, Peters Y, Levison J. Evaluation of human immunodeficiency virus–adapted group prenatal care. American journal of obstetrics and gynecology MFM. 2020;2(3). Exclusion reason: Wrong publication type (commentary).

152. McLeod Hiser KR, Cerepani MJ, Cassidy B. Evaluating Depressive Symptomatology, Maternal Loneliness, and Maternal Self-Esteem in Mothers Who Attend a New Moms Peer Support Group. Journal of Pediatric Healthcare. 2017;31(4):427-8. Exclusion reason: Wrong publication type (commentary).

153. McParlin C, Graham RH, Robson SC. Caring for women with nausea and vomiting in pregnancy: new approaches. British Journal of Midwifery. 2008;16(5):280-5. Exclusion reason: Wrong focus of study.

154. McRae MJ. "Condemned to loneliness"--a necessary maternity care decree? MCN, American Journal of Maternal Child Nursing. 1977;2(6):374-7. Exclusion reason: Wrong publication type (commentary).

155. Mead M. Review of Teenagepregnancy and reproductive health. Primary Health Care Research and Development. 2007;8(4):378-. Exclusion reason: Wrong publication type (commentary).

156. Medora NP, Wilson S, Larson JH. Attitudes toward parenting strategies, potential for child abuse, and parental satisfaction of ethnically diverse low-income U.S. mothers. J Soc Psychol. 2001;141(3):335-48. Exclusion reason: Wrong patient population.

157. Meijer A. Elective mutism in children. Isr Ann Psychiatr Relat Discip. 1979;17(2):93-100. Exclusion reason: Wrong patient population.

158. Mijuskovic B. Child abuse and neglect, dependent personalities, and loneliness. Psychology: A Journal of Human Behavior. 1990;27(1):1-10. Exclusion reason: Wrong patient population.

159. Milne SJ, Corbett GA, Hehir MP, Lindow SW, Mohan S, Reagu S, et al. Effects of isolation on mood and relationships in pregnant women during the covid-19 pandemic. Eur J Obstet Gynecol Reprod Biol. 2020;252:610-1. Exclusion reason: Wrong date.

160. Milner JS, Wimberley RC. An inventory for the identification of child abusers. J Clin Psychol. 1979;35(1):95-100. Exclusion reason: Wrong focus of study.

161. Montgomery-Andersen RA, Willen H, Borup I. 'There was no other way things could have been.' Greenlandic women's experiences of referral and transfer during pregnancy. Anthropol Med. 2010;17(3):301-13. Exclusion reason: Wrong focus of study.

162. Morais GS, da Costa SF. [Existential experience of mothers of hospitalized children in intensive pediatric care unit]. Rev Esc Enferm USP. 2009;43(3):639-46. Exclusion reason: Wrong patient population.

163. Moreira Rodrigues AR, Paiva Rodrigues D, Moura da Silveira MA, de Maria Gomes Paiva A, de Melo Fialho AV, Azevedo Queiroz AB. Hospital admission in high-risk pregnancies: the social representations of pregnant women. Revista de Enfermagem Referência. 2020(3):1-7. Exclusion reason: Wrong focus of study.

164. Murray L, McDonald N, Thang VV. Mothers' experiences of settling infants in central Vietnam "through their eyes": A photo-elicitation study. Health Care Women Int. 2019;40(5):495-514. Exclusion reason: Wrong focus of study.

165. Muth C, Exler U, Holzgreve W. Psychologic digestion of genetically indicated termination in the second trimester. Z Geburtshilfe Perinatol. 1989;193(2):96-9. Exclusion reason: No English translation available.

166. Muth C, Exler U, Miny P, Holzgreve W. [Psychological adjustment to abortion for genetic indications in the 2d trimester]. Z Geburtshilfe Perinatol. 1989;193(2):96-9. Exclusion reason: No English translation available.

167. Mwangi MW, Luckett T. Authoritative Parenting Style Among Kenyan Immigrants in Phoenix, Az [Doctoral dissertation]: Grand Canyon University; 2020. Exclusion reason: Wrong patient population.

168. Nahas V, Amasheh N. Culture care meanings and experiences of postpartum depression among Jordanian Australian women: a transcultural study. J Transcult Nurs. 1999;10(1):37-45. Exclusion reason: Wrong measure (e.g. loneliness not measured or identified as theme).

169. Naoko M. Pregnant women's loneliness: Correlated factors and its impacts on maternal role identification and common complaints during pregnancy. Journal of Japan Academy of Midwifery. 2017;31(1):23-33. Exclusion reason: No English translation available.

170. Ndiaye S, Bosowski J, Tuyisenge L, Penn-Kekana L, Thorogood N, Moxon SG, et al. Parents as carers on a neonatal unit: Qualitative study of parental and staff perceptions in a low-income setting. Early Hum Dev. 2020;145:105038. Exclusion reason: Wrong date.

171. Nicholson JH, DeVoe ER. Thrown back: Reintegration experiences of National Guard/Reserve mothers of young children. Child & Family Social Work. 2020;25:188-97. Exclusion reason: Wrong date.

172. Nilsson C, Bondas T, Lundgren I. Previous birth experience in women with intense fear of childbirth. JOGNN - Journal of Obstetric, Gynecologic, & Neonatal Nursing. 2010;39(3):298-309. Exclusion reason: Wrong focus of study.

173. Noriko O, Megumi M, Hanako M, Yasuko M. Birth experience and postnatal depression: women's negative evaluation as a risk factor. Asian Journal of Nursing. 2007;10(4):257-64. Exclusion reason: Unavailable COVID-19.

174. O'Driscoll T, Kelly L, Payne L, St Pierre-Hansen N, Cromarty H, Minty B, et al. Delivering away from home: the perinatal experiences of First Nations women in northwestern Ontario. Canadian Journal of Rural Medicine. 2011;16(4):126-30. Exclusion reason: Wrong measure (e.g. loneliness not measured or identified as theme).

175. Oakley A, Rajan L, Turner H. Evaluating parent support initiatives: lessons from two case studies. Health & Social Care in the Community. 1998;6(5):318-30. Exclusion reason: Wrong focus of study.

176. Oberc MC. Perceptions of isolation by mothers of apnea monitored infants. 1985(MSN):N.PAG p-N.PAG p. Exclusion reason: No English translation available.

177. Ortega DM. How much support is too much? Parenting efficacy and social support. Children and Youth Services Review. 2002;24(11):853-76. Exclusion reason: Wrong patient population.

178. Oulai S, Cisse L, Enoh J, Yao A, Maho S, Andoh J. [The psychological experience of the mothers whose babies were born deformed in the neonatological ward of the academic hospital center of Treichville (Cote-d'Ivoire)]. Arch Pediatr. 2008;15(4):357-61. Exclusion reason: No English translation available.

179. Panula V, Junttila N, Aromaa M, Rautava P, Räihä H. Parental psychosocial well-being as a predictor of the social competence of a child. Journal of Child and Family Studies. 2020. Exclusion reason: Wrong date.

180. Parry YK, Ankers MD, Abbott S, Willis L, Thorpe L, O'Brien T, et al. Antenatal Dads and First Year Families program: a qualitative study of fathers' and program facilitators' experiences of a community-based program in Australia. Prim Health Care Res Dev. 2019;20:e154. Exclusion reason: Wrong focus of study.

181. Pearlman A. Developing content for skills and support interventions for mothers and fathers of children with feeding disorders. 2015(10137230). Exclusion reason: Wrong patient population.

182. Pelentsov LJ, Fielder AL, Laws TA, Esterman AJ. The supportive care needs of parents with a child with a rare disease: results of an online survey. BMC Fam Pract. 2016;17:88. Exclusion reason: Wrong patient population.

183. Pereira de Souza FL, Matozinhos Clark L, Brazão Lelis BD, de Sousa Dusso MI, Moraes Leite A. Feelings and Meanings: HIV in the Impossibility of Breastfeeding. Journal of Nursing UFPE / Revista de Enfermagem UFPE. 2019;13:1055-60. Exclusion reason: Wrong focus of study.

184. Phillips MF. Loneliness in mothers of boys with Duchenne Muscular Dystrophy: case study methodology. 1995(PH.D.):169 p- p. Exclusion reason: Wrong patient population.

185. Phillips MF. Loneliness in mothers of boys with duchenne muscular dystrophy: Case study methodology. 1996;56:6038-. Exclusion reason: Duplicate.

186. Pillai V, Collins A, Morgan R. Family Walk-In Centre--Eaton Socon: evaluation of a project on preventive intervention based in the community. Child Abuse Negl. 1982;6(1):71-9. Exclusion reason: Wrong patient population.

187. Pitel L, Psenkova P, Jones CCG, Zahumensky J. Validation of the Slovak version of the Wijma Delivery Expectancy/Experience Questionnaire (W-DEQ), version A. Bratisl Lek Listy. 2020;121(6):415-21. Exclusion reason: Wrong focus of study.

188. Polansky NA. Determinants of Loneliness among Neglectful and Other Low-Income Mothers. Journal of Social Service Research. 1985;8(3):1-15. Exclusion reason: Wrong patient population.

189. Polansky NA, Ammons PW, Gaudin JM. Loneliness and isolation in child neglect. Soc Casework. 1985;66(1):38-47. Exclusion reason: Wrong patient population.

190. Pontes VV, Chaves SS, Bastos AC, Uriko K, Valsiner J. Having recurrent gestational losses: Persistence in living. Cultural dynamics of women's lives. 2012:489-508. Exclusion reason: Wrong measure (e.g. loneliness not measured or identified as theme).

191. Pozzo ML, Brusati V, Cetin I. Clinical relationship and psychological experience of hospitalization in "high-risk" pregnancy. Eur J Obstet Gynecol Reprod Biol. 2010;149(2):136-42. Exclusion reason: Wrong measure (e.g. loneliness not measured or identified as theme).

192. Praetorius R, Maxwell D, Alam K. Wearing a happy mask: mother's expressions of suicidality with postpartum depression. Social Work in Mental Health. 2020;18(4):429-59. Exclusion reason: Wrong focus of study.

193. Prodgers A. Psychopathology of the physically abusing parent: a comparison with the borderline syndrome. Child Abuse Negl. 1984;8(4):411-24. Exclusion reason: Wrong patient population.

194. Ravelo SE, Flynn J. A chaplain's responsive coaching-based model platform to impart effective coping skills for military personnel who have suffered prenatal or postnatal loss. 2014(3580221):250. Exclusion reason: Wrong focus of study.

195. Razani N, Morshed S, Kohn MA, Wells NM, Thompson D, Alqassari M, et al. Effect of park prescriptions with and without group visits to parks on stress reduction in low-income parents: SHINE randomized trial. PLoS ONE [Electronic Resource]. 2018;13(2):e0192921. Exclusion reason: Wrong focus of study.

196. Ren Q, Ye M. Losing children and mental well-being: evidence from China. Applied Economics Letters. 2017;24(12):868-77. Exclusion reason: Wrong patient population.

197. Rhynes L, Hayslip B, Jr., Caballero D, Ingman S. The beneficial effects of senior center attendance on grandparents raising grandchildren. Journal of Intergenerational Relationships. 2013;11(2):162-75. Exclusion reason: Wrong patient population.

198. Ribaillier C, Poizat A, Di Marco L. Approach to the lived experience of men during a medical abortion at the Hôpital Couple-Enfant in Grenoble: A qualitative analysis. Revue Sage - Femme. 2018;17(5):203-7. Exclusion reason: No English translation available.

199. Roberts-Smith S, Allen DF, Jekel JF. Teenage pregnancy in the Bahamas: trends and characteristics. Yale J Biol Med. 1984;57(3):273-81. Exclusion reason: Wrong measure (e.g. loneliness not measured or identified as theme).

200. Rodrigues L, Lima DD, de Jesus JVF, Neto GL, Turato ER, Campos CJG. Understanding bereavement experiences of mothers facing the loss of newborn infants. Revista Brasileira de Saude Materno Infantil. 2020;20(1):65-72. Exclusion reason: Wrong date.

201. Rodriguez-Almagro J, Hernandez-Martinez A, Rodriguez-Almagro D, Quiros-Garcia JM, Martinez-Galiano JM, Gomez-Salgado J. Women's Perceptions of Living a Traumatic Childbirth Experience and Factors Related to a Birth Experience. International Journal of Environmental Research & Public Health [Electronic Resource]. 2019;16(9):13. Exclusion reason: Wrong focus of study.

202. Rokach A. Loneliness among loved ones: Alienation in the family. Psychology and Education: An Interdisciplinary Journal. 2003;40(3-4):1-18. Exclusion reason: Wrong focus of study.

203. Ross RF. A preliminary investigation of parental loneliness. 1993;53:2748-. Exclusion reason: Wrong patient population.

204. Sato M, Tadaka E, Arimoto A. Factors associated with loneliness among mothers with 4-month-old or 18-month-old infants in an urban area in Japan. Nippon Koshu Eisei Zasshi - Japanese Journal of Public Health. 2014;61(3):121-9. Exclusion reason: No English translation available.

205. Schlesinger B. Jewish Mother-Headed One-Parent Families: Impressions from a Canadian Study. J Psychol Judaism. 1990;14(3):169-88. Exclusion reason: Duplicate.

206. Schlesinger B. Jewish female-headed one-parent families. Journal of Divorce & Remarriage. 1991;17(1-2):201-9. Exclusion reason: Wrong patient population.

207. Schwank S, Lindgren H, Wickberg B, Fu S-C, Yan D, Andersson E. When a New Mother Becomes Mentally Unhealthy, It Is Everyone's Problem: Shanghai Women's Perceptions of Perinatal Mental Health Problems. Women's Reproductive Health. 2020;7(3):190-204. Exclusion reason: Wrong date.

208. Sha'ked A, Rokach A. Addressing loneliness: Coping, prevention and clinical interventions. New York, NY: Routledge/Taylor & Francis Group; 2015.

209. Sha'ked A, Rokach A, Morris R. Marital distress, loneliness and children's adjustment. Psychology and Education: An Interdisciplinary Journal. 2013;50(3-4):11-8. Exclusion reason: Wrong focus of study.

210. Shahar G. Maternal personality and distress as predictors of child neglect. Journal of Research in Personality. 2001;35(4):537-45. Exclusion reason: Wrong patient population.

211. Sharpe L, Conron MK. Making the Most of Bed Rest: Weekly Support Group and Education for Hospitalized Antepartum Patients. JOGNN: Journal of Obstetric, Gynecologic & Neonatal Nursing. 2014;43(Supp 1):S10-S. Exclusion reason: Wrong focus of study.

212. Sheng Q, Zhang X, Cai C, Shi Y. Parents' Experiences of Caring for Their Only Child With Mental Illness in China: A Qualitative Study. J Psychosoc Nurs Ment Health Serv. 2020:1-9. Exclusion reason: Wrong patient population.

213. Shorey S, Chee C, Chong YS, Ng ED, Lau Y, Dennis CL. Evaluation of Technology-Based Peer Support Intervention Program for Preventing Postnatal Depression: Protocol for a Randomized Controlled Trial. JMIR Research Protocols. 2018;7(3):e81. Exclusion reason: Wrong focus of study.

214. Shulman L, Gitterman A, Shulman L. Healing the hurts: Single parents. Mutual aid groups, vulnerable populations, and the life cycle, 2nd ed. 1994:349-63. Exclusion reason: Wrong patient population.

215. Silverstein M, Reid S, DePeau K, Lamberto J, Beardslee W. Functional interpretations of sadness, stress and demoralization among an urban population of low-income mothers. Maternal & Child Health Journal. 2010;14(2):245-53. Exclusion reason: Wrong patient population.

216. Slomian J, Emonts P, Vigneron L, Acconcia A, Reginster JY, Oumourgh M, et al. Meeting the Needs of Mothers During the Postpartum Period: Using Co-Creation Workshops to Find Technological Solutions. JMIR Research Protocols. 2017;6(5):e76. Exclusion reason: Wrong focus of study.

217. Smith CS. Substance abuse, chronic sorrow, and mothering loss: relapse triggers among female victims of child abuse. J Pediatr Nurs. 2009;24(5):401-12. Exclusion reason: Wrong patient population.

218. Smith RL, Stagnitti K, Lewis AJ, Pepin G. The views of parents who experience intergenerational poverty on parenting and play: a qualitative analysis. Child Care Health Dev. 2015;41(6):873-81. Exclusion reason: Wrong focus of study.

219. Soares MJ, Magano S, Marques M, Bos S, Telmeira AT, Maia B, et al. The effects of prenatal maternal stress, loneliness/helplessness, depressive symptoms, sleep difficulties and social support on child temperament in 3 months postpartum. Eur Psychiatry. 2019;56:S318-S. Exclusion reason: Wrong measure (e.g. loneliness not measured or identified as theme).

220. Sommer P. Balancing Bicultural Heritage: Mexican American Teen Mothers' Lived Experience. 2014(Ph.D.):105 p- p. Exclusion reason: Wrong focus of study.

221. Sommer P. Balancing bicultural heritage: Mexican American teen mothers' lived experience. 2016;76. Exclusion reason: Wrong focus of study.

222. Sperlich S, Arnhold-Kerri S, Geyer S. What accounts for depressive symptoms among mothers?: the impact of socioeconomic status, family structure and psychosocial stress. International Journal of Public Health. 2011;56(4):385-96. Exclusion reason: Wrong patient population.

223. Stack S. Marriage, family and loneliness: A cross-national study. Sociological Perspectives. 1998;41(2):415-32. Exclusion reason: Wrong patient population.

224. Stewart M, Spitzer DL, Kushner KE, Shizha E, Letourneau N, Makwarimba E, et al. Supporting refugee parents of young children: “knowing you’re not alone”. International Journal of Migration, Health & Social Care. 2018;14(1):15-29. Exclusion reason: Wrong focus of study.

225. Sword W. Review: mothers with postpartum depression had to readjust expectations, cope with loss, and find ways to meet needs. Evidence Based Mental Health. 2002;5(4):128-. Exclusion reason: Wrong focus of study.

226. Szeverenyi P, Poka R, Hetey M, Torok Z. Contents of childbirth-related fear among couples wishing the partner's presence at delivery. Journal of Psychosomatic Obstetrics & Gynecology. 1998;19(1):38-43. Exclusion reason: Wrong measure (e.g. loneliness not measured or identified as theme).

227. Takegata M, Haruna M, Morikawa M, Yonezawa K, Komada M, Severinsson E. Qualitative exploration of fear of childbirth and preferences for mode of birth among Japanese primiparas. Nurs Health Sci. 2018;20(3):338-45. Exclusion reason: Wrong focus of study.

228. Takenoue K, Satoh T, Matsuyama T. Women's mental states after spontaneous abortion (2) -- focusing on husbands' reactions and women's feeling for pregnancy and sexual intercourse. Journal of Japan Academy of Midwifery. 2001;14(2):5-17. Exclusion reason: No English translation available.

229. Tcheng-Laroche F, Prince RH. Middle income, divorced female heads of families: their lifestyles, health and stress levels. Canadian Journal of Psychiatry - Revue Canadienne de Psychiatrie. 1979;24(1):35-42. Exclusion reason: Wrong patient population.

230. Teffo ME, Rispel LC. 'I am all alone': factors influencing the provision of termination of pregnancy services in two South African provinces. Glob Health Action. 2017;10(1):1347369. Exclusion reason: Wrong patient population.

231. Thelen TH, Alumbaugh RV. Relative body weight as a factor in the decision to abort. Psychol Rep. 1983;52(3):763-75. Exclusion reason: Wrong focus of study.

232. Thiele P. A million little pieces: Parental and physician perspectives on trisomy 18. A. parent's perspective. J Neonatal Perinatal Med. 2012;5(1):1-7. Exclusion reason: Wrong focus of study.

233. Thupayagale-Tshweneagae G. Psychosocial effects experienced by grandmothers as primary caregivers in rural Botswana. J Psychiatr Ment Health Nurs. 2008;15(5):351-6. Exclusion reason: Wrong patient population.

234. Tirgari B, Rayyani M, Cheraghi MA, Mangeli M. Experiences of Iranian Teen Mothers with Parenting Stress: A Qualitative Study. Comprehensive Child & Adolescent Nursing. 2019:1-14. Exclusion reason: Wrong measure (e.g. loneliness not measured or identified as theme).

235. Tobin C, Murphy-Lawless J, Beck CT. Childbirth in exile: asylum seeking women's experience of childbirth in Ireland. Midwifery. 2014;30(7):831-8. Exclusion reason: Wrong focus of study.

236. Trad PV. On becoming a mother: In the throes of developmental transformation. Psychoanal Psychol. 1990;7(3):341-61. Exclusion reason: Wrong focus of study.

237. Ugarriza DN. Postpartum depressed women's explanation of depression. J Nurs Scholarsh. 2002;34(3):227-33. Exclusion reason: Wrong focus of study.

238. Ugarriza DN, Brown SE, Chang-Martinez C. Anglo-American mothers and the prevention of postpartum depression. Issues Ment Health Nurs. 2007;28(7):781-98. Exclusion reason: Wrong focus of study.

239. Valcarcel C, Jatziri M, Borbor J, Santiesteban Y. Quality of life of pregnant teens assisted in Junta de Beneficencia de Guayaquil Hospital. Ciencia Unemi. 2018;11(27):87-96. Exclusion reason: No English translation available.

240. Valdez CR, Martinez E. Mexican Immigrant Fathers' Recognition of and Coping with Maternal Depression: The Influence of Meaning-Making on Marital and Co-Parenting Roles among Men Participating in a Family Intervention. Journal of Latina/o Psychology. 2019;7(4):304-21. Exclusion reason: Wrong patient population.

241. Vasileiou K, Barnett J, Barreto M, Vines J, Atkinson M, Lawson S, et al. Experiences of Loneliness Associated with Being an Informal Caregiver: A Qualitative Investigation. Front Psychol. 2017;8:585. Exclusion reason: Wrong patient population.

242. Villecourt-Couchat I, di Vettimo DS. Entre honte et culpabilité, la capacité d’être seule de la mère en présence de son enfant = Between shame and guilt, the mother’s ability to be alone in her child’s presence. Dialogue: Recherches sur le couple et la famille. 2018;219(1):111-24. Exclusion reason: No English translation available.

243. Viveiros CJ, Darling EK. Perceptions of barriers to accessing perinatal mental health care in midwifery: A scoping review. Midwifery. 2019;70:106-18. Exclusion reason: Wrong focus of study.

244. Wakefield CE, McLoone JK, Butow P, Lenthen K, Cohn RJ. Parental adjustment to the completion of their child's cancer treatment. Pediatr Blood Cancer. 2011;56(4):524-31. Exclusion reason: Wrong patient population.

245. Walsh JH, Lutzker JR, Guastaferro KM, Whitaker DJ. A preliminary analysis of the relationship between hazards in the home and the potential for abuse with families at risk. International Journal of Child & Adolescent Health. 2016;9(3):355-65. Exclusion reason: Wrong focus of study.

246. Wells MB, Kerstis B, Andersson E. Impacted family equality, self-confidence and loneliness: a cross-sectional study of first-time and multi-time fathers’ satisfaction with prenatal and postnatal father groups in Sweden. Scand J Caring Sci. 2020. Exclusion reason: Wrong date.

247. Westrupp EM, Karantzas G, Macdonald JA, Olive L, Youssef G, Greenwood CJ, et al. Study Protocol for the COVID-19 Pandemic Adjustment Survey (CPAS): A Longitudinal Study of Australian Parents of a Child 0-18 Years. Frontiers in psychiatry Frontiers Research Foundation. 2020;11:555750. Exclusion reason: Wrong date.

248. Wigert H, Dellenmark Blom M, Bry K. Parents' experiences of communication with neonatal intensive-care unit staff: an interview study. BMC Pediatr. 2014;14:304. Exclusion reason: Wrong focus of study.

249. Yako EM, Yako JM. A descriptive study of the reasons and consequences of pregnancy among single adolescent mothers in Lesotho. Curationis. 2007;30(3):74-81. Exclusion reason: Wrong focus of study.

250. Yu Q, Mazzoni S, Lauzon M, Borgatti A, Caceres N, Miller S, et al. Associations Between Social Network Characteristics and Loneliness During Pregnancy in a Sample of Predominantly African American, Largely Publicly-Insured Women. Maternal & Child Health Journal. 2020;24(12):1429-37. Exclusion reason: Wrong date.

251. Yu QH, Miller S, Fata A, Caceres N, Mazzoni S, Salvy SJ. Relationship between Social Networks and Feelings of Loneliness during Pregnancy. Ann Behav Med. 2020;54:S380-S. Exclusion reason: Wrong publication type (commentary).

252. Zare N, Ravanipour M, Bahreini M, Motamed N, Hatami G, Nemati H. Effect of a Self-Management Empowerment Program on Anger and Social Isolation of Mothers of Children with Cerebral Palsy: A Randomized Controlled Clinical Trial. Journal of Evidence-based Care. 2017;7(3):35-44. Exclusion reason: Wrong patient population.

253. Zayas LH. Thematic features in the manifest dreams of expectant fathers. Clin Soc Work J. 1988;16(3):282-96. Exclusion reason: Wrong focus of study.

254. Zekeri AA. Livelihood strategies of food-insecure poor, female-headed families in rural Alabama. Psychol Rep. 2007;101(3 Pt 2):1031-6. Exclusion reason: Wrong patient population.
