# Supplementary material for: Loneliness in pregnant and postpartum people and parents of children aged 5 years or younger: a scoping review
Source: Syst Rev. 2022 Sep 7;11:196. doi: 10.1186/s13643-022-02065-5 (PMC9451126; doi:10.1186/s13643-022-02065-5)
Supplement: Supplementary file 1 — Additional file 1: Table 1. Summary of Documents Included. Information about the records included in this review, as well as additional details including study aims, designs of included studies, and characteristics of the studies’ samples. Table 2. Data Extracted on Parental Loneliness contains data related to the research questions of this scoping review, including what type of loneliness was identified (if authors addressed loneliness type), study results, definition of loneliness used (if authors defined loneliness), means for measuring loneliness (if loneliness was measured), factors associated with and protective of loneliness, and prevalence of loneliness within the study sample. [file 13643_2022_2065_MOESM1_ESM.zip › Included studies_108-ScR-Parental-LonelinessR4.docx]

Included Studies

1. Aching MC, Granato TMM. The good enough mother under social vulnerability conditions. Estudos de Psicologia. 2016;33(1):15-24.

2. Amphlett JE. Less than kin and more than kind: Maternal playgroup experience. 1998;59:1838-.

3. Arimoto A, Tadaka E. Reliability and validity of Japanese versions of the UCLA loneliness scale version 3 for use among mothers with infants and toddlers: a cross-sectional study. BMC Womens Health. 2019;19(1):105.

4. Armstrong MA. Being pregnant and using drugs: a retrospective phenomenological inquiry. 1992(D.N.SC.):231 p- p.

5. Aydin R, Korukcu O, Kabukcuoglu K. Investigation of the Experiences of Mothers Living Through Prenatal Loss Incidents: A Qualitative Study. J Nurs Res. 2019;27(3):e22.

6. Ayers S, Crawley R, Webb R, Button S, Thornton A, group HAc. What are women stressed about after birth? Birth. 2019;46(4):678-85.

7. Badaru UM, Ogwumike OO, Adeniyi AF, Kaka B. Psychosocial adversities and depression in mothers of children with cerebral palsy in Nigeria. J Pediatr Neurol. 2013;11(1):1-7.

8. Bandyopadhyay M, Small R, Watson LF, Brown S. Life with a new baby: how do immigrant and Australian-born women's experiences compare? Aust N Z J Public Health. 2010;34(4):412-21.

9. Beck CT. The lived experience of postpartum depression: a phenomenological study. Nurs Res. 1992;41(3):166-70.

10. Beck CT. Postpartum depression: a metasynthesis. Qual Health Res. 2002;12(4):453-72.

11. Bloom T, Glass N, Curry MA, Hernandez R, Houck G. Maternal stress exposures, reactions, and priorities for stress reduction among low-income, urban women. Journal of Midwifery & Women's Health. 2013;58(2):167-74.

12. Bondas-Salonen T. How women experience the presence of their partners at the births of their babies. Qual Health Res. 1998;8(6):784-800.

13. Botha E, Joronen K, Kaunonen M. The consequences of having an excessively crying infant in the family: an integrative literature review. Scand J Caring Sci. 2019;33(4):779-90.

14. Callan VJ, Hennessey JF. The psychological adjustment of women experiencing infertility. Br J Med Psychol. 1988;61(Pt 2):137-40.

15. Charter R, Ussher JM, Perz J, Robinson K. The transgender parent: Experiences and constructions of pregnancy and parenthood for transgender men in Australia. International Journal of Transgenderism. 2018;19(1):64-77.

16. Chiaradonna W. A group work approach to post-surrender treatment of unwed mothers. Social Work with Groups: A Journal of Community and Clinical Practice. 1982;5(4):47-68.

17. Childs RE. Maternal psychological conflicts associated with the birth of a retarded child. Matern Child Nurs J. 1985;14(3):175-82.

18. Co-operative G, British Red Cross S. Trapped in a bubble : an investigation into triggers for loneliness in the UK. London: Kantar Public=; 2016 [cited Included Available from: https://www.redcross.org.uk/about-us/what-we-do/action-on-loneliness.

19. Connelly JJ, Golding J, Gregory SP, Ring SM, Davis JM, Davey Smith G, et al. Personality, behavior and environmental features associated with OXTR genetic variants in British mothers. PLoS ONE [Electronic Resource]. 2014;9(3):e90465.

20. Cote-Arsenault D, Denney-Koelsch E. "My baby is a person": parents' experiences with life-threatening fetal diagnosis. J Palliat Med. 2011;14(12):1302-8.

21. Cronin C. First-time mothers – identifying their needs, perceptions and experiences. Journal of Clinical Nursing (Wiley-Blackwell). 2003;12(2):260-7.

22. Cutrona CE. DEPRESSIVE ATTRIBUTIONAL STYLE AND NONPSYCHOTIC POSTPARTUM DEPRESSION. 1981(8201082):275.

23. Cutrona CE. Objective determinants of perceived social support. J Pers Soc Psychol. 1986;50(2):349-55.

24. Dennis CL, Hodnett E, Kenton L, Weston J, Zupancic J, Stewart DE, et al. Effect of peer support on prevention of postnatal depression among high risk women: multisite randomised controlled trial. BMJ. 2009;338:a3064.

25. DiIorio C, Riley B. Predictors of loneliness in pregnant teenagers. Public Health Nurs. 1988;5(2):110-5.

26. Eissler LA, Toebe D. The experience of medically indicated relocation during high-risk pregnancy: A phenomenological study. 2002(1409947):56.

27. Ellis SA, Wojnar DM, Pettinato M. Conception, pregnancy, and birth experiences of male and gender variant gestational parents: it's how we could have a family. Journal of Midwifery & Women's Health. 2015;60(1):62-9.

28. Engnes K, Liden E, Lundgren I. Experiences of being exposed to intimate partner violence during pregnancy. International Journal of Qualitative Studies on Health and Well being. 2012;7.

29. Fords GM, Crowley T, van der Merwe AS. The lived experiences of rural women diagnosed with the human immunodeficiency virus in the antenatal period. SAHARA J: Journal of Social Aspects of HIV/AIDS Research Alliance. 2017;14(1):85-92.

30. Fry MJ, Cartwright DW, Huang RC, Davies MW. Preterm birth a long distance from home and its significant social and financial stress. Aust N Z J Obstet Gynaecol. 2003;43(4):317-21.

31. Garthus-Niegel S, Storksen HT, Torgersen L, Von Soest T, Eberhard-Gran M. The Wijma Delivery Expectancy/Experience Questionnaire: a factor analytic study. Journal of Psychosomatic Obstetrics & Gynecology. 2011;32(3):160-3.

32. Geller JS. Loneliness and pregnancy in an urban Latino community: associations with maternal age and unscheduled hospital utilization. Journal of Psychosomatic Obstetrics & Gynecology. 2004;25(3-4):203-9.

33. Goedecke DM, Jones E. A comparison of personal factors in pregnant and non-pregnant adolescent girls. 1991(1346408):101.

34. Harms VO, Abbott DA. The relationship of family functioning and self-perception to adolescent pregnancy: A cultural perspective. 1994(9425285):59.

35. Heaman M, Gupton A. Perceptions of bed rest by women with high-risk pregnancies: A comparison between home and hospital. Birth. 1998;25(4):252-8.

36. Hudson DB, Campbell-Grossman C, Kupzyk KA, Brown SE, Yates BC, Hanna KM. Social Support and Psychosocial Well-being Among Low-Income, Adolescent, African American, First-Time Mothers. Clin Nurse Spec. 2016;30(3):150-8.

37. Hudson DB, Elek SM, Campbell-Grossman C. Depression, self-esteem, loneliness, and social support among adolescent mothers participating in the new parents project. Adolescence. 2000;35(139):445-53.

38. Huttlinger KW. The experience of pregnancy in teenage girls. 1988(PH.D.):199 p- p.

39. Igarashi Y, Horiuchi S, Porter SE. Immigrants' experiences of maternity care in Japan. J Community Health. 2013;38(4):781-90.

40. Jabraeili M, Hassankhani H, Negarandeh R, Abbaszadeh M, Cleveland LM. Mothers' Emotional Experiences Providing Care for Their Infants Within the Culture of an Iranian Neonatal Unit. Adv Neonatal Care. 2018;18(4):E3-E12.

41. Jopling K, Sserwanja I. Loneliness Across the Life Course: A Rapid Review of the Evidence: Calouste Gulbenkian Foundation, UK Branch; 2016 [cited Included Available from: https://gulbenkian.pt/uk-branch/.

42. Jundt K, Haertl K, Knobbe A, Kaestner R, Friese K, Peschers UM. Pregnant women after physical and sexual abuse in Germany. Gynecol Obstet Invest. 2009;68(2):82-7.

43. Junttila N, Ahlqvist-Bjorkroth S, Aromaa M, Rautava P, Piha J, Raiha H. Intercorrelations and developmental pathways of mothers' and fathers' loneliness during pregnancy, infancy and toddlerhood--STEPS study. Scand J Psychol. 2015;56(5):482-8.

44. Junttila N, Ahlqvist-Björkroth S, Aromaa M, Rautava P, Piha J, Vauras M, et al. Mothers' and fathers' loneliness during pregnancy, infancy and toddlerhood. Psychology and Education: An Interdisciplinary Journal. 2013;50(3-4):98-104.

45. Kane AH. Loneliness in young mothers. Nurs Mirror. 1964;118:489-.

46. Khan S, Ion A, Alyass A, Greene S, Kwaramba G, Smith S, et al. Loneliness and perceived social support in pregnancy and early postpartum of mothers living with HIV in Ontario, Canada. AIDS Care. 2019;31(3):318-25.

47. Kjelsvik M, Sekse RJT, Moi AL, Aasen EM, Chesla CA, Gjengedal E. Women's experiences when unsure about whether or not to have an abortion in the first trimester. Health Care Women Int. 2018;39(7):784-807.

48. Klein TM. Adolescent pregnancy and loneliness. Public Health Nurs. 1998;15(5):338-47.

49. Knight A, Chase E, Aggleton P. 'Someone of your own to love': Experiences of being looked after as influences on teenage pregnancy. Children and Society. 2006;20(5):391-403.

50. Korukcu O, Bulut O, Kukulu K. Psychometric Evaluation of the Wijma Delivery Expectancy/Experience Questionnaire Version B. Health Care Women Int. 2016;37(5):550-67.

51. Kroupa SE, Carman RS. The interpersonal world of the pregnant adolescent: A multiple comparison group approach. 1990(9030609):226.

52. Kruse JA, Williams RA, Seng JS. Considering a Relational Model for Depression in Women with Postpartum Depression. International Journal of Childbirth. 2014;4(3):151-68.

53. LeDrew HM, Moores P, Read T, O'Regan-Hogan M. He's here and he's gone; he's here and he's gone ... The experiences of new mothers in rural Newfoundland and Labrador, Canada, whose partners work away from home. Rural & Remote Health. 2018;18(3):4542.

54. Lee K, Vasileiou K, Barnett J. 'Lonely within the mother': An exploratory study of first-time mothers' experiences of loneliness. J Health Psychol. 2019;24(10):1334-44.

55. Lee LC, Yin TJ, Yu S. Prenatal examination utilization and its determinants for immigrant women in Taiwan: an exploratory study. J Nurs Res. 2009;17(1):73-82.

56. Liu LL, Slap GB, Kinsman SB, Khalid N. Pregnancy among American Indian adolescents: reactions and prenatal care. J Adolesc Health. 1994;15(4):336-41.

57. Lundgren I, Berg M. Central concepts in the midwife-woman relationship. Scand J Caring Sci. 2007;21(2):220-8.

58. Lundqvist P, Weis J, Sivberg B. Parents' journey caring for a preterm infant until discharge from hospital-based neonatal home care-A challenging process to cope with. J Clin Nurs. 2019;28(15-16):2966-78.

59. Luoma I, Korhonen M, Puura K, Salmelin RK. Maternal loneliness: concurrent and longitudinal associations with depressive symptoms and child adjustment. Psychology Health & Medicine. 2019;24(6):667-79.

60. Lutz WJ, Hock E. Parental emotions following the birth of the first child: gender differences in depressive symptoms. Am J Orthopsychiatry. 2002;72(3):415-21.

61. Mandai M, Kaso M, Takahashi Y, Nakayama T. Loneliness among mothers raising children under the age of 3 years and predictors with special reference to the use of SNS: a community-based cross-sectional study. BMC Womens Health. 2018;18(1):131.

62. Martin BP. An analysis of common postpartum problems and adaptation strategies used by women during the first two to eight weeks following delivery of a fullterm healthy newborn. 1995(PH.D.):110 p- p.

63. Matos-Rios AY. Loneliness and intimacy of friendship among pregnant and nonpregnant adolescents. 1995(D.N.S.):125 p- p.

64. Mauthner NS, Stoppard JM, McMullen LM. 'Imprisoned in my own prison': A relational understanding of Sonya's story of postpartum depression. Situating sadness: Women and depression in social context. 2003:88-112.

65. Milner JS, Wimberley RC. Prediction and explanation of child abuse. J Clin Psychol. 1980;36(4):875-84.

66. Mommersteeg PM, Drost JT, Ottervanger JP, Maas AH. Long-term follow-up of psychosocial distress after early onset preeclampsia: the Preeclampsia Risk EValuation in FEMales cohort study. Journal of Psychosomatic Obstetrics & Gynecology. 2016;37(3):101-9.

67. Monti F, Mori GF. The 'times' of maternality. From pregnancy to motherhood: Psychoanalytic aspects of the beginning of the mother-child relationship. 2015:107-19.

68. Mossman SL. How to cure the home-alone blues... a new mother offers suggestions for coping with loneliness. American Baby. 1980;42:38-.

69. Mugweni L. Exploring prenatal health promotion experiences of recent immigrant women. 2009(MR63954):133.

70. Muller ME. The development and testing of the Mueller Prenatal Attachment Inventory. 1989(PH.D.):155 p- p.

71. Nadelson CC. The pregnant teenager: Problems of choice in a developmental framework. Psychiatric Opinion. 1975;12(2):6-12.

72. Nahas VL, Hillege S, Amasheh N. Postpartum depression. The lived experiences of Middle Eastern migrant women in Australia. J Nurse Midwifery. 1999;44(1):65-74.

73. Nasir R, Ahmad Zamani Z, Khairudin R, Wan Sulaiman WS, Mohd Sani MN, Amin AS. Depression, loneliness and cognitive distortion among young unwed pregnant women in Malaysia: Counseling implications. Asian Social Science. 2016;12(8):104-9.

74. Nilsson C, Lundgren I. Women's lived experience of fear of childbirth. Midwifery. 2009;25(2):e1-9.

75. Nims CL. Postpartum depression: The lived experience. 1997(1383715):72.

76. Nystrom K, Ohrling K. Parental support: mothers' experience of electronic encounters. J Telemed Telecare. 2006;12(4):194-7.

77. Olsson P, Jansson L, Norberg A. Parenthood as talked about in Swedish ante- and postnatal midwifery consultations. A qualitative study of 58 video-recorded consultations. Scand J Caring Sci. 1998;12(4):205-14.

78. Omer-Salim A, Suri S, Dadhich JP, Faridi MM, Olsson P. Theory and social practice of agency in combining breastfeeding and employment: A qualitative study among health workers in New Delhi, India. Women & Birth: Journal of the Australian College of Midwives. 2014;27(4):298-306.

79. Ornelas IJ, Perreira KM, Beeber L, Maxwell L. Challenges and strategies to maintaining emotional health: qualitative perspectives of Mexican immigrant mothers. Journal of Family Issues. 2009;30(11):1556-75.

80. Palmer L, Carlsson G, Brunt D, Nystrom M. Existential security is a necessary condition for continued breastfeeding despite severe initial difficulties: a lifeworld hermeneutical study. International Breastfeeding Journal. 2015;10:17.

81. Perlman D, Milardo RM. Loneliness: A life-span, family perspective. Families and social networks. 1988:190-220.

82. Pletsch PK. A DESCRIPTION AND COMPARISON OF HEALTH RELATED ACTIVITIES OF PREGNANT AND NONPREGNANT HIGH SCHOOL STUDENTS. 1984(8422539):125.

83. Proctor SE. Loneliness and childbearing in adolescence. 1996(D.N.S.):384 p- p.

84. Ritchie J. Social characteristics of a sample of solo mothers. N Z Med J. 1980;91(659):349-52.

85. Robbins JM, DeLamater JD. Support from significant others and loneliness following induced abortion. Soc Psychiatry. 1985;20(2):92-9.

86. Rokach A. Giving life: Loneliness, pregnancy, and motherhood. Social Behavior and Personality: An International Journal. 2004;32(7):691-702.

87. Rokach A. Coping with loneliness during pregnancy and motherhood. Psychology and Education: An Interdisciplinary Journal. 2005;42(1):1-12.

88. Rokach A. Self-perception of the antecedents of loneliness among new mothers and pregnant women. Psychol Rep. 2007;100(1):231-43.

89. Rolls C, Hanna B. What about the mother and family when an infant doesn't sleep? Australian Journal of Primary Health. 2001;7(3):49-53.

90. Russo A, Lewis B, Joyce A, Crockett B, Luchters S. A qualitative exploration of the emotional wellbeing and support needs of new mothers from Afghanistan living in Melbourne, Australia. BMC Pregnancy Childbirth. 2015;15:197.

91. Sable MR, Washington CC, Schwartz LR, Jorgenson M. Social well-being in pregnant women: intended versus unintended pregnancies. J Psychosoc Nurs Ment Health Serv. 2007;45(12):24-31.

92. Samano R, Martinez-Rojano H, Robichaux D, Rodriguez-Ventura AL, Sanchez-Jimenez B, de la Luz Hoyuela M, et al. Family context and individual situation of teens before, during and after pregnancy in Mexico City. BMC Pregnancy Childbirth. 2017;17(1):382.

93. Santos HP, Jr., Kossakowski JJ, Schwartz TA, Beeber L, Fried EI. Longitudinal network structure of depression symptoms and self-efficacy in low-income mothers. PLoS ONE [Electronic Resource]. 2018;13(1):e0191675.

94. Saunders T, Lawrence J. COMING FULL CIRCLE: BUILDING A SUSTAINABLE COMMUNITY OF MOTHERS. Practising Midwife. 2018;21(3):35-8.

95. Schuez-Havupalo L, Lahti E, Junttila N, Toivonen L, Aromaa M, Rautava P, et al. Parents' depression and loneliness during pregnancy and respiratory infections in the offspring: A prospective birth cohort study. PLoS ONE [Electronic Resource]. 2018;13(9):e0203650.

96. Shorey S, Chee CYI, Ng ED, Lau Y, Dennis CL, Chan YH. Evaluation of a Technology-Based Peer-Support Intervention Program for Preventing Postnatal Depression (Part 1): Randomized Controlled Trial. J Med Internet Res. 2019;21(8):e12410.

97. Smith JE. Prenatal maternal stress and coping among vulnerable rural young women. 2007(Ph.D.):139 p- p.

98. Sorenson DS. Healing traumatizing provider interactions among women through short-term group therapy. Arch Psychiatr Nurs. 2003;17(6):259-69.

99. Spinetta JJ. Parental personality factors in child abuse. J Consult Clin Psychol. 1978;46(6):1409-14.

100. Stewart M, Dennis CL, Kariwo M, Kushner KE, Letourneau N, Makumbe K, et al. Challenges Faced by Refugee New Parents from Africa in Canada. Journal of Immigrant & Minority Health. 2015;17(4):1146-56.

101. Stewart M, Kushner KE, Dennis C, Kariwo M, Letourneau N, Makumbe K, et al. Social support needs of Sudanese and Zimbabwean refugee new parents in Canada. International Journal of Migration, Health & Social Care. 2017;13(2):234-52.

102. Stewart M, Makwarimba E, Letourneau NL, Kushner KE, Spitzer DL, Dennis CL, et al. Impacts of a Support Intervention for Zimbabwean and Sudanese Refugee Parents: "I Am Not Alone". Can J Nurs Res. 2015;47(4):113-40.

103. Tuominen M, Junttila N, Ahonen P, Rautava P. The effect of relational continuity of care in maternity and child health clinics on parenting self-efficacy of mothers and fathers with loneliness and depressive symptoms. Scand J Psychol. 2016;57(3):193-200.

104. Van der Gucht N, Lewis K. Women's experiences of coping with pain during childbirth: a critical review of qualitative research. Midwifery. 2015;31(3):349-58.

105. Vicary JR, Corneal DA. A comparison of young women's psychosocial status based on age of their first childbirth. Fam Community Health. 2001;24(2):73-84.

106. Webber G, Wilson R. Childbirth in the north. A qualitative study in the Moose Factory zone. Can Fam Physician. 1993;39:781-8.

107. Yang YO, Peden-McAlpine C, Chen CH. A qualitative study of the experiences of Taiwanese women having their first baby after the age of 35 years. Midwifery. 2007;23(4):343-9.

108. Zaidi F, Nigam A, Anjum R, Agarwalla R. Postpartum Depression in Women: A Risk Factor Analysis. Journal of Clinical and Diagnostic Research JCDR. 2017;11(8):QC13-QC6.
