# Supplementary material for: Loneliness in pregnant and postpartum people and parents of children aged 5 years or younger: a scoping review
Source: Syst Rev. 2022 Sep 7;11:196. doi: 10.1186/s13643-022-02065-5 (PMC9451126; doi:10.1186/s13643-022-02065-5)
Supplement: Supplementary file 1 — Additional file 1: Table 1. Summary of Documents Included. Information about the records included in this review, as well as additional details including study aims, designs of included studies, and characteristics of the studies’ samples. Table 2. Data Extracted on Parental Loneliness contains data related to the research questions of this scoping review, including what type of loneliness was identified (if authors addressed loneliness type), study results, definition of loneliness used (if authors defined loneliness), means for measuring loneliness (if loneliness was measured), factors associated with and protective of loneliness, and prevalence of loneliness within the study sample. [file 13643_2022_2065_MOESM1_ESM.zip › Supplemental Table 1- Summary of Documents IncludedR4.docx]

**Supplemental Table 1: Summary of Documents Included in This Scoping Review**

| Authors, Year, Journal Information | Title | Country | Aim | Design | Sample (*n)* |
| --- | --- | --- | --- | --- | --- |
| Aching, M. C., & Granato, T. M. M. (2016). The good enough mother under social vulnerability conditions. *Estudos de Psicologia, 33*(1), 15-24. https://doi.org/10.1590/1982-02752016000100003 | The Good Enough Mother Under Social Vulnerability Conditions | Brazil | Psychoanalytical investigation of how a group of vulnerable mothers “construct the image of a good enough mother" (p. 17). | Qualitative Descriptive | Adult women (n = 17) who were staying in a shelter for women with their newborn babies in the city of Sāo Paulo, Brazil. |
| Amphlett, J. E. (1998). *Less than kin and more than kind: Maternal playgroup experience* (Publication Number 9832055) [Doctoral dissertation.] Massachusetts School of Professional Psychology. ProQuest Dissertations & Theses Global. | Less Than Kin and More Than Kind: Maternal Playgroup Experience | US | "This study seeks to illuminate and understand the holding experience of new mothers within the context of playgroups." (Holding as in Winnicottian nesting) "As such, it has two foci: the nature and function of playgroups as distinct support entities, and the subjective experience of participants, particularly in the context of the developmental transition to parenthood and the requisite support it requires (p. 7)" | Qualitative Phenomenology | 9 middle-class mothers who participated in a total of 16 playgroups. |
| Arimoto, A., & Tadaka, E. (2019). Reliability and validity of Japanese versions of the UCLA loneliness scale version 3 for use among mothers with infants and toddlers: A cross-sectional study. *BMC Women's Health, 19*(1), 105. https://doi.org/10.1186/s12905-019-0792-4 | Reliability And Validity of Japanese Versions of The UCLA Loneliness Scale Version 3 For Use Among Mothers With Infants And Toddlers: A Cross-sectional Study | Japan | "Our goal was to evaluate the reliability and validity of the Japanese version of the UCLA Loneliness Scale Version 3 (UCLA-LS3-J), as well as two short-form versions - the 10-item UCLA LS3 (SF-10) and the 3-item UCLA LS3 (SF-3) - for the measurement of loneliness in mothers with infants and toddlers in Japan. (abstract)" | Quantitative Cross-sectional | 430 mothers of infants and toddlers who visited a community health center in Yokohama City in Japan. |
| Armstrong, M. A. (1992). *Being pregnant and using drugs: A retrospective phenomenological inquiry* (Publication Number 9223368) [Doctoral dissertation.] University of San Diego. ProQuest Dissertations & Theses Global. | Being Pregnant and Using Drugs: A Retrospective Phenomenological Inquiry | US | “The purpose of the study was to explore the nature of women's experience and perception of the interaction, relationship, and intersection of the contextual phenomena of lifestyle, pregnancy, and substance abuse” (abstract). | Qualitative Phenomenology | 11 mothers participating in a recovery program. |
| Aydin, R., Korukcu, O., & Kabukcuoglu, K. (2019). Investigation of the experiences of mothers living through prenatal loss incidents: A qualitative study. *Journal of Nursing Research, 27*(3), e22. https://doi.org/10.1097/jnr.0000000000000289 | Investigation of the Experiences of Mothers Living Through Prenatal Loss Incidents: A Qualitative Study | Turkey | To investigate the experiences of women whose pregnancies were terminated because of medical indications (p. 1)." | Qualitative Descriptive | 10 women who were hospitalized and had subsequently experienced pregnancy loss during their hospitalization. |
| Ayers, S., Crawley, R., Webb, R., Button, S., Thornton, A., & group, H. A. C. (2019). What are women stressed about after birth? *Birth, 46*(4), 678-685. https://doi.org/10.1111/birt.12455 | What Are Women Stressed About After Birth? | UK | “This study aimed to identify what women find stressful during the early postpartum period in contemporary Western society” (p. 678). | Qualitative Descriptive | 148 women postpartum 6 to 12 weeks. |
| Badaru, U. M., Ogwumike, O. O., Adeniyi, A. F., & Kaka, B. (2013). Psychosocial adversities and depression in mothers of children with cerebral palsy in Nigeria. *Journal of Pediatric Neurology, 11*(1), 1-7. | Psychosocial Adversities and Depression in Mothers of Children with Cerebral Palsy in Nigeria | Nigeria | “This study was aimed at examining possible correlation of psychosocial adversities with depression in mothers of children with cerebral palsy (CP) in Nigeria” (p. 1). | Quantitative  Cross-sectional | 45 mothers of children with CP attending physiotherapy clinics. |
| Bandyopadhyay, M., Small, R., Watson, L. F., & Brown, S. (2010). Life with a new baby: How do immigrant and Australian-born women's experiences compare? *Australian and New Zealand Journal of Public Health, 34*(4), 412-421. https://doi.org/10.1111/j.1753-6405.2010.00575.x | Life with a New Baby: How Do Immigrant and Australian-Born Women's Experiences Compare? | Australia | “The aim of the analysis presented here is to explore similarities and differences in experiences of life with a young baby for Australian-born and immigrant women from non-English speaking countries who responded to a postal survey six months after giving birth as part of the evaluation of a large community trial of a range of strategies to improve the wellbeing of mothers of young babies," (p. 412). | Quantitative Cross-sectional | Six-months postpartum women born overseas in non-English speaking countries who reported speaking English very well (n = 460), born overseas in non-English-speaking countries who reported speaking English less than very well (n = 184), and Australian-born women (n = 9,796). |
| Beck, C. T. (1992). The lived experience of postpartum depression: A phenomenological study. *Nursing Research, 41*(3), 166-170. | The Lived Experience of Postpartum Depression: A Phenomenological Study | US | “The purpose of this phenomenological study was to describe the essential structure of the lived experience of postpartum depression (p. 166). | Qualitative Phenomenology | 7 mothers who had suffered from postpartum depression were interviewed regarding their subjective experiences. |
| Beck, C. T. (2002). Postpartum depression: A metasynthesis. *Qualitative Health Research*, 12(4),  453-472. https://doi.org/10.1177/104973202129120016 | Postpartum Depression: A Metasynthesis | US | The purpose was to “enlarge the interpretive possibilities of the results of these 18 individual studies and construct a larger narrative. It is hoped that these results can have a greater impact on direction for clinical practice if they are situated in a larger interpretive context," (p. 454). | Systematic Review | N/A |
| Bloom, T., Glass, N., Curry, M. A., Hernandez, R., & Houck, G. (2013). Maternal stress exposures, reactions, and priorities for stress reduction among low-income, urban women. *Journal of Midwifery & Women's Health, 58*(2), 167-174. https://doi.org/10.1111/j.1542-2011.2012.00197.x | Maternal Stress Exposures, Reactions, and Priorities for Stress Reduction Among Low-Income, Urban Women | US | “In this exploratory study, researchers aimed to describe maternal stress exposures, reactions, and the interrelationships among stressors during pregnancy in a sample of low-income, urban women, a population at high risk for maternal stress. A secondary aim was to better understand women's priorities for stress reduction during pregnancy. Specifically, we aimed to understand women's informal needs and to identify useful content for a stress-reduction information resource that health care providers might provide women, thereby laying the groundwork for future prenatal interventions," (p. 167-168). | Mixed-Methods | 24 low-income, urban women. |
| Bondas-Salonen, T. (1998). How women experience the presence of their partners at the births of their babies. *Qualitative Health Research, 8*(6), 784-800. | How Women Experience the Presence of Their Partners at the Births of Their Babies | Finland | “The purpose of this phenomenological study is to describe and explore Finnish women's experiences of their partners' presence at the births of their babies” (p. 785). | Qualitative Phenomenology | 40 Finnish women, each of whom had given birth to one to six children in different Finnish hospitals. |
| Botha, E., Joronen, K., & Kaunonen, M. (2019). The consequences of having an excessively crying infant in the family: An integrative literature review. *Scandinavian Journal of Caring Sciences, 33*(4), 779-790. https://doi.org/10.1111/scs.12702 | The Consequences of Having an Excessively Crying Infant in the Family: An Integrative Literature Review | Finland | “To identify, describe and synthesise previous studies on the consequences of having an excessively crying infant in the family” (p. 779). | Literature Review | N/A |
| Callan, V. J., & Hennessey, J. F. (1988). The psychological adjustment of women experiencing infertility. *British Journal of Medical Psychology, 61*(Pt 2), 137-140. | The Psychological Adjustment of Women Experiencing Infertility | Australia | “The aim of the study described here was to compare two groups of women currently attempting to achieve a pregnancy through in vitro fertilization (IVF). On the basis of previous studies, we expected mothers to report higher levels of adjustment” (p. 137). | Quantitative Cross-sectional | 53 infertile women and 24 mothers who now experienced infertility. |
| Charter, R., Ussher, J. M., Perz, J., & Robinson, K. (2018). The transgender parent: Experiences and constructions of pregnancy and parenthood for transgender men in Australia. *International Journal of Transgenderism, 19*(1), 64-77. https://doi.org/10.1080/15532739.2017.1399496 | The Transgender Parent: Experiences and Constructions of Pregnancy and Parenthood for Transgender Men in Australia | Australia | “The present study aims to address this gap in the literature through addressing the following research questions: how do Australian trans men construct and experience their desire for parenthood? And, how do Australian trans men construct and experience gestational pregnancy?” (p. 66). | Qualitative Descriptive | 25 transgender men between the ages of 25 to 46 years of age who had experienced a gestational pregnancy (p. 66). |
| Chiaradonna, W. (1982). A group work approach to post-surrender treatment of unwed mothers. *Social Work with Groups: A Journal of Community and Clinical Practice, 5*(4), 47-68. https://doi.org/10.1300/J009v05n04_05 | A Group Work Approach to Post-surrender Treatment of Unwed Mothers | US | To offer treatment to women post-surrender of a baby to understand their experiences and learn how to support this population. | Qualitative | 9 women ranging in age from their mid-teens to their mid-twenties who had surrendered their baby to adoption (6 in the first group and 3 in the second group). |
| Childs, R. E. (1985). Maternal psychological conflicts associated with the birth of a retarded child. *Maternal-Child Nursing Journal, 14*(3), 175-182. | Maternal Psychological Conflicts Associated with The Birth of a Retarded Child | US | “This study was conducted in order to learn more about the feelings of mothers after the birth of a retarded child. Special attention was focused on troublesome feelings that caused concern to the mother” (p. 176). | Qualitative Descriptive | 50 mothers of profoundly retarded children who had given birth to their child within the previous year. |
| Connelly, J. J., Golding, J., Gregory, S. P., Ring, S. M., Davis, J. M., Davey Smith, G., Harris, J. C., Carter, C. S., & Pembrey, M. (2014). Personality, behavior and environmental features associated with OXTR genetic variants in British mothers. *PLoS ONE* [Electronic Resource] 2014;9(3):e90465. DOI: [10.1371/journal.pone.0090465](https://dx.doi.org/10.1371/journal.pone.0090465) | Personality, Behavior and Environmental Features Associated with OXTR Genetic Variants in British Mothers | US and UK | “We use over 1000 environmental and phenotypic measures to assess possible relationships with two genetic variants (rs53576 and rs2254298) in a population sample of mothers using the Avon Longitudinal Study of Parents and Children (ALSPAC), and focus on two research questions: (a) do these women differ in their personality and emotional characteristics as described in the literature and (b) are there environmental differences in the women in the study associated with their polymorphisms on the SNPs rs53576 and/or rs2254298?” (p. 2). | Quantitative Correlational | More than 8000 pregnant women. |
| Cote-Arsenault, D., & Denney-Koelsch, E. (2011). "My baby is a person": Parents' experiences with life-threatening fetal diagnosis. *Journal of Palliative Medicine, 14*(12), 1302-1308. https://doi.org/10.1089/jpm.2011.0165 | My Baby is a Person": Parents' Experiences with Life-threatening Fetal Diagnosis | US | “Our goal was to explore the parents' experience of pregnancy with a lethal fetal diagnosis in order to gain insight into their needs. We sought to demonstrate the feasibility and acceptability of research participation of couples during this stressful period, so that more information, gathered by future studies in larger samples, can guide the formation of improved care programs in perinatal palliative care," (p. 1303). | Qualitative Descriptive | 8 parents (two women and two couples during pregnancy, and one couple was interviewed post-birth. |
| Cronin, C. (2003). First-time mothers – identifying their needs, perceptions and experiences. *Journal of Clinical Nursing (Wiley-Blackwell), 12*(2), 260-267. https://doi.org/10.1046/j.1365-2702.2003.00684.x | First-Time Mothers – Identifying Their Needs, Perceptions and Experiences | Ireland | “The aim of this research was to identify needs, perceptions and experiences of first-time mothers in the postnatal period," (p. 260). “…from the first few days at home, and up to nine months after birth” (p. 262). | Qualitative Descriptive | 13 women younger than 18 and up to age 25. |
| Cutrona, C. E. (1981). *Depressive attributional style and nonpsychotic postpartum depression*. (Publication Number 8201082) [Doctoral dissertation.] University of California, Los Angeles. ProQuest Dissertations & Theses Global. | Depressive Attributional Style and Nonpsychotic Postpartum Depression | US | “The primary purpose of the study was to test the reformulated learned helplessness model of depression in the context of postpartum adjustment” (p. xi). | Mixed Methods | 85 first-time mothers were followed from the third trimester of pregnancy through the second month after childbirth. |
| Cutrona, C. E. (1986). Objective determinants of perceived social support. *Journal of Personality and Social Psychology, 50*(2), 349-355. | Objective Determinants of Perceived Social Support | US | "The goal of the two studies reported here was to examine the relation between two fundamental social network variables and six components of perceived social support. The network variables that were chosen for investigation were network size and frequency of contact with network members" (p. 349). | Quantitative Descriptive | 71 women between 18 and 35 who had given birth to their first child approximately 12 months before. (50 older adults aged 60 to 88 were also studied so that the network characteristics and determinants of perceived availability of social support could be compared). |
| Dennis, Hodnett, E., Kenton, L., Weston, J., Zupancic, J., Stewart, D. E., & Kiss, A. (2009). Effect of peer support on prevention of postnatal depression among high risk women: Multisite randomised controlled trial. *BMJ*, 338(7689), 230–284. https://doi.org/10.1136/bmj.a3064 | Effect of Peer Support on Prevention of Postnatal Depression Among High Risk Women: Multisite Randomised Controlled Trial | Canada and US | “To evaluate the effectiveness of telephone based peer support in the prevention of postnatal depression” (p. 1). | Quantitative RCT | 701 women in the first two weeks postpartum identified as high risk for postnatal depression with the Edinburgh postnatal depression scale and randomised with an internet based randomization service," (p. 1). |
| DiIorio, C., & Riley, B. (1988). Predictors of loneliness in pregnant teenagers. *Public Health Nursing, 5*(2), 110-115. | Predictors of Loneliness in Pregnant Teenagers | US | “The purpose of this study was to examine the relationship of self-concept and future orientation to loneliness in a group of pregnant teenagers (p. 110). | Quantitative Descriptive | 79 pregnant teenagers under the age of 19. |
| Eissler, L. A. (2002). *The experience of medically indicated relocation during high-risk pregnancy: A phenomenological study* (Publication Number 1409947) [Master's thesis.] University of Alaska Anchorage. ProQuest Dissertations & Theses Global. | The Experience of Medically Indicated Relocation During High-risk Pregnancy: A Phenomenological Study | US | “The purpose of this study was to explore women's experiences of medically indicated relocation during high-risk pregnancies and to develop insight and understanding of this phenomenon” (p. 3). | Qualitative Phenomenology | 11 Alaskan women who had relocated during their pregnancy. |
| Ellis, S. A., Wojnar, D. M., & Pettinato, M. (2015). Conception, pregnancy, and birth experiences of male and gender variant gestational parents: It's how we could have a family. *Journal of Midwifery & Women's Health, 60*(1), 62-69. https://doi.org/10.1111/jmwh.12213 | Conception, Pregnancy, and Birth Experiences of Male and Gender Variant Gestational Parents: It's How We Could Have a Family | US | “The current study sought to begin filling the gap of knowledge by investigating the conception, pregnancy, and child-birth perspectives of male and gender-variant gestational parents who have undergone social or medical gender transition prior to pregnancy,” (p. 62). | Qualitative Grounded Theory | 8 male-identified or gender-variant gestational parents who had successfully delivered a baby within the past five years and had identified as male or gender variant at the time of conception and throughout the pregnancy. |
| Engnes, K., Liden, E., & Lundgren, I. (2012). Experiences of being exposed to intimate partner violence during pregnancy. *International Journal of Qualitative Studies on Health and Well being, 7*. https://doi.org/10.3402/qhw.v7i0.11199 | Experiences of Being Exposed to Intimate Partner Violence During Pregnancy | Norway | “The aim of the study was to gain a deeper understanding of women's experiences of being exposed to intimate partner violence (IPV) during pregnancy” (p. 1). | Qualitative Phenomenology | 5 Norwegian women aged 20 to 38, two during pregnancy and three after birth. |
| Fords, G. M., Crowley, T., & van der Merwe, A. S. (2017). The lived experiences of rural women diagnosed with the human immunodeficiency virus in the antenatal period. *SAHARA J: Journal of Social Aspects of HIV/AIDS Research Alliance, 14*(1), 85-92. https://doi.org/10.1080/17290376.2017.1379430 | The Lived Experiences of Rural Women Diagnosed with the Human Immunodeficiency Virus in the Antenatal Period | South Africa | “The aim of the study was to explore the lived experiences of women diagnosed with HIV in the antenatal period in a rural area in the Eastern Cape province of South Africa,” (p.85). | Qualitative Phenomenology | 10 women aged 24 to 46 were selected from the 199 women diagnosed with HIV in the antenatal period between January 2015 and July 2015. |
| Fry, M. J., Cartwright, D. W., Huang, R. C., & Davies, M. W. (2003). Preterm birth a long distance from home and its significant social and financial stress. *Australian and New Zealand Journal of Obstetrics and Gynaecology, 43*(4), 317-321. | Preterm Birth a Long Distance from Home and its Significant Social and Financial Stress | Australia | “The aim of the present paper was to determine the extent of this problem by determining the proportion of preterm infants admitted to the level three neonatal unit at the Royal Women’s Hospital (RWH) in Brisbane under these circum- stances. The other aim was to determine the circumstances of delivery of these preterm infants, and to determine the emotional, social, psychological and financial burden on the families” (p. 317). | Mixed-Methods | 12 mothers of preterm infants. |
| Garthus-Niegel, S., Storksen, H. T., Torgersen, L., Von Soest, T., & Eberhard-Gran, M. (2011). The Wijma Delivery Expectancy/Experience Questionnaire: A factor analytic study. *Journal of Psychosomatic Obstetrics & Gynecology, 32*(3), 160-163. https://doi.org/10.3109/0167482X.2011.573110 | The Wijma Delivery Expectancy/Experience Questionnaire: A Factor Analytic Study | Norway | “Our aim was therefore to investigate the underlying factor structure of the W-DEQ using data from a large-scale, representative sample of pregnant women. Moreover, we aimed to examine how the overall scale and potential sub factors were related to variables that are important for and characterize different domains of FOC, such as general anxiety, depression, prenatal attachment, and anticipations regarding the upcoming birth,” (p. 160). | Quantitative Cross-sectional | At 32 weeks of pregnancy, 1642 women scheduled to give birth at the Akershus University Hospital during 2009 to 2010 completed the questionnaires. |
| Geller, J. S. (2004). Loneliness and pregnancy in an urban Latino community: Associations with maternal age and unscheduled hospital utilization. *Journal of Psychosomatic Obstetrics & Gynecology, 25*(3-4), 203-209. | Loneliness and Pregnancy in an Urban Latino Community: Associations with Maternal Age and Unscheduled Hospital Utilization | US | “The objective is to compare loneliness in a pregnant population to a non-pregnant control group, and to evaluate loneliness and unscheduled hospital visits during pregnancy" (p. 203). "Our hypothesis, therefore, was that during pregnancy loneliness is a more prevalent issue in a woman's life than when not pregnant. If a pregnant woman felt insufficient support, then she might have more need for support from the medical community in the form of more unscheduled visits to the hospital" (p. 204). | Quantitative Correlational | 53 pregnant women in a predominantly Caribbean Latina population. |
| Goedecke, D. M., & Jones, E. (1991). *A comparison of personal factors in pregnant and non-pregnant adolescent girls* (Publication Number 1346408) [Master's thesis.] The University of Arizona. ProQuest Dissertations & Theses Global | A Comparison of Personal Factors in Pregnant and Non-Pregnant Adolescent Girls | US | “The purpose of this study was to compare the intrapersonal, and interpersonal factors among adolescent girls who were pregnant versus adolescent girls who were not pregnant" (p. 11). "The study sought to discover if there were differences in their feelings of closeness with family and friends, and if differences existed in their feelings of loneliness and sexual-self concept" (p.22). | Qualitative Descriptive | 34 pregnant and non-pregnant females aged 13 to 19. |
| Harms, V. O., & Abbott, D. A. (1994). *The relationship of family functioning and self-perception to adolescent pregnancy: A cultural perspective* (Publication Number 9425285) [Doctoral dissertation.] The University of Nebraska - Lincoln. ProQuest Dissertations & Theses Global. | The Relationship of Family Functioning and Self-perception to Adolescent Pregnancy: A Cultural Perspective | US | “This study focused on identifying the differences between adolescent females in Central America, those who were or are now pregnant and those who are not and have never been pregnant” (abstract). | Quantitative Descriptive | 53 adolescent females (29 pregnant, 24 non-pregnant). |
| Heaman, M., & Gupton, A. (1998). Perceptions of bed rest by women with high-risk pregnancies: A comparison between home and hospital. *Birth, 25*(4), 252-258. | Perceptions of Bed Rest by Women with High-risk Pregnancies: A Comparison Between Home and Hospital | Canada | An ethnographic examination of high-risk pregnant women's perspectives on bed rest at home versus the hospital. | Qualitative Ethnography | 24 pregnant women aged 18 to 36 who were on bed rest for at least seven days. |
| Hudson, D. B., Campbell-Grossman, C., Kupzyk, K. A., Brown, S. E., Yates, B. C., & Hanna, K. M. (2016). Social support and psychosocial well-being among low-income, adolescent, African American, first-time mothers. *Clinical Nurse Specialist, 30*(3), 150-158. https://doi.org/10.1097/NUR.0000000000000202 | Social Support and Psychosocial Well-Being Among Low-Income, Adolescent, African American, First-Time Mothers | US | “Aims of this study were to describe for single, low-income, adolescent, African American new mothers how (1) primary sources of social support changed over time; (2) the level of social support (emotional, informational, tangible, and problematic) from these primary sources changed over time; and (3) social support from the primary supporter was associated with mothers' psychosocial well-being (self-esteem and loneliness) over time” (p. 2). | Quantitative Correlation | 35 single, low-income, adolescent African American new mothers. |
| Hudson, D. B., Elek, S. M., & Campbell-Grossman, C. (2000). Depression, self-esteem, loneliness, and social support among adolescent mothers participating in the New Parents Project. *Adolescence, 35*(139), 445-453. | Depression, Self-esteem, Loneliness, and Social Support Among Adolescent Mothers Participating in the New Parents Project | US | “The purpose of this pilot study was to examine levels of depression, self-esteem, loneliness, and social support, and the relationships between these variables among adolescent mothers participating in the New Parents Project (formerly called the Young Parents Project)” (p. 446). | Quantitative Descriptive | 21 adolescent first-time mothers aged 16 to 19. |
| Huttlinger, K. W. (1988). *The experience of pregnancy in teenage girls* (Publication Number 8822424) [Doctoral dissertation.] University of Arizona. ProQuest Dissertations & Theses Global. | The Experience of Pregnancy in Teenage Girls | US | “The purpose, therefore, of this study was to investigate the experience of pregnancy in 16 teenage girls by using an ethnographic approach” (p. 13). | Qualitative Ethnography | 16 unmarried pregnant teenage females aged 14 to 19. |
| Igarashi, Y., Horiuchi, S., & Porter, S. E. (2013). Immigrants' experiences of maternity care in Japan. *Journal of Community Health, 38*(4), 781-790. https://doi.org/10.1007/s10900-013-9679-8 | Immigrants' Experiences of Maternity Care in Japan | Japan and US | “The primary purpose of this study was to analyze and compare evaluations from CDW and JP about the maternity care they received in Japan. The research question was: ``What are the associations among the quality of maternity care, loneliness, Japanese literacy level and care satisfaction for CDW" (p. 782). | Quantitative Descriptive Cross-sectional | 804 immigrant women living in Japan and 568 Japanese women. |
| Jabraeili, M., Hassankhani, H., Negarandeh, R., Abbaszadeh, M., & Cleveland, L. M. (2018). Mothers' emotional experiences providing care for their infants within the culture of an Iranian neonatal unit. *Advances in Neonatal Care, 18*(4), E3-E12. https://doi.org/10.1097/ANC.0000000000000530 | Mothers' Emotional Experiences Providing Care for Their Infants Within the Culture of An Iranian Neonatal Unit | Iran and US | “To explore the emotional caregiving experiences of mothers in an Iranian NU” (p. E3). | Qualitative Ethnography | 19 mothers of term and preterm infants aged 14 to 33 years. |
| Jopling, K., & Sserwanja, I. (2016). *Loneliness Across the Life Course: A Rapid Review of the Evidence*. Calouste Gulbenkian Foundation, UK Branch. https://gulbenkian.pt/uk-branch/ | Loneliness Across the Life Course: A Rapid Review of The Evidence | UK | “With this in mind, in this review we have sought to identify a small number of transitions around which it seems credible to construct a case that loneliness could be exacerbated or triggered, and have examined the extent to which this case is backed up by evidence. The transitions examined are by no means an exhaustive list of all of the transitions that may be of interest, however, it is intended to suggest some potential directions for future investigations" (p. 10). Note: Becoming a Parent is one of these transitional groups identified. | Literature Review | N/A |
| Jundt, K., Haertl, K., Knobbe, A., Kaestner, R., Friese, K., & Peschers, U. M. (2009). Pregnant women after physical and sexual abuse in Germany. *Gynecologic and Obstetric Investigation, 68*(2), 82-87. https://doi.org/10.1159/000215931 | Pregnant Women After Physical and Sexual Abuse in Germany | Germany | “The aim of our study was to evaluate the prevalence of physical and sexual abuse among pregnant women in Germany attending our antenatal outpatient clinic to observe whether a history of abuse had consequences for women's feelings about their pregnancy” (p. 82), and “whether their feelings about the pregnancy and the course of their pregnancy were different from those of non-abused women” (p. 83). | Quantitative Descriptive | 455 pregnant women. |
| Junttila, N., Ahlqvist-Bjorkroth, S., Aromaa, M., Rautava, P., Piha, J., & Raiha, H. (2015). Intercorrelations and developmental pathways of mothers' and fathers' loneliness during pregnancy, infancy and toddlerhood--STEPS study. *Scandinavian Journal of Psychology, 56*(5), 482-488. https://doi.org/10.1111/sjop.12241 | Intercorrelations and Developmental Pathways of Mothers' and Fathers' Loneliness During Pregnancy, Infancy and Toddlerhood--STEPS Study | Finland | Our aim was to study the longitudinal inter-correlations and development pathways of mothers’ and fathers’ social and emotional loneliness during pregnancy (20th pregnancy week), infancy, (child aged 8 months), and early childhood (child aged 18 months). Moreover, we aimed to study whether mothers and fathers who have different developmental profiles (identified by latent growth curve mixture models) differ in their experiences of marital dissatisfaction (RDAS), social phobia (SPIN) and depression (BDI) during pregnancy,” (p. 482 and 483). | Quantitative Correlational | At 20-weeks gestation, 1,234 mothers and 1,132 fathers; when the children were 8 months old, 1,273 mothers and 1,194 fathers; and when the children were 18 months old, 995 mothers and 879 fathers. |
| Junttila, N., Ahlqvist-Björkroth, S., Aromaa, M., Rautava, P., Piha, J., Vauras, M., Lagström, H., & Räihä, H. (2013). Mothers' and fathers' loneliness during pregnancy, infancy and toddlerhood. *Psychology and Education: An Interdisciplinary Journal, 50*(3-4), 98-104. http://search.ebscohost.com/login.aspx?direct=true&db=psyh&AN=2013-35445-010&site=ehost-live | Mothers' and Fathers' Loneliness During Pregnancy, Infancy and Toddlerhood | Finland | “Our first purpose was to study whether Finnish mothers' and father's social and emotional loneliness can be evaluated with the translated and modified version of the UCLA Loneliness Scale." "Our second purpose was to analyze the stability of mothers' and fathers' social and emotional (or global, in the case of a one-dimensional solution) loneliness during pregnancy (at the 20th pregnancy week), infancy (child aged 8 months), and early childhood (child aged 18 months)” (p. 99). | Quantitative Correlation | See Juntilla et al. 2015. |
| Kane, A. H. (1964). Loneliness in young mothers. *Nursing Mirror, 118*, 489-489. | Loneliness in Young Mothers | UK | Kane is reporting as a health visitor RN with a sociology background who argues that health visitors can help mothers of young children to overcome loneliness experienced during this time by facilitating introductions with other mothers with similar interests/needs. | Editorial | N/A |
| Kantar Public, Co-operative Group (Great Britain), & British Red Cross Society. (2016). *Trapped in a bubble: An investigation into triggers for loneliness in the UK*. Kantar Public=. https://www.redcross.org.uk/about-us/what-we-do/action-on-loneliness | Trapped in a Bubble: An Investigation into Triggers for Loneliness in the UK | UK | “There is much literature on loneliness later in life, but triggers for loneliness across life stages is less well documented. Therefore, the research concentrated on six target groups” (p. 6). See page 13 for a list of aims. | Multiple methods; Qualitative interviews with 115 people from the six groups of interest plus a quantitative survey of 2,523 people aged 16 and older. | “Young new mums (aged 18-24)” (p. 6). |
| Khan, S., Ion, A., Alyass, A., Greene, S., Kwaramba, G., Smith, S., Carvalhal, A., Kennedy, V. L., Walmsley, S., & Loutfy, M. (2019). Loneliness and perceived social support in pregnancy and early postpartum of mothers living with HIV in Ontario, Canada. *AIDS Care, 31*(3), 318-325. https://doi.org/10.1080/09540121.2018.1515469 | Loneliness and Perceived Social Support in Pregnancy and Early Postpartum of Mothers Living With HIV in Ontario, Canada | Canada | “The present quantitative analysis explores the clinical determinants of loneliness and lower perceived social support in pregnant and postpartum WLWHIV in Ontario. We also aim to describe the dynamic nature of these two outcomes and their determinants over time; pre-partum compared to post-partum. The hypothesized clinical determinants included continuous variables; age, years living with HIV, racism (Everyday Discrimination Scale) (Clark, Coleman, & Novak, 2004), depression score [Edinburgh Postnatal Depression Scale (EPDS)] (Cox, Holden, & Sagovsky, 1987) and categorical variables; nadir CD4 (,200 cells/uL), tertiary HIV care vs. community HIV care, and marital status" (p. 319). | Quantitative Descriptive | 15 WLWHIV who were 18 or older, biologically female and pregnant. |
| Kjelsvik, M., Sekse, R. J. T., Moi, A. L., Aasen, E. M., Chesla, C. A., & Gjengedal, E. (2018). Women's experiences when unsure about whether or not to have an abortion in the first trimester. *Health Care for Women International, 39*(7), 784-807. https://doi.org/10.1080/07399332.2018.146594 | Women's Experiences When Unsure About Whether or Not to Have an Abortion in the First Trimester | Norway and Canada | "In order to contribute to filling the gap in the literature and thereby improving health professionals' understanding and competence when caring for ambivalent pregnant women, the aim of this study was to explore the experiences of the subset of Norwegian pregnant women, who during the decision-making process still were unsure about whether to complete or terminate pregnancy" (p. 787). | Qualitative Phenomenology | 13 Norwegian women in their first trimester of pregnancy and considering abortion. |
| Klein, T. M. (1998). Adolescent pregnancy and loneliness. *Public Health Nursing, 15*(5), 338-347. | Adolescent Pregnancy and Loneliness | USA | “The purpose of this study was to describe the relationships between and among characterological loneliness and situational loneliness in childbearing adolescents" (p. 340). | Quantitative Cross Sectional | 57 pregnant adolescents between 12 and 21 years of age. |
| Knight, A., Chase, E., & Aggleton, P. (2006). 'Someone of your own to love': Experiences of being looked after as influences on teenage pregnancy. *Children and Society, 20*(5), 391-403. https://doi.org/10.1111/j.1099-0860.2006.00014.x | Someone of Your Own to Love': Experiences of Being Looked After as Influences on Teenage Pregnancy | UK | This article offers insights learned from the larger study it was connected to called Pregnancy and parenthood among young people in and leaving local authority care: Determinants and support for the mother, father and child. The insights are about how young peoples' experiences before and during their time in care may influence the choices they make about sexual relationships and pregnancy. | Qualitative Descriptive | 63 recently looked after people aged 15-24 (47 young women between the ages of 15 and 22, and 16 young men between the ages of 15-24). |
| Korukcu, O., Bulut, O., & Kukulu, K. (2016). Psychometric evaluation of the Wijma Delivery Expectancy/Experience Questionnaire Version B. *Health Care for Women International, 37*(5), 550-567. https://doi.org/10.1080/07399332.2014.943838 | Psychometric Evaluation of The Wijma Delivery Expectancy/Experience Questionnaire Version B | Turkey and US | “The authors aim is to examine the overall psychometric quality of the Wijma Delivery Expectancy/Experience Questionnaire version B.``''An additional aim is to contribute to the national literature regarding the performance of the instrument in cross-cultural adaptation" (p. 554). | Quantitative Descriptive | 227 mothers in an antenatal clinic in Turkey 24 hours after delivery. |
| Kroupa, S. E., & Carman, R. S. (1990). *The interpersonal world of the pregnant adolescent: A multiple comparison group approach* (Publication Number 9030609) [Doctoral dissertation.] University of Wyoming. | The Interpersonal World of The Pregnant Adolescent: A Multiple Comparison Group Approach | US | “The present study focuses on the subjective, interpersonal world of the pregnant, unmarried teenager” (p. 45). | Quantitative Descriptive Cross Sectional | 221 participants consisting of (31) pregnant (and 40 non-pregnant control) adolescent women between the ages of 13 and 19, (37) pregnant adult women between the ages of 20-29, a never-pregnant delinquent group (87), and a delinquent adolescents with previous pregnancy group (26). |
| Kruse, J. A., Williams, R. A., & Seng, J. S. (2014). Considering a relational model for depression in women with postpartum depression. *International Journal of Childbirth, 4*(3), 151-168. | Considering a Relational Model for Depression in Women with Postpartum Depression | US | “The purpose of this research study was to examine the multiple variables of perceived social support, sense of belonging, conflict in relationships, parenting sense of competence, maternal bonding with the infant, and loneliness for their associations in a relational model for depression with women experiencing PPD," (pp. 2-3). | Quantitative Correlation | 564 women at three time points ending at 6 weeks postpartum. |
| LeDrew, H. M., Moores, P., Read, T., & O'Regan-Hogan, M. (2018). He's here and he's gone; he's here and he's gone ... The experiences of new mothers in rural Newfoundland and Labrador, Canada, whose partners work away from home. *Rural & Remote Health, 18*(3), 4542. https://doi.org/10.22605/RRH4542 | He's Here and He's Gone; He's Here and He's Gone ... The Experiences of New Mothers in Rural Newfoundland and Labrador, Canada, Whose Partners Work Away from Home | Canada | “As early days of mothering may be consumed with anxiety and compounded by limited family support and absentee or intermittently present fathers it is critical for CHNs to understand the impact of ERM on families and to strive to identify and meet support needs. This article reports findings from a qualitative research study conducted from 2014 to 2015" (p. 2). "The purpose of this study was to understand the experiences of new mothers on the island portion of rural NL whose partners participated in ERM" (p. 3). | Qualitative Phenomenology | 19 women with children aged three or younger who lived on the island portion of rural NL, and whose partners worked away from home more than 30% of the calendar year. |
| Lee, K., Vasileiou, K., & Barnett, J. (2019). 'Lonely within the mother': An exploratory study of first-time mothers' experiences of loneliness. *Journal of Health Psychology, 24*(10), 1334-1344. https://doi.org/10.1177/1359105317723451 | 'Lonely Within the Mother': An Exploratory Study of First-time Mothers' Experiences of Loneliness | UK | “This study aims to add to the literature about loneliness in new mothers by providing an in-depth understanding of their experiences. Specifically, the study has two research objectives: To understand the context of first-time mothers' experiences of loneliness; To understand whether their accounts highlight discrepancies between expected and actual motherhood (e.g. in relation to their experiences and emotions), and if so, whether these gave rise to feelings of loneliness" (pp. 1335-1336). | Qualitative Phenomenology | 7 participants without a diagnosis of postpartum depression (to avoid undue distress and to maintain a focus on experiences of loneliness) and children aged between 4 and 9 months. |
| Lee, L. C., Yin, T. J., & Yu, S. (2009). Prenatal examination utilization and its determinants for immigrant women in Taiwan: An exploratory study. *Journal of Nursing Research, 17*(1), 73-82. https://doi.org/10.1097/JNR.0b013e3181999ee8 | Prenatal Examination Utilization and its Determinants for Immigrant Women in Taiwan: An Exploratory Study | Taiwan | "The purpose of this study was to understand and explore determinants of prenatal examination utilization among Vietnamese immigrant women in Taiwan” (p. 74). | Quantitative Cross Sectional | 101 primiparous Vietnamese women of childbearing age residing in northern Taiwan. |
| Liu, L. L., Slap, G. B., Kinsman, S. B., & Khalid, N. (1994). Pregnancy among American Indian adolescents: Reactions and prenatal care. *Journal of Adolescent Health, 15*(4), 336-341. | Pregnancy Among American Indian Adolescents: Reactions and Prenatal Care | US | The objective of this study was to explore the reactions and prenatal care of Navajo and Apache adolescents delivering infants at Shiprock Indian Hospital in New Mexico and Whiteriver Indian Hospital in Arizona. | Quantitative Descriptive Cross Sectional | 15 Navajo and 5 Apache participants aged 19 years old or younger who were interviewed within 24 hours after delivery. |
| Lundgren, I., & Berg, M. (2007). Central concepts in the midwife-woman relationship. *Scandinavian Journal of Caring Sciences, 21*(2), 220-228. | Central Concepts in the Midwife-Woman Relationship | Sweden | “The aim of this study was to delineate central concepts in the midwife-woman relationship, in normal as well as high-risk situations” (abstract). | Secondary Analysis of Phenomenological-hermeneutic Qualitative Interviews | 96 participants in eight studies |
| Lundqvist, P., Weis, J., & Sivberg, B. (2019). Parents' journey caring for a preterm infant until discharge from hospital-based neonatal home care-A challenging process to cope with. *Journal of Clinical Nursing, 28*(15-16), 2966-2978. https://doi.org/10.1111/jocn.14891 | Parents' Journey Caring for a Preterm Infant Until Discharge from Hospital-Based Neonatal Home Care- A Challenging Process to Cope With | Denmark and Sweden | "To present parents' experience of having a preterm infant cared for at the neonatal unit until discharge from hospital-based neonatal home care" (abstract). | Qualitative Phenomenology | 6 parents of infants born before 37 weeks gestation couples at a NICU in Sweden. |
| Luoma, I., Korhonen, M., Puura, K., & Salmelin, R. K. (2019). Maternal loneliness: Concurrent and longitudinal associations with depressive symptoms and child adjustment. *Psychology Health & Medicine, 24*(6), 667-679. https://doi.org/10.1080/13548506.2018.1554251 | Maternal Loneliness: Concurrent and Longitudinal Associations with Depressive Symptoms and Child Adjustment | Finland | “Our hypotheses were the following: 1) maternal loneliness is significantly related to maternal depressive symptoms both simultaneously and longitudinally at the three stages of motherhood examined; 2) maternal loneliness predicts internalizing problems in the child; and 3) maternal loneliness predicts the child's internalizing problems even when maternal depressive symptoms are controlled for" (p. 669). | Quantitative Correlational | 122 mothers expecting their first child were part of a larger longitudinal study and were available for data collection at all three stages (pregnancy, child aged 8-9, and child aged 16-17. |
| Lutz, W. J., & Hock, E. (2002). Parental emotions following the birth of the first child: Gender differences in depressive symptoms. *American Journal of Orthopsychiatry, 72*(3), 415-421. | Parental Emotions Following the Birth of the First Child: Gender Differences in Depressive Symptoms | US | “The study focuses on employment and marital quality as well as trait-like personality attributes that may put men and women at risk for depressive disorders during the first 2 years of parenthood. The primary aim is to examine gender differences in the relative contribution of employment, martial quality, and basic personality characteristics to depressive symptomatology" (p. 415). | Quantitative Descriptive | 107 married couples with infants between the ages of 22 and 25 months. |
| Mandai, M., Kaso, M., Takahashi, Y., & Nakayama, T. (2018). Loneliness among mothers raising children under the age of 3 years and predictors with special reference to the use of SNS: A community-based cross-sectional study. *BMC Women's Health, 18*(1), 131. https://doi.org/10.1186/s12905-018-0625-x | Loneliness Among Mothers Raising Children Under the Age of 3 Years and Predictors With Special Reference to the Use of SNS: A Community-Based Cross-Sectional Study | Japan | "This study aimed to identify predictors of loneliness in mothers raising children in Japan, with special reference to SNS use" (p.2). | Quantitative Cross Sectional | 523 mothers raising children under the age of three who resided in Nagahama City and accessed health check-ups for their children in the city. |
| Martin, B. P. (1995). *An analysis of common postpartum problems and adaptation strategies used by women during the first two to eight weeks following delivery of a fullterm healthy newborn* (Publication Number 9536488) [Doctoral dissertation.] University of Mississippi. ProQuest Dissertations & Theses Global. | An Analysis of Common Postpartum Problems and Adaptation Strategies Used by Women During the First Two to Eight Weeks Following Delivery of a Fullterm Healthy Newborn | USA | "The purpose of this research study was to identify common postpartum problems and adaptation strategies used to cope with these problems by postpartum women during the first 2 to 8 weeks following delivery of a fullterm healthy newborn” (abstract). | Quantitative Descriptive | 300 women between 2 and 8 weeks postpartum following the delivery of a fullterm healthy newborn. |
| Matos-Rios, A. Y. (1995). *Loneliness and intimacy of friendship among pregnant and nonpregnant adolescents* (Publication Number 9542142) [Doctoral dissertation.] Louisiana State University. ProQuest Dissertations & Theses Global. | Loneliness and Intimacy of Friendship Among Pregnant and Nonpregnant Adolescents | US | “The purpose of this study was to explore the presence of loneliness and to ascertain whether there is a relationship between friendship and loneliness among pregnant and non-pregnant teenagers” (abstract). | Quantitative Descriptive | 42 pregnant and 39 non pregnant teenagers ranging between the ages of 14-19. |
| Mauthner, N. S., Stoppard, J. M., & McMullen, L. M. (2003). 'Imprisoned in my own prison': A relational understanding of Sonya's story of postpartum depression. *Situating sadness: Women and depression in social context.*, 88-112. | ‘Imprisoned in my own Prison': A Relational Understanding of Sonya's Story of Postpartum Depression | US and UK | Case study guided by relational-psychology theory. "A relational approach opened up a space for me to theorize postnatal depression as a relational problem involving interpersonal and cultural ‘disconnections.’ Depressed women feel their experiences of motherhood are not reflected in other mothers' experiences or cultural representations of motherhood. Their sense of difference and deviance leaves them feeling cut off from the world and unable to confide their emotions in other people," (pp. 91-92). | Qualitative Case Study | One mother in her late thirties with postpartum depression. Mother of an only child. |
| Milner, J. S., & Wimberley, R. C. (1980). Prediction and explanation of child abuse. *Journal of Clinical Psychology, 36*(4), 875-884. | Prediction and Explanation of Child Abuse | US | “The purpose of the present study was to provide additional substantiation of the ability of the CAP-Inventory to differentiate abusers from nonusers using a larger, more heterogeneous sample. The study also attempted to delineate further the personality traits that are most descriptive of the abusive individuals.” (p. 875). | Quantitative Descriptive | 130 parents were administered the test instrument. 27 abusing and 27 matched non-abusing parents from across the state of North Carolina, and 38 abusing and non-abusing matched parents from Tulsa, OK. |
| Mommersteeg, P. M., Drost, J. T., Ottervanger, J. P., & Maas, A. H. (2016). Long-term follow-up of psychosocial distress after early onset preeclampsia: The Preeclampsia Risk EValuation in FEMales cohort study. *Journal of Psychosomatic Obstetrics & Gynecology, 37*(3), 101-109. <https://doi.org/10.3109/0167482X.2016.1168396> | Long-Term Follow-Up of Psychosocial Distress After Early Onset Preeclampsi: The Preeclampsia Risk EValuation in FEMales Cohort Study | Netherlands | The objective was “to examine long-term psychosocial distress in women with a history of early onset preeclampsia (PE) compared to a comparison group” (abstract). | Quantitative Correlation | All (n=681) participants of the original PREVFEM historical cohort study were invited to participate in the follow-up arm to examine psychosocial distress; in total, 265 women in the PE group responded, and 268 women in the non-PE group responded. |
| Monti, F., & Mori, G. F. (2015). The 'times' of maternality. From pregnancy to motherhood: Psychoanalytic aspects of the beginning of the mother-child relationship*.*, 107-119. | From Pregnancy to Motherhood: Psychoanalytic Aspects of the Beginning of the Mother-Child Relationship | Unknown | “Our study group, starting from Gina Ferrara Mori's project, began exploring "the territory of the dawn of the mother-child relationships", using as maps the "stories" narrated by "expectant" women and, as the journey tool (Bick, 1964), the observation of the emotional atmosphere" (p. 107). | Psychoanalytic Narrative | N/A |
| Mossman, S. L. (1980). How to cure the home-alone blues... A new mother offers suggestions for coping with loneliness. *American Baby, 42*, 38-38. | How To Cure the Home-alone Blues... A New Mother Offers Suggestions for Coping with Loneliness | US | First-person account of "home-alone blues" from a first-time mother who had been a working professional up to that point. Published in American Baby. | Editorial | N/A |
| Mugweni, L. (2009). *Exploring prenatal health promotion experiences of recent immigrant women* (Publication Number MR63954) [Master's thesis.] University of Manitoba (Canada). ProQuest Dissertations & Theses Global. | Exploring Prenatal Health Promotion Experiences of Recent Immigrant Women | Canada | “The purpose of this practicum was to explore the prenatal health promotion needs of immigrant women in Winnipeg” (p. 40). | Qualitative Descriptive | 5 immigrant women who had given birth to a child and lived in Winnipeg for less than five years. |
| Muller, M. E. (1989). *The development and testing of the Mueller Prenatal Attachment Inventory* (Publication Number 8926411) [Doctoral dissertation.] University of California, San Francisco,. ProQuest Dissertations & Theses Global. | The Development and Testing of the Mueller Prenatal Attachment Inventory | US | “The purpose of this study was to develop the Müller Prenatal Attachment Inventory (MPAI), to measure fetal attachment and demonstrate evidence of reliability and construct and criterion validity for the PAI” (p. 8). | Quantitative Descriptive Cross-sectional | 310 low risk, pregnant women. |
| Nadelson, C. C. (1975). The pregnant teenager: Problems of choice in a developmental framework. *Psychiatric Opinion, 12*(2), 6-12. | The Pregnant Teenager: Problems of Choice in a Developmental Framework | US | Special counseling considerations for the pregnant teenager are examined, including considerations for loneliness experienced by this group. | Editorial | N/A |
| Nahas, V. L., Hillege, S., & Amasheh, N. (1999). Postpartum depression- The lived experiences of Middle Eastern migrant women in Australia. *Journal of Nurse-Midwifery, 44*(1), 65-74. | Postpartum Depression-The Lived Experiences of Middle Eastern Migrant Women in Australia | Hong Kong and Australia | “The aim of this study was to explore the lived experiences of postpartum depression among the Middle Eastern women living in Sydney, Australia” (abstract). | Qualitative Phenomenology | 45 Middle Eastern women who had experienced PPD and who were recruited using the snowballing technique. Two criteria for inclusion in the sample were: 1) the woman had experienced PPD and 2) the woman was able to articulate her experiences with PPD. The selection of the sample was limited to Middle Eastern women living within the Western Sydney Area region. The women have lived in Sydney for the last 5 years and reported experiencing postpartum depression only during their first pregnancy in Sydney. |
| Nasir, R., Ahmad Zamani, Z., Khairudin, R., Wan Sulaiman, W. S., Mohd Sani, M. N., & Amin, A. S. (2016). Depression, loneliness and cognitive distortion among young unwed pregnant women in Malaysia: Counseling implications. *Asian Social Science*, *12*(8):104-109. DOI: [10.5539/ass.v12n8p104](https://dx.doi.org/10.5539/ass.v12n8p104) | Depression, Loneliness and Cognitive Distortion Among Young Unwed Pregnant Women in Malaysia: Counseling Implications | Malaysia | “The objective of this quantitative study is to ascertain whether relationships exist between depression, loneliness and cognitive distortion among unwed pregnant young women in Malaysia” (abstract). | Quantitative Descriptive | 150 young unwed pregnant women aged 14-29 years, living in shelters for unwed pregnant women in Malaysia. |
| Nilsson, C., & Lundgren, I. (2009). Women's lived experience of fear of childbirth. *Midwifery, 25*(2), e1-9. | Women's Lived Experience of Fear of Childbirth | Sweden | The objective is “to describe women's lived experience of fear of childbirth” (abstract). | Qualitative Phenomenology | 8 pregnant women (24-37 weeks gestation aged 27 to 34 years old) with severe fear of childbirth. |
| Nims, C. L. (1997). *Postpartum depression: The lived experience* (Publication Number 1383715) [Master's thesis.] Medical College of Ohio. ProQuest Dissertations & Theses Global. | Postpartum Depression: The Lived Experience | US | To use a phenomenological approach to achieve a “deepened insight into the unique experience of postpartum depression and to provide an understanding of women's perceptions of this phenomenon” (p. 5). | Qualitative Phenomenology | 4 women between the ages of 29 to 36 who had delivered a child within the past five years and who were currently attending a postpartum support group. |
| Nystrom, K., & Ohrling, K. (2006). Parental support: Mothers' experience of electronic encounters. *Journal of Telemedicine and Telecare, 12*(4), 194-197. | Parental Support: Mothers' Experience of Electronic Encounters | Sweden | Because northern Sweden consists of sparsely populated areas, a Telehealth study was conducted to provide virtual meetings for mothers with the intent of providing parental support. “The aim of the present paper was to describe the mothers' experience of the intervention.” (p. 194). | Qualitative Descriptive, Longitudinal Group Interviews | 5 mothers aged 20 to 32 who spoke Swedish, had a normal pregnancy and delivered a healthy child, and had access to a computer with broadband connection at home. |
| Olsson, P., Jansson, L., & Norberg, A. (1998). Parenthood as talked about in Swedish ante- and postnatal midwifery consultations. A qualitative study of 58 video-recorded consultations. *Scandinavian Journal of Caring Sciences, 12*(4), 205-214. https://www.ncbi.nlm.nih.gov/pubmed/10067646 | Parenthood as Talked About in Swedish Ante- And Postnatal Midwifery Consultations. A Qualitative Study of 58 Video-recorded Consultations | Sweden | The aims of this study were to describe topics of conversation concerning parenthood and to illuminate the meaning of being a mother and a father as disclosed in Swedish ante- and postnatal midwifery consultations, | Qualitative- Hermeneutics Phenomenology | 5 midwives, 5 pregnant women and 3 of the male partners. The parents were between the ages of 20 and 27. Two couples were expecting their first child, two couples were expecting their second child, and one couple was expecting their third child. |
| Omer-Salim, A., Suri, S., Dadhich, J. P., Faridi, M. M., & Olsson, P. (2014). Theory and social practice of agency in combining breastfeeding and employment: A qualitative study among health workers in New Delhi, India. *Women & Birth: Journal of the Australian College of Midwives, 27*(4), 298-306. https://doi.org/10.1016/j.wombi.2014.07.002 | Theory and Social Practice of Agency in Combining Breastfeeding and Employment: A Qualitative Study Among Health Workers on New Delhi, India | Sweden and India | “The aim of this study was thus to explore manifestations of agency in combining breastfeeding and employment amongst Indian health workers using Bandura’s theoretical constructs of agency and women’s experiences” (p. 304). | Qualitative Descriptive | 10 women who were first-time mothers, working within healthcare settings, and had a baby between 8 to 12 months of age. |
| Ornelas, I. J., Perreira, K. M., Beeber, L., & Maxwell, L. (2009). Challenges and strategies to maintaining emotional health: qualitative perspectives of Mexican immigrant mothers. *Journal of Family Issues, 30*(11), 1556-1575. | Challenges and Strategies to Maintaining Emotional Health: Qualitative Perspectives of Mexican Immigrant Mothers | US | "The purpose of the study was to identify factors that influence the emotional health of Mexican immigrant mothers in a new immigrant destination. In addition to identifying the stressors associated with developing depressive symptoms, we sought to describe the coping strategies women employed in the face of these stressors, including the role of social networks and social support” (p. 1558). | Qualitative Descriptive | 20 low-income, Mexican-born mothers of infants or toddlers living in North Carolina. |
| Palmer, L., Carlsson, G., Brunt, D., & Nystrom, M. (2015). Existential security is a necessary condition for continued breastfeeding despite severe initial difficulties: A lifeworld hermeneutical study. *International Breastfeeding Journal, 10*, 17. https://doi.org/10.1186/s13006-015-0042-9 | Existential Security Is a Necessary Condition for Continued Breastfeeding Despite Severe Initial Difficulties: A Lifeworld Hermeneutical Study | Sweden | “…this study aims to explain and understand how women’s decisions to continue or cease breastfeeding are influenced by the meaning of severe initial difficulties” (p. 2). | Qualitative Hermeneutics | 8 mothers who experienced severe difficulties with initial breastfeeding. |
| Perlman, D., & Milardo, R. M. (1988). Loneliness: A life-span, family perspective. *Families and social networks.*, 190-220. | Loneliness: A Life-span, Family Perspective | US | This is chapter 8 in the book Families and social networks. "The purpose of this chapter is to chart some of the fluctuations in loneliness that occur in conjunction with human development and the family life cycle" (p. 190). | Systematic Review | N/A |
| Pletsch, P. K. (1984). *A Description and Comparison of Health Related Activities of Pregnant and Nonpregnant High School Students* (Publication Number 8422539) [Doctoral dissertation.] University of Illinois at Chicago. ProQuest Dissertations & Theses Global. | A Description and Comparison of Health Related Activities of Pregnant and Nonpregnant High School Students | US | "The purpose of this project was to describe and analyze particular health activities of a special group of pregnant women as a beginning step in understanding health actions during pregnancy. More specifically, this project examined health related activities of urban inner city pregnant adolescents who had remained in school. The relationships among substance use, perceived harmfulness of substances, feelings of loneliness, trimester of pregnancy, age of the young women, and general health activities were examined" (p. 1). | Quantitative Cross-sectional | 432 urban, inner-city female students; 119 pregnant and 313 non-pregnant. |
| Proctor, S. E. (1996). *Loneliness and childbearing in adolescence* (Publication Number 9634290) [Doctoral dissertation.] University of California, San Francisco. ProQuest Dissertations & Theses Global. | Loneliness and Childbearing in Adolescence | US | "This study examined the association between loneliness, parents, and childbearing in adolescence among a sample of first-time birthing adolescents. The study assessed loneliness, as the primary construct, and the relationship of loneliness to perceived parental expressiveness, both maternal and paternal, quality of communication with parents, both mother and father, and perceived family strength, all, before and after the birth of the infant. A subsample of the original group was interviewed to further explore the significance of the infant to the self, to measures of loneliness, and to perception of parental relationships and family” (p. 3). | Mixed Methods | 84 pregnant adolescent females . |
| Ritchie, J. (1980). Social characteristics of a sample of solo mothers. *New Zealand Medical Journal, 91*(659), 349-352. | Social Characteristics of a Sample of Solo Mothers | New Zealand | “This study yielded data which enable us to compare the child rearing patterns of solo parents with those of parents living in the dual parent situation” (p. 349). | Quantitative Cross-sectional | 158 solo mothers of four-year-old children. |
| Robbins, J. M., & DeLamater, J. D. (1985). Support from significant others and loneliness following induced abortion. *Social Psychiatry, 20*(2), 92-99. | Support From Significant Others and Loneliness Following Induced Abortion | US | “The relationship between support from significant others and feelings of loneliness 1 week after induced abortion was investigated” (p. 92). | Quantitative Cross-sectional Descriptive | 228 abortion recipients. |
| Rokach, A. (2004). Giving life: Loneliness, pregnancy, and motherhood. *Social Behavior and Personality: An International Journal, 32*(7), 691-702. https://doi.org/10.2224/sbp.2004.32.7.691 | Giving Life: Loneliness, Pregnancy, and Motherhood | Canada | This study aimed at exploring the sources of new mothers' alienation, and understanding what contributes to their loneliness so that pregnant women and new mothers may reduce and cope better with their loneliness. | Quantitative Descriptive | 396 women - 91 pregnant, 97 in the first year of motherhood, and 208 who were neither. |
| Rokach, A. (2005). Coping with loneliness during pregnancy and motherhood. *Psychology and Education: An Interdisciplinary Journal, 42*(1), 1-12. | Coping with Loneliness During Pregnancy and Motherhood | Canada | “The present study examined the manner in which pregnant and new mothers cope with loneliness” (p. 4). | Quantitative Descriptive | 397 women - 91 pregnant, 97 in their first year of motherhood, and 208 who were neither. |
| Rokach, A. (2007). Self-perception of the antecedents of loneliness among new mothers and pregnant women. *Psychological Reports, 100*(1), 231-243. | Self-perception of the Antecedents of Loneliness Among New Mothers and Pregnant Women | Canada | This study aimed at exploring the sources of new mothers' alienation, and understanding what contributes to their loneliness so that pregnant women and new mothers may reduce and cope better with their loneliness. | Quantitative Descriptive | 396 women - 91 pregnant, 97 in the first year of motherhood, and 208 who were neither. |
| Rolls, C., & Hanna, B. (2001). What about the mother and family when an infant doesn't sleep? *Australian Journal of Primary Health, 7*(3), 49-53. | What About the Mother and Family when an Infant Doesn't Sleep? | Australia | "The aims of the research were to gain an understanding from the women's perspective of their experiences of having a child with a sleep problem and to assess whether a five-day residential stay in an EPC is of benefit to families" (p. 50). | Qualitative Focus Group | 28 women, two men and one grandmother. All participants had a child (aged between 6 weeks and 18 months) with a sleep problem that was causing distress in the family. (p. 50). |
| Russo, A., Lewis, B., Joyce, A., Crockett, B., & Luchters, S. (2015). A qualitative exploration of the emotional wellbeing and support needs of new mothers from Afghanistan living in Melbourne, Australia. *BMC Pregnancy and Childbirth, 15*, 197. https://doi.org/10.1186/s12884-015-0631-z | A Qualitative Exploration of the Emotional Wellbeing and Support Needs of New Mothers from Afghanistan Living in Melbourne, Australia | Australia | "This research aimed to explored the experiences of Afghan women living in Melbourne throughout pregnancy, birth and early motherhood, and gain insight into the aspects of their experiences that they perceive as positively and negatively impacting their emotional wellbeing" (abstract). | Qualitative Descriptive | 38 Afghanistan-born women, aged at least 18, who had migrated to Australia, had a child less than 5 years old, and had given birth in Australia. |
| Sable, M. R., Washington, C. C., Schwartz, L. R., & Jorgenson, M. (2007). Social well-being in pregnant women: Intended versus unintended pregnancies. *Journal of Psychosocial Nursing and Mental Health Services, 45*(12), 24-31. | Social Well-Being in Pregnant Women: Intended Versus Unintended Pregnancies | US | To examine the relationship between pregnancy intention (unintended versus intended pregnancy) and social well-being. "The goal of this study was to examine whether perceived loneliness, lack of social support, or family relationship problems were associated with pregnancy intention or feelings toward pregnancy” (p. 29). | Quantitative Descriptive | 72 pregnant women aged 18 to 35 who were, mostly low-income and attending Women, Infants, and Children programs and prenatal clinics in a midwestern community. |
| Samano, R., Martinez-Rojano, H., Robichaux, D., Rodriguez-Ventura, A. L., Sanchez-Jimenez, B., de la Luz Hoyuela, M., Godinez, E., & Segovia, S. (2017). Family context and individual situation of teens before, during and after pregnancy in Mexico City. *BMC Pregnancy and Childbirth, 17*(1), 382. https://doi.org/10.1186/s12884-017-1570-7 | Family Context and Individual Situation of Teens Before, During and After Pregnancy in Mexico City | Mexico | "The present qualitative study explores the social reality of pregnant teens in the Mexico City metropolitan area." "The aim of the study was to explore factors in the individual and family context of teenage girls that can be present with teen pregnancy” (p. 3). | Qualitative Ethnography | 29 teen mothers who were low- to lower-middle class in Mexico City, as well as 6 mothers, four fathers, and four partners of the pregnant women interviewed. |
| Santos, H. P., Jr., Kossakowski, J. J., Schwartz, T. A., Beeber, L., & Fried, E. I. (2018). Longitudinal network structure of depression symptoms and self-efficacy in low-income mothers. *PloS One, 13*(1), e0191675. https://doi.org/10.1371/journal.pone.0191675 | Longitudinal Network Structure of Depression Symptoms and Self-Efficacy in Low-income Mothers | Amsterdam and US | "The goal of this paper is thus to explore the specific interactions between self-efficacy and individual maternal depression symptoms, and test whether these are consistent over time" (p. 2). "This is the first study to explore the multivariate structural dependencies among depression symptoms and self-efficacy in a sample of low-income mothers, and to explore the temporal stability of network structures over four time points" (p. 8). | Quantitative Correlational Network Analysis | 306 low-income women aged at least 15 years old and mothers of infants or toddlers aged 6 weeks to 36 months who scored at least 16 on the CES-D depression scale, not reciting psychotherapy or counseling or on psychotropic medication. |
| Saunders, T., & Lawrence, J. (2018). Coming full circle: Building a sustainable community of mothers. *Practising Midwife, 21*(3), 35-38. | Coming Full Circle: Building a Sustainable Community of Mothers | UK | “The UN sustainable development goals aim to promote a more sustainable planet through addressing key areas of sustainability; key to these are goals related to health and wellbeing. As health care professionals, midwives are an integral part of this, as they develop close and long-lasting relationships with women and their families. This is vital in the ever-changing landscape of health care provision, where the demise of the extended family has resulted in increased numbers of women feeling isolated and lonely during pregnancy, which affects mental wellbeing both short-and long term" (p. 35). | Editorial | N/A |
| Schuez-Havupalo, L., Lahti, E., Junttila, N., Toivonen, L., Aromaa, M., Rautava, P., Peltola, V., & Raiha, H. (2018). Parents' depression and loneliness during pregnancy and respiratory infections in the offspring: A prospective birth cohort study. *PloS One, 13*(9), e0203650. https://doi.org/10.1371/journal.pone.0203650 | Parents' Depression and Loneliness During Pregnancy and Respiratory Infections in the Offspring: A Prospective Birth Cohort Study | Finland | “In this study our aim was to evaluate whether mothers' and fathers' depressive symptoms and loneliness during pregnancy predict higher rates of respiratory tract infections in the offspring." (abstract). "Within the context of a large Finnish cohort study (the Steps study), we aimed to examine, whether mothers' and fathers' antenatal depressive symptoms and loneliness are associated with higher rates of acute otitis media (AOM), RTI-related physician visits and antibiotic treatments in infants, and whether they have an influence on parental healthcare-seeking behavior. We hypothesized that parental depression and loneliness during pregnancy predict a higher burden of RTIs in the offspring" (p. 2). | Quantitative Correlation | 924 parents of children who were part of the birth cohort of the observational 'Steps to the Healthy Development and Wellbeing of Children' Study. |
| Shorey, S., Chee, C. Y. I., Ng, E. D., Lau, Y., Dennis, C. L., & Chan, Y. H. (2019). Evaluation of a technology-based peer-wupport intervention program for preventing postnatal depression (Part 1): Randomized controlled trial. *Journal of Medical Internet Research, 21*(8), e12410. https://doi.org/10.2196/12410 | Evaluation Of a Technology-based Peer-support Intervention Program for Preventing Postnatal Depression (Part 1): Randomized Controlled Trial | Singapore and Canada | "According to a recent review, an effective technology-based PND prevention intervention should be short term, be conducted immediately postpartum at an individual level, and target at-risk women instead of the general population. By incorporating all these elements, this study aimed to evaluate the effectiveness of a technology-based peer-support intervention program (PIP) on maternal outcomes during the early postpartum period (3 months postpartum)” (pp. 2-3). | Quantitative RCT | 138 mothers at risk of PND (69 intervention, 69 control). |
| Smith, J. E. (2007). *Prenatal maternal stress and coping among vulnerable rural young women* (Publication Number 3296690) [Doctoral dissertation.] University of South Carolina. ProQuest Dissertations & Theses Global. | Prenatal Maternal Stress and Coping Among Vulnerable Rural Young Women | US | “Purposes of this study are to examine the psychosocial variables (social support, optimism, depressive symptoms and loneliness), vulnerability variables (age, ethnicity, and socioeconomic level), stress appraisal variables (maternal prenatal stress and perceived global stress), and their relation to adolescent coping and health behaviors during pregnancy," (p. 7). | Quantitative Descriptive Correlation | 62 pregnant adolescent girls ranging from ages 16 to 19. |
| Sorenson, D. S. (2003). Healing traumatizing provider interactions among women through short-term group therapy. *Archives of Psychiatric Nursing, 17*(6), 259-269. | Healing Traumatizing Provider Interactions Among Women Through Short-term Group Therapy | US | "The purpose of this investigation was to test a short-term, cognitive group therapy intervention method to reduce or resolve ongoing psychological disruption and/or trauma among women who experienced traumatizing provider interactions (TPI) in their childbearing experience” (p. 259). | Causal-comparative/Quasi Experimental | 9 women 6-weeks postpartum with self-identified, significant psychological disruption after a traumatizing provider interaction during a birth experience unrelated to the baby's physical outcome. |
| Spinetta, J. J. (1978). Parental personality factors in child abuse. *Journal of Consulting and Clinical Psychology, 46*(6), 1409-1414. https://doi.org/10.1037//0022-006X.46.6.1409 | Parental Personality Factors in Child Abuse | US | The present study is an attempt to demonstrate that however one might explain the particular circumstances that helped shape the parents' personality, abusing parents differ from non-abusing parents in attitudinal and personality variables" (p. 1410). | Quantitative Descriptive | 7 adjudicated abusers, 9 spouses of abusers, 13 parents convicted of neglect, 15 non-abusing mothers from a college population, 15 mothers from a middle socioeconomic level, and 41 mothers from a lower socioeconomic level. |
| Stewart, M., Dennis, C. L., Kariwo, M., Kushner, K. E., Letourneau, N., Makumbe, K., Makwarimba, E., & Shizha, E. (2015). Challenges faced by refugee new parents from Africa in Canada. *Journal of Immigrant & Minority Health, 17*(4), 1146-1156. https://doi.org/10.1007/s10903-014-0062-3 | Challenges Faced by Refugee New Parents from Africa in Canada | Canada | “Consequently, the purpose of this study was to examine challenges faced by Sudanese and Zimbabwean refugee new parents in Canada. Three research questions guided this study. From the perspectives of Sudanese and Zimbabwean refugees in Canada: (1) What are their experiences of stress and coping concurrently with challenges related to migration and new parenthood? (2) What are their experiences with loneliness, social isolation, and mental health challenges? (3) What are the implications for supportive services, programs, and policies?" (p. 1147). | Mixed Methods | 72 "Participants were Sudanese and Zimbabwean refugee mothers and fathers who came to Canada in the previous 5 years and had a baby born in Canada. |
| Stewart, M., Kushner, K. E., Dennis, C., Kariwo, M., Letourneau, N., Makumbe, K., Makwarimba, E., & Shizha, E. (2017). Social support needs of Sudanese and Zimbabwean refugee new parents in Canada. *International Journal of Migration, Health & Social Care, 13*(2), 234-252. https://doi.org/10.1108/IJMHSC-07-2014-0028 | Social Support Needs of Sudanese and Zimbabwean Refugee New Parents in Canada | Canada | “The purpose of this paper is to examine support needs of African refugee new parents in Canada, and identify support preferences that may enhance the mental health of refugee parents and children” (abstract). | Mixed Methods | 72 refugee new parents (36 from Zimbabwe, 36 from Sudan) with a child aged 4 months to 5 years. |
| Stewart, M., Makwarimba, E., Letourneau, N. L., Kushner, K. E., Spitzer, D. L., Dennis, C. L., & Shizha, E. (2015). Impacts of a Support Intervention for Zimbabwean and Sudanese Refugee Parents: "I Am Not Alone". *Canadian Journal of Nursing Research, 47*(4), 113-140. https://doi.org/10.1177/084456211504700407 | Impacts of a Support Intervention for Zimbabwean and Sudanese Refugee Parents: "I am not Alone" | Canada | "The purpose of this pilot study was to design and evaluate the effects of an accessible and culturally appropriate social support intervention that meets the support needs and preferences identified by new parents who are refugees," (p. 117). "The study was guided by a four-part research question: What are the perceptions, values, and beliefs of new parents who are refugee about the impacts of the social support intervention on their (1) support resources (e.g., social, informational); (2) loneliness and isolation (discrepancies between ideal and perceived interpersonal relationships producing and maintaining feelings of loneliness and isolation [Cacioppo & Hawkley, 2009]); (3) coping (proactive coping using the resources of others - practical, informational, and emotional [Greenglass, 2002]); and (4) parenting stress (attributed to the behaviour of the child, to difficulty managing parenting tasks, or to dysfunctional interaction between child and parent [Abidin, 1995])?” (p. 117). | Mixed Methods | 85 new parents who were refugees (48 Sudanese and 37 Zimbabwean), had an infant at least 4 months old, and who had been living in Canada for less than 5 years. |
| Tuominen, M., Junttila, N., Ahonen, P., & Rautava, P. (2016). The effect of relational continuity of care in maternity and child health clinics on parenting self-efficacy of mothers and fathers with loneliness and depressive symptoms. *Scandinavian Journal of Psychology, 57*(3), 193-200. https://doi.org/10.1111/sjop.12284 | The Effect of Relational Continuity of Care in Maternity and Child Health Clinics on Parenting Self-efficacy Of Mothers and Fathers with Loneliness and Depressive Symptoms | Finland | The aim of the study was to examine how the relational continuity of care experienced in maternity health clinics (MHCs) and child health clinics (CHCs) affect mothers' and fathers' parenting self-efficacy (PSE) when the child is 18 months old. "Our research questions were: (1) Is there an association between the relational continuity of care in MHCs and CHCs and mothers’ and fathers’ PSE, loneliness and depressive symptoms?; and (2) Is the effect of loneliness and depressive symptoms on parents’ PSE different between the parents who have experienced relational continuity of care in MHCs and CHCs and those who have not?”, (p. 194). | Quantitative Cross-sectional | 1,797 women who participated in the STEPS follow-up study. |
| Van der Gucht, N., & Lewis, K. (2015). Women's experiences of coping with pain during childbirth: A critical review of qualitative research. *Midwifery, 31*(3), 349-358. https://doi.org/10.1016/j.midw.2014.12.005 | Women's Experiences of Coping with Pain During Childbirth: A Critical Review of Qualitative Research | UK | "…to identify and analyse qualitative literature exploring women's experiences of coping with pain during childbirth” (pp. 350-351). | Review | N/A |
| Vicary, J. R., & Corneal, D. A. (2001). A comparison of young women's psychosocial status based on age of their first childbirth. *Family and Community Health, 24*(2), 73-84. | A Comparison of Young Women's Psychosocial Status Based on Age of Their First Childbirth | US | “This 12-year longitudinal study examined the psychological status; social relationships; and home, work, and parenting stress and satisfaction in their young adulthood for a sample of rural women who were teen mothers compared to their cohort who had their first child in their twenties" (abstract). | Quantitative Correlational | 177 rural, low socioeconomic background females followed from junior high school through their mid-twenties. |
| Webber, G., & Wilson, R. (1993). Childbirth in the north- A qualitative study in the Moose Factory zone. *Canadian Family Physician, 39*, 781-788. | Childbirth In the North- A Qualitative Study in the Moose Factory Zone | Canada | “This study explores the views of Mushkegowuk Cree women in the Moose Factory zone to reveal the spectrum of concerns, the perceived positive factors of evacuation, the desired improvements, and preferences for birthing" (p. 781). "The purpose of this study was to document how Cree women in the Moose Factory zone perceived their experience of evacuation, and what changes they would choose to make in current policy" (p. 782). | Qualitative Descriptive | Childbirth in the north. A qualitative study in the Moose Factory zone. |
| Yang, Y. O., Peden-McAlpine, C., & Chen, C. H. (2007). A qualitative study of the experiences of Taiwanese women having their first baby after the age of 35 years. *Midwifery, 23*(4), 343-349. | A Qualitative Study of The Experiences of Taiwanese Women Having Their First Baby After the Age of 35 | US and Taiwan | “…to explore the experiences of Taiwanese women who become pregnant for the first time after the age of 35 years" (abstract). | Qualitative Phenomenology | 10 first-time pregnant women, in their third trimester and without pregnancy complications, aged 35-44. |
| Zaidi, F., Nigam, A., Anjum, R., & Agarwalla, R. (2017). Postpartum depression in women: A risk factor analysis. *Journal of Clinical and Diagnostic Research JCDR, 11*(8), QC13-QC16. https://doi.org/10.7860/JCDR/2017/25480.10479 | Postpartum Depression in Women: A Risk Factor Analysis | India | “To estimate the commonly associated risk factors of PPD among the women coming to a tertiary hospital in New Delhi, India” (abstract). | Quantitative Correlation | 149 postnatal women. |
