# Supplementary material for: Loneliness in pregnant and postpartum people and parents of children aged 5 years or younger: a scoping review
Source: Syst Rev. 2022 Sep 7;11:196. doi: 10.1186/s13643-022-02065-5 (PMC9451126; doi:10.1186/s13643-022-02065-5)
Supplement: Supplementary file 1 — Additional file 1: Table 1. Summary of Documents Included. Information about the records included in this review, as well as additional details including study aims, designs of included studies, and characteristics of the studies’ samples. Table 2. Data Extracted on Parental Loneliness contains data related to the research questions of this scoping review, including what type of loneliness was identified (if authors addressed loneliness type), study results, definition of loneliness used (if authors defined loneliness), means for measuring loneliness (if loneliness was measured), factors associated with and protective of loneliness, and prevalence of loneliness within the study sample. [file 13643_2022_2065_MOESM1_ESM.zip › Supplemental Table 2-Data Extracted on Parental Loneliness R4.docx]

**Supplemental Table 2: Extracted Data on Parental Loneliness in Included Documents**

| Author & Year | Loneliness Type Identified | Outcomes/  Results | Loneliness Definition | Loneliness Measurement | Factors Associated with Loneliness | Loneliness Protective Factors | Loneliness Prevalence |
| --- | --- | --- | --- | --- | --- | --- | --- |
| Aching, M. C., & Granato, T. M. M. (2016). The good enough mother under social vulnerability conditions. *Estudos de Psicologia, 33*(1), 15-24. https://doi.org/10.1590/1982-02752016000100003 | N/A | Common in the study’s narratives was the idea that childrearing offers the chance for women to develop as mothers, but also common were feelings of helplessness, abandonment, and loneliness. | N/A | N/A | Women who identified as having been abandoned and/or dealing with the logistics of being a single parent under vulnerable and precarious conditions. | N/A | N/A |
| Amphlett, J. E. (1998). *Less than kin and more than kind: Maternal playgroup experience* (Publication Number 9832055) [Doctoral dissertation.] Massachusetts School of Professional Psychology. ProQuest Dissertations & Theses Global. | N/A | For all the women in this study, playgroups succeeded in reducing the sense of individual burden and loneliness. | N/A | N/A | Being a new mom, and feeling the need for camaraderie and community. | Mother felt that the playgroup experience offered some protection from isolation. | N/A |
| Arimoto, A., & Tadaka, E. (2019). Reliability and validity of Japanese versions of the UCLA loneliness scale version 3 for use among mothers with infants and toddlers: A cross-sectional study. *BMC Women's Health, 19*(1), 105. https://doi.org/10.1186/s12905-019-0792-4 | N/A | “Scales showed goodness of fit/acceptable fit for all three UCLA scales tested when compared with the original scales. Additionally, the score on the UCLA-LS3-J was positively correlated with childcare burden and negatively correlated with social networks. "The loneliness of mothers would be influenced by difference in quality, namely of interpersonal relationships and exchanges” (p. 5). | Loneliness is the 'unpleasant experience that occurs when a person's network of social relations is deficient in some important way, either quantitatively or qualitatively,'" (p. 1). Attributed to: Perlman D, Peplau LA. Toward a social psychology of loneliness. In: Gilmour R, Duck S, editors. London: Academic Press; 1981. | Revised UCLA loneliness scale - Japanese Version | Childcare burden and deficient social networks. | N/A | N/A |
| Armstrong, M. A. (1992). *Being pregnant and using drugs: A retrospective phenomenological inquiry* (Publication Number 9223368) [Doctoral dissertation.] University of San Diego. ProQuest Dissertations & Theses Global. | N/A | Experiencing pregnancy included intentional pregnancy to create or extend a family, and accidental pregnancy, welcomed or unwelcomed. Experiences of self included extreme social and emotional isolation, mistrust of others, and difficulty managing emotional issues. Use of drugs incurred feelings of guilt and self-hatred while paradoxically assuaging loneliness, and distancing and dissipating negative feelings. Emotional isolation is discussed in detail beginning on page 124. | N/A | N/A | Drug lifestyles isolated these women from their non-drug using families and friends. | N/A | N/A |
| Aydin, R., Korukcu, O., & Kabukcuoglu, K. (2019). Investigation of the experiences of mothers living through prenatal loss incidents: A qualitative study. *Journal of Nursing Research, 27*(3), e22. https://doi.org/10.1097/jnr.0000000000000289 | N/A | "During this period, parents live through a sudden emptiness and feeling of loneliness" (p. 6). | N/A | N/A | Loneliness results from the physical loss of a pregnancy. See page four. | N/A | N/A |
| Ayers, S., Crawley, R., Webb, R., Button, S., Thornton, A., & group, H. A. c. (2019). What are women stressed about after birth? *Birth, 46*(4), 678-685. https://doi.org/10.1111/birt.12455 | N/A | “Our findings emphasize the importance of exploring stressors and psychological well-being with women to provide support, help women's adjustment postpartum, and ensure interventions are offered when appropriate” (p. 678). Within the category of Adjusting to Life with a Baby, Loneliness was reported as a postpartum stressor by four women (2.7%), p. 680-681. Three cross-cutting themes emerged including lack of support from others. | N/A | N/A | Shouldering the bulk of childcare responsibilities leading to alienation from friend network (p. 682). | N/A | N/A |
| Badaru, U. M., Ogwumike, O. O., Adeniyi, A. F., & Kaka, B. (2013). Psychosocial adversities and depression in mothers of children with cerebral palsy in Nigeria. *Journal of Pediatric Neurology, 11*(1), 1-7. | N/A | “The results show that psychosocial adversity items, which may be broadly classified into poor marital relationships, inadequate social interaction and adverse economic conditions were significantly associated with depression among mothers of children with CP in Nigeria," (p. 5). Psychosocial variable of loneliness was experienced by 38% of the overall n, and in 4% of non-depressed moms, and 77% of depressed moms, p. 4. "The significant positive correlation that exists between psychosocial adversity and depression indicates that the more the psychosocial stress experienced, the higher the risk of the mother developing depression. | N/A | Psychosocial Adversity Scale (PAS) - 12 item tool with items assessing feelings of  loneliness, lack of confidants, and isolation. | There was a greater frequency of loneliness experienced by depressed moms than non-depressed moms - depression was significantly associated with isolation, feelings of loneliness, and lack of confidants. | N/A | N/A |
| Bandyopadhyay, M., Small, R., Watson, L. F., & Brown, S. (2010). Life with a new baby: how do immigrant and Australian-born women's experiences compare? *Australian and New Zealand Journal of Public Health, 34*(4), 412-421. https://doi.org/10.1111/j.1753-6405.2010.00575.x | N/A | “However, compared with Australian-born women, immigrant mothers less proficient in English did have a higher prevalence of depression (28.8% vs 15%) and were more likely to report wanting more practical (65.2% vs 55.4%) and emotional (65.2% vs 44.1% support. They were more likely to have no 'time out' from baby care (47% vs 28% and to report feeling lonely and isolated (39% vs 17%)” (p. 412). | N/A | N/A | Thirty-nine percent of Immigrants who reported speaking English less than very well reported loneliness (compared to 17% of Australian-born women). | N/A | N/A |
| Beck, C. T. (1992). The lived experience of postpartum depression: A phenomenological study. *Nursing Research, 41*(3), 166-170. | N/A | Feelings of loneliness prevailed because mothers felt that no one really understood the nightmare they were going through. | N/A | N/A | Loneliness was identified in the experiences of women with postpartum depression. | N/A | N/A |
| Beck, C. T. (2002). Postpartum depression: A metasynthesis. *Qualitative Health Research*, *12*(4),  453-472. https://doi.org/10.1177/104973202129120016 | N/A | “In synthesizing the translations, it became apparent that these themes reflected four perspectives involved with postpartum depression: (a) incongruity between expectations and reality of motherhood, (b) spiraling downward, (c) pervasive loss, and (d) making gains," (p. 458). | N/A | N/A | “As mothers silenced themselves and withdrew socially, they felt a profound sense of isolation and loneliness. Postpartum-depressed women were enveloped in unbearable loneliness due to the discomfort they felt being around others and their belief that no one else really understood what they were experiencing" (p. 464). | Support groups. | N/A |
| Bloom, T., Glass, N., Curry, M. A., Hernandez, R., & Houck, G. (2013). Maternal stress exposures, reactions, and priorities for stress reduction among low-income, urban women. *Journal of Midwifery & Women's Health, 58*(2), 167-174. https://doi.org/10.1111/j.1542-2011.2012.00197.x | N/A | Three stressors were most commonly reported in the sample: financial stress, violence, and isolation/loneliness" (p. 170). | N/A | N/A | Loss of friend network when priorities changed in pregnancy and when they stopped drinking or using drugs (p. 171). | N/A | N/A |
| Bondas-Salonen, T. (1998). How women experience the presence of their partners at the births of their babies. *Qualitative Health Research, 8*(6), 784-800. | N/A | “The partners' presence could alleviate the suffering of loneliness, pain, and uncertainty during delivery and find the woman’s strength to endure the suffering as well as share their joy. Even when women were insulted by their partners, they still preferred their partners' presence. The presence meant communion to the women and, in its deepest sense, the creation of families” (p. 784). | N/A | N/A | N/A | "The men's presence and their caring could alleviate the women's feelings of loneliness, pain, fear, and anxiety” (p. 794). | N/A |
| Botha, E., Joronen, K., & Kaunonen, M. (2019). The consequences of having an excessively crying infant in the family: An integrative literature review. *Scandinavian Journal of Caring Sciences, 33*(4), 779-790. https://doi.org/10.1111/scs.12702 | N/A | Ten themes were identified: The consequences of having an excessively crying infant in the family create desperation. It ruins everyday life, impairs breastfeeding, isolates and casts parents into loneliness, strains and breaks family relationships with feelings of failure as a parent” (p. 779). | N/A | N/A | “Inconsolable crying isolates and casts the parents into loneliness. The excessively crying infant can bring the family into social exclusion, isolation and loneliness (7, 31, 36, 37). They might seek and fail to get support from people and professionals around them (30, 35, 37). The parents are dis- appointed and disillusioned with health professionals, expecting them to change in how they work (30, 37)” (p. 786). | N/A | N/A |
| Callan, V. J., & Hennessey, J. F. (1988). The psychological adjustment of women experiencing infertility. *British Journal of Medical Psychology, 61*(Pt 2), 137-140. | N/A | “While women reported similar feelings of general enjoyment, usefulness and love in their lives, infertile women rated life as less interesting, more lonely and they were less contented with life" (p. 138). | N/A | N/A | “The lower levels of personal happiness reported by infertile women seem linked to their perceptions of life as less interesting and more lonely” (p. 139). | N/A | N/A |
| Charter, R., Ussher, J. M., Perz, J., & Robinson, K. (2018). The transgender parent: Experiences and constructions of pregnancy and parenthood for transgender men in Australia. *International Journal of Transgenderism, 19*(1), 64-77. https://doi.org/10.1080/15532739.2017.1399496 | N/A | Exclusion, isolation, and loneliness were the predominant features of trans men's experiences of gestational pregnancies. Healthcare systems are not generally supportive of trans bodies and identities and trans men encounter significant issues when interacting with healthcare providers. “The Isolation of Exclusion” (p. 68). | N/A | N/A | “Many participants echoed this account and reported that "when you're growing up female" motherhood is positioned as "nonnegotiable" or "assumed" and that to diverge or question that narrative, as Stevie (46) notes, "makes you feel even less normal than you already do" (p. 72). | N/A | N/A |
| Chiaradonna, W. (1982). A group work approach to post-surrender treatment of unwed mothers. *Social Work with Groups: A Journal of Community and Clinical Practice, 5*(4), 47-68. https://doi.org/10.1300/J009v05n04_05 | N/A | N/A | N/A | N/A | The decision to not tell family and/or friends about the pregnancy resulted in them being cut off from this social support and experiencing loneliness (p. 58). | N/A | N/A |
| Childs, R. E. (1985). Maternal psychological conflicts associated with the birth of a retarded child. *Maternal-Child Nursing Journal, 14*(3), 175-182. | N/A | 13 categories of responses were developed and are listed in order of frequency of occurrence: guilt, denial, inferiority, question religious beliefs, shame, confusion, death wish, anger, need to blame others, loneliness, unloved, infanticide, and helplessness (pp. 176-179). | N/A | N/A | Loneliness. “Seventy percent of the mothers expressed feelings of loneliness during the events following the birth of their retarded child. The mothers expressed feelings of being on an island with no one else. They talked of sensing that what they were experiencing was unique to themselves. They felt desolate, solitary, and singled out. Many commented that the words, ‘I know how you feel’ were an insult” (p. 179). | N/A | 70% |
| Connelly, J. J., Golding, J., Gregory, S. P., Ring, S. M., Davis, J. M., Davey Smith, G., Harris, J. C., Carter, C. S., & Pembrey, M. (2014). Personality, behavior and environmental features associated with OXTR genetic variants in British mothers. *PLoS ONE* [Electronic Resource] 2014;9(3):e90465. DOI: [10.1371/journal.pone.0090465](https://dx.doi.org/10.1371/journal.pone.0090465) | N/A | "There were 22 tests and for only one was P,0.05 - i.e. no more than would have been expected by chance. This association with rs53576 indicated that the women with GG reported lower levels of emotional loneliness than those with the A allele” (p. 3). | N/A | Emotional loneliness measured using the perceived social support scale, p. 2. | N/A | N/A | N/A |
| Cote-Arsenault, D., & Denney-Koelsch, E. (2011). "My baby is a person”: Parents' experiences with life-threatening fetal diagnosis. *Journal of Palliative Medicine, 14*(12), 1302-1308. https://doi.org/10.1089/jpm.2011.0165 | N/A | The theme of "utterly alone," which crossed the study's two theme categories, describes "how the parents' sense of social isolation adds to their personal sense of loss and loneliness," (p. 1302). "...disconnect between parents' personal experiences and that of others, experienced through their interactions with others, leading to an intense sense of isolation that heightened the parents' experience of loss," (p. 1304). They wanted to be understood, hopefully by friends, certainly by family (p. 1307). | N/A | N/A | The parents desired to share their personal pregnancy experience with friends, family, and medical providers. When they didn't find the understanding and support they were seeking, feelings of utter loneliness set in (p. 1306). Parents felt that no one really understood what they were going through, which left them feeling "stuck on an island," (p. 1307). | N/A | N/A |
| Cronin, C. (2003). First-time mothers – identifying their needs, perceptions and experiences. *Journal of Clinical Nursing (Wiley-Blackwell), 12*(2), 260-267. https://doi.org/10.1046/j.1365-2702.2003.00684.x | N/A | Psychological issues that emerged as themes for the new mothers were depression, loneliness, frustration, and 'letting go' (p. 265). "Although it appears that there was substantial support for mothers in terms of infant care, a sense of loneliness was reported" (p. 265). | N/A | N/A | N/A | N/A | N/A |
| Cutrona, C. E. (1981). *Depressive Attributional Style and Nonpsychotic Postpartum Depression* (Publication Number 8201082) [Doctoral dissertation.] University of California, Los Angeles. ProQuest Dissertations & Theses Global. | N/A | “Women who scored high on loneliness during pregnancy were more depressed than other women after the birth of their baby." "...deficiencies in relationships with family, friends, and guidance figures were closely linked to the level of postpartum depression." "Infant-related stress was also a major determinant of postpartum depression scores, especially in the first two weeks after delivery," (p. xiii). "Inadequate Social Support [a scale composed in part of the UCLA Loneliness Scale] remained a highly significant predictor of depression scores" (p. 163). | N/A | 20-item revised UCLA loneliness scale |  | N/A | N/A |
| Cutrona, C. E. (1986). Objective determinants of perceived social support. *Journal of Personality and Social Psychology, 50*(2), 349-355. | N/A | “Because all of the new mothers were married or living with someone, frequency of contact did not reflect the presence or absence of a committed partner. The strength of the relation between marital status and perceived social support is a reminder of the potential importance of a single key relationship with another person" (p. 353). | N/A | 20-item revised UCLA loneliness scale | N/A | N/A | N/A |
| Dennis, Hodnett, E., Kenton, L., Weston, J., Zupancic, J., Stewart, D. E., & Kiss, A. (2009). Effect of peer support on prevention of postnatal depression among high risk women: Multisite randomised controlled trial. BMJ, 338(7689), 230–284. https://doi.org/10.1136/bmj.a3064 | N/A | Telephone based peer support can be effective in preventing postnatal depression among women at high risk," (p. 1). "Women in the intervention group were significantly less likely to have symptoms of postnatal depression at the 12 week assessment than those in the control group," (p. 5), (14% of the intervention group compared to 25% of the control group). The intervention group had lower levels of anxiety at 12 weeks (p. 7). | N/A | UCLA Loneliness Scale- shortened version (version 3) | N/A | N/A | N/A |
| DiIorio, C., & Riley, B. (1988). Predictors of loneliness in pregnant teenagers. *Public Health Nursing, 5*(2), 110-115. | N/A | "Data analysis revealed a significant negative correlation between self-concept and loneliness and a significant negative correlation between future time perspective and loneliness. Stepwise multiple regression analysis indicated that self-concept and future time perspective together explained 38 percent of the variance in loneliness. The findings revealed that self-concept was the best predictor of loneliness in this sample and that the prediction of loneliness was strengthened in the presence of a weak future time perspective” (p. 110). | N/A | 20-item revised UCLA loneliness scale | Diminished self-concept is a strong predictor of loneliness among this population, p. 11. Future-time perspective was inversely related to loneliness (i.e. lack of educational goals). In other words, poor self-concept and constricted future-time perspective are predictors of loneliness in this population. | Those who wanted their pregnancy displayed significantly higher future time perspective scores and total self-concept scores (p. 114). | N/A |
| Eissler, L. A. (2002). *The experience of medically indicated relocation during high-risk pregnancy: A phenomenological study* (Publication Number 1409947) [Master's thesis.] University of Alaska Anchorage. ProQuest Dissertations & Theses Global. | N/A | “Findings of the study included concerns regarding the well-being of the baby, loneliness and isolation, and difficulties of relocation despite its recognized necessity. Lack of control over life circumstances was a prominent theme. Participants discussed coping methods: communication, time-consuming activities, and acknowledging positive aspects of their circumstances." “You’re Here and They’re There” was the theme that captures participants’ feelings about family, home, and loneliness,” (p. 22). | N/A | N/A | High-risk pregnancy necessitated relocation of these mothers to acute in-patient facilities, resulting in loneliness. | Receiving phone calls from loved ones and compassionate caregivers. | N/A |
| Ellis, S. A., Wojnar, D. M., & Pettinato, M. (2015). Conception, pregnancy, and birth experiences of male and gender variant gestational parents: It's how we could have a family. *Journal of Midwifery & Women's Health, 60*(1), 62-69. https://doi.org/10.1111/jmwh.12213 | N/A | “Loneliness was the overarching theme that permeated participants’ experiences, social interactions, and emotional responses during every stage of achieving biologic parenthood. Within the context of loneliness, participants described complex internal and external processes of navigating identity. The struggles associated with navigating identify included undergoing internal struggles and engaging with the external world,” (p. 62). “Persistent loneliness and navigating identity formed a constant and significant backdrop of daily life for all participants, requiring a considerable investment of energy and attention” (p. 63). | N/A | N/A | “Associated with loneliness were the internal and external struggles of navigating identity. "Following the decision to pursue gestational parenthood, and prior to achieving a successful pregnancy, participants entered a time of ambiguity that can aptly be described as entering the unknown. Most participants experienced this as a particularly lonely and overwhelming time,” (p. 65). | N/A | 100% |
| Engnes, K., Liden, E., & Lundgren, I. (2012). Experiences of being exposed to intimate partner violence during pregnancy. *International Journal of Qualitative Studies on Health and Well being, 7*. https://doi.org/10.3402/qhw.v7i0.11199 | N/A | Feeling lonely" was one of five "constituents" that emerged from the two main themes of existential choices and ambivalence | N/A | N/A | The women reported that their partners wanted to be with them all of the time and did not want the women to spend time with friends. Additionally, women reported feelings of shame associated with exposure to violence that creates distance to friends. “…It means that when things are difficult I have nobody,” (p. 6). Women shared that friends became tired of hearing about their experiences of violence. | N/A | N/A |
| Fords, G. M., Crowley, T., & van der Merwe, A. S. (2017). The lived experiences of rural women diagnosed with the human immunodeficiency virus in the antenatal period. *SAHARA J: Journal of Social Aspects of HIV/AIDS Research Alliance, 14*(1), 85-92. https://doi.org/10.1080/17290376.2017.1379430 | N/A | A loneliness that hurts" was one of four themes that emerged. "Participants described their lives after an HIV diagnosis as filled with loneliness - a loneliness that hurt as it was not possible for them that any other person could comprehend the haunting thoughts, the feelings of isolation and the fear from loved ones," (p. 88). | N/A | N/A | "The women, throughout their pregnancy and, for many, after their delivery of their babies, experienced blame, fear, the cruelties of stigma, stereotyping and judging, and ultimately avoided any closeness or romantic relationships. Many expressed drastic changes in their socialising patterns and that they spent more time alone” (p. 88). | Others were able to cope as they relied on their spiritual beliefs," (p. 88). "Many found solace and acceptance in the privacy of their own homes and avoided society due to the cruelties of stigma" p. 89). Some reported the support of their mothers. | N/A |
| Fry, M. J., Cartwright, D. W., Huang, R. C., & Davies, M. W. (2003). Preterm birth a long distance from home and its significant social and financial stress. *Australian and New Zealand Journal of Obstetrics and Gynaecology, 43*(4), 317-321. | N/A | All mothers described isolation, loneliness, poor social support and significant financial hardship related to getting their infants back to a local hospital or home. | N/A | N/A | Missing the support of their partner, family and/or friends while temporarily relocated at the hospital after emergency delivery of a preterm baby. | N/A | N/A |
| Garthus-Niegel, S., Storksen, H. T., Torgersen, L., Von Soest, T., & Eberhard-Gran, M. (2011). The Wijma Delivery Expectancy/Experience Questionnaire: A factor analytic study. *Journal of Psychosomatic Obstetrics & Gynecology, 32*(3), 160-163. https://doi.org/10.3109/0167482X.2011.573110 | N/A | Loneliness was one of six factors identified in fear of childbirth (FOC). | N/A | The Wijma Delivery Expectancy/Experience Questionnaire (measures fear of  childbirth; loneliness is one of six factors measuring the domains of fear of childbirth) | Fear of childbirth. | N/A | N/A |
| Geller, J. S. (2004). Loneliness and pregnancy in an urban Latino community: Associations with maternal age and unscheduled hospital utilization. *Journal of Psychosomatic Obstetrics & Gynecology, 25*(3-4), 203-209. | N/A | Increased loneliness is associated with increased unscheduled pregnancy related hospital utilization during pregnancy. Older pregnant women had higher loneliness scores. Loneliness was more significant than age in predicting higher unscheduled hospital visits. "In other words younger pregnant mothers were much less lonely than the normal population, and older pregnant women were much more lonely than the control group" (p. 208). "This finding clearly has many implications in terms of the potential direct monetary cost of loneliness to society as a whole as a result of higher health resource utilization" p. 207. "The combination of higher loneliness and younger age predicts the pregnant women most likely to come unexpectedly for a hospital visit" p. 208. | Loneliness is the perception of insufficient social, physical, and emotional support when needed. | UCLA Loneliness Scale (version 3) | Lonely mothers were twice as likely to be high hospital users, p. 207. Older mothers were more likely to be lonely than their younger counterparts. | N/A | N/A |
| Goedecke, D. M., & Jones, E. (1991). *A comparison of personal factors in pregnant and non-pregnant adolescent girls* (Publication Number 1346408) [Master's thesis.] The University of Arizona. ProQuest Dissertations & Theses Global | The author believes that there has been a relative neglect to exploring the intrapersonal and interpersonal factors which may be related to weakened defenses that increase the likelihood of unwanted adolescent pregnancy, (p. 17). In this study, loneliness is viewed as an intra-personal mediating factor to a stressor (potential sexual activity) which may or may not result in the decision to engage in sexual activity. (p. 12-14). Other mediating factors were: interpersonal (social support from friends and family) or intra-personal (factors of loneliness and sexual-self concept). | “The mean loneliness score for the pregnant subjects was 53.30 while the mean score for the non-pregnant subjects was 52.14 thus demonstrating no significant differences between the two groups” (p. 73). | “Loneliness is an unpleasant experience and has been operationalized as the psychological state resulting from dissatisfaction with the number and quality of one's social and emotional relationships (Goswick and Jones, 1981)” (p. 34). | 20-item revised UCLA loneliness scale | "Since loneliness is a symptom of identity diffusion (Erikson, 1968), it is important to examine the relationships of self-concept and future orientation to loneliness in adolescents (DiIorio & Riley, 1988)," (p. 34). "Loneliness has been found to be consistently associated with problematic emotions, self-derogation and less positive attitudes towards others, as well as a variety of social and personal problems," (p. 34). Thinking poorly of oneself. Expectation that others will reject the lonely person. Failure to use interpersonal opportunities to alleviate loneliness. Feeling dissatisfied by the quality of relationships. "Their findings also indicated that greater loneliness was associated with tendencies to negatively evaluate one's body, sexuality, health, and appearance" (p. 35). | N/A | N/A |
| Harms, V. O., & Abbott, D. A. (1994). *The relationship of family functioning and self-perception to adolescent pregnancy: A cultural perspective* (Publication Number 9425285) [Doctoral dissertation.] The University of Nebraska - Lincoln. ProQuest Dissertations & Theses Global. | N/A | The two individual variables of esteem and loneliness were able to discriminate between the groups at significant levels. The pregnant group reported lower levels of individual loneliness and higher levels of individual esteem. | The amount of social or emotional isolation an individual experiences. | Loneliness Inventory-Short Form (10-item, 5-point Likert Scale) | Hypothesis of risk for becoming pregnant (results found that the pregnant group was less lonely than the non-pregnant group at a significant level). The author's possible explanations for the results of higher esteem and lower loneliness in the pregnant group were 1) the pregnant adolescents may have attempted to meet their needs by being involved in a sexual relationship, or 2) popular girls, being less lonely and having higher esteem, may be at higher risk for pregnancy. | N/A | N/A |
| Heaman, M., & Gupton, A. (1998). Perceptions of bed rest by women with high-risk pregnancies: A comparison between home and hospital. *Birth, 25*(4), 252-258. | N/A | Bed rest had a significant emotional and social impact on pregnant women and their families in both settings. Overall, bed rest in hospital seemed to be associated with more sources of stress than at home. In hospital, women had to cope with separation from home and family, lack of privacy, hospital discomforts, and incompatible roommates, whereas women at home struggled with role reversal and the temptation to do more activity than was recommended. | N/A | N/A | Women on bed rest in the hospital stated that the worst part of being in the hospital was being away from their homes and families. Feelings of depression and loneliness were predominant among women in the hospital. Women expressed unmet expectations for social interactions with their friends and family, but then guilt was also experienced when husbands would visit because the husband was assuming all family responsibilities. | N/A | N/A |
| Hudson, D. B., Campbell-Grossman, C., Kupzyk, K. A., Brown, S. E., Yates, B. C., & Hanna, K. M. (2016). Social support and psychosocial well-being among low-income, adolescent, African American, first-time mothers. *Clinical Nurse Specialist, 30*(3), 150-158. https://doi.org/10.1097/NUR.0000000000000202 | N/A | "At every time point, positive correlations were found between emotional support and self-esteem and between problematic support and loneliness." | “Loneliness, an exceedingly unpleasant and distressing experience resulting from perceived deficiencies in a person's relationships, is an important aspect of well-being for adolescents in general” (p. 5). | 20-item Revised UCLA Loneliness Scale | Problematic social support. | N/A | N/A |
| Hudson, D. B., Elek, S. M., & Campbell-Grossman, C. (2000). Depression, self-esteem, loneliness, and social support among adolescent mothers participating in the New Parents Project. *Adolescence, 35*(139), 445-453. | N/A | There was a negative relationship between depression and social support (r = -.61, p < .05). Social support was positively associated with self-esteem (r = .65, p < .05) and negatively associated with loneliness (r = -.50, p < .05). Loneliness was correlated with depression (r = .53, p < .05) and inversely correlated with self-esteem (r = -.74, p < .001)" (p. 450). | N/A | 20-item Revised UCLA Loneliness Scale | Loneliness was correlated with depression and inversely correlated with self-esteem. | N/A | N/A |
| Huttlinger, K. W. (1988). *The experience of pregnancy in teenage girls* (Publication Number 8822424) [Doctoral dissertation.] University of Arizona. ProQuest Dissertations & Theses Global. | N/A | Loneliness was one of 8 themes that were revealed. | N/A | N/A | Being pregnant made the teenagers feel separate from their friends and family. Not having someone to talk to was another common reason for feeling lonely during pregnancy. They felt like their friends had moved on without them. Others felt different and alone. Some felt alone because they had been abandoned by the fathers of their babies. They also shared that the feeling of alienation was related to their loneliness. | N/A | N/A |
| Igarashi, Y., Horiuchi, S., & Porter, S. E. (2013). Immigrants' experiences of maternity care in Japan. *Journal of Community Health, 38*(4), 781-790. https://doi.org/10.1007/s10900-013-9679-8 | N/A | When immigrant women had some Japanese literacy, obstruction to communication was more likely to occur presumably because some literacy unreasonably increased health care providers' expectations of a higher level of communication. This was associated with loneliness, and when loneliness was strongly felt, care satisfaction was lower. | N/A | Revised UCLA loneliness scale - Japanese version | Healthcare provider over estimation of literacy level is thought to cause confusion and feelings of isolation and loneliness (p. 788). | N/A | N/A |
| Jabraeili, M., Hassankhani, H., Negarandeh, R., Abbaszadeh, M., & Cleveland, L. M. (2018). Mothers' emotional experiences providing care for their infants within the culture of an Iranian neonatal unit. *Advances in Neonatal Care, 18*(4), E3-E12. https://doi.org/10.1097/ANC.0000000000000530 | N/A | Loneliness was one for four themes that emerged. "Loneliness consisted of bearing the burden of care while feeling alone” p. E3). This theme consisted of two sub-themes: 1) burden of care, and 2) feeling alone, (p. E7). | N/A | N/A | Mothers were responsible for infant care in the NU 24 hours per day without respite, help from family and without formation of partnerships between mothers and nurses. Nurses often blamed the mothers if something went wrong. This contributed to mothers feeling alone, unsupported and unsure of their mothering skills and/or their ability to care for their infant. (p. E7) | N/A | N/A |
| Jopling, K., & Sserwanja, I. (2016). *Loneliness Across the Life Course: A Rapid Review of the Evidence*. Calouste Gulbenkian Foundation, UK Branch. https://gulbenkian.pt/uk-branch/ | N/A | N/A | "The Campaign defines loneliness as a subjective, unwelcome feeling of lack or loss of companionship, which happens when we have a mismatch between the quantity and quality of social relationships that we have, and those that we want. This definition draws on the work of Perlman and Peplau2 which is commonly referred to across the literature, and attracts a good degree of consensus,” (p. 3). | N/A | N/A | N/A | N/A |
| Jundt, K., Haertl, K., Knobbe, A., Kaestner, R., Friese, K., & Peschers, U. M. (2009). Pregnant women after physical and sexual abuse in Germany. *Gynecologic and Obstetric Investigation, 68*(2), 82-87. https://doi.org/10.1159/000215931 | N/A | "The Hospital Anxiety Depression Scale (HADS) to evaluate symptoms of anxiety and depression and the SCL-K-9 demonstrated significantly more feelings of depression and anxiety, strain, loneliness, vulnerability and less expectation of happiness for their future in abused women" (p. 84). | N/A | Hospital Anxiety Depression Scale (HADS-D/A and HADS-D/D) and the SCL-K-9 | Physical and sexual abuse may complicate a woman's experience of pregnancy, leading to increased feelings of depression, anxiety, strain, loneliness and less expectation of happiness for their future (p. 82). | N/A | N/A |
| Junttila, N., Ahlqvist-Bjorkroth, S., Aromaa, M., Rautava, P., Piha, J., & Raiha, H. (2015). Intercorrelations and developmental pathways of mothers' and fathers' loneliness during pregnancy, infancy and toddlerhood--STEPS study. *Scandinavian Journal of Psychology, 56*(5), 482-488. https://doi.org/10.1111/sjop.12241 | Social and emotional loneliness | Both mothers’ social and emotional loneliness and fathers’ social and emotional loneliness were highly stable, and within individuals these loneliness factors were strongly correlated. However, the correlations between mothers’ loneliness experiences and fathers’ loneliness experiences were weaker than expected. Separate latent growth curve groups were identified, which differed in feelings of marital dissatisfaction, social phobia, and depression. These groupings revealed that the higher the loneliness was, the more parents experienced these other psychosocial problems. Social Loneliness of mothers: 78.2% were stable non-lonely, 16.8% were stable lonely, and 5% increasingly lonely. Social Loneliness of fathers: 62.8% decreasing non-lonely, 30.5% increasing average lonely, and 6.5% increasing lonely. Emotional Loneliness in mothers: 85.5% stable non-lonely, 5.8% stable lonely, 8.7% increasingly lonely. Emotional Loneliness in fathers: 59.4% decreasing non-lonely, 32.6% stable average lonely, and 8% stable lonely. Overall, marital dissatisfaction, social phobia, and depressive symptoms were higher in the average lonely and lonely groups than in the non-lonely groups. | “Loneliness is defined as the subjective feelings of being without the type of relationships that are desired — a discrepancy between one’s real and desired relationships,” (p. 482). “The commonly accepted definition of social loneliness is the absence of a social network or the feeling that one is not part of a group. Emotional loneliness, in turn, refers to the lack of a close, intimate attachment to another person” (p. 482). | 20-item Revised UCLA Loneliness Scale | Findings supported the idea that a significant relationship does not protect individuals from the feelings of loneliness, p. 486. “However, the lonelier the parents, the more problems they had in their couple relationship, social functioning, and mental well-being,” (p. 486). “In conclusion, becoming a parent may increase both mothers’ as well as fathers’ feelings of social and emotional loneliness and these phenomena are highly associated with lower levels of marital satisfactions and higher levels of social phobia and depression,” (p. 487). “This combination of risk factors may make the mothers especially vulnerable for increasing social and emotional loneliness during the early phase of motherhood,” (p. 486). “…we also know that a strong predictor for both mothers’ and fathers’ poor parental self-efficacy is her/his own feelings of social and emotional loneliness,” (p. 487) Additionally, an important mediator between parent’s mental health and a child’s positive outcome seems to be parenting self-efficacy. | N/A | N/A |
| Junttila, N., Ahlqvist-Björkroth, S., Aromaa, M., Rautava, P., Piha, J., Vauras, M., Lagström, H., & Räihä, H. (2013). Mothers' and fathers' loneliness during pregnancy, infancy and toddlerhood. *Psychology and Education: An Interdisciplinary Journal, 50*(3-4), 98-104. http://search.ebscohost.com/login.aspx?direct=true&db=psyh&AN=2013-35445-010&site=ehost-live | Social and emotional loneliness | “After a few minor modifications were made, the Finnish shortened version of the UCLA Loneliness Scale showed good construct validity for evaluation of mothers' and fathers' social and emotional loneliness." " Based on the longitudinal CFA results, the stability of mothers' and fathers' social and emotional loneliness was high from pregnancy until their child was 18 months old. To our knowledge, no research on the stability of mothers' and fathers' social and emotional loneliness during this time point in life exists. Interestingly, this study showed that men were lonelier than women during pregnancy. However, after the birth of the child mothers had greater levels of loneliness" (p. 102). | "Loneliness is a painful and stressful experience signaling the existence of a personal failure in the valued area of interpersonal relationships." "The commonly accepted definition of social loneliness is that it refers to the absence of a social network or to the feeling that one is not part of a group." Emotional loneliness, in turn, refers to the lack of a close, intimate attachment to another person" (p. 98). | Finnish shortened version of the UCLA Loneliness Scale | N/A | N/A | N/A |
| Kane, A. H. (1964). Loneliness in young mothers. *Nursing Mirror, 118*, 489-489. | N/A | N/A | N/A | N/A | Perhaps some people may consider a 'Friendship Bureau' to be hardly part of a health visitor's work, but no one can deny that promoting the mental health of lonely mothers by encouraging them to discuss the difficulties of motherhood with others similarly situated, and thus help to relieve their anxieties, is one of the health visitor's main duties" (p. 490). | N/A | N/A |
| Kantar Public, Co-operative Group (Great Britain), & British Red Cross Society. (2016). *Trapped in a bubble: An investigation into triggers for loneliness in the UK*. Kantar Public=. https://www.redcross.org.uk/about-us/what-we-do/action-on-loneliness | N/A | “Young new mums – Participants noted that even where their initial social connections were strong, either before or shortly after the birth of their baby, they had experienced diminishing support from friends and family as the months went on. For those with a strong existing support network, the influx of attention and support was followed by increasingly fewer encounters, as the ‘novelty’ of the new baby wore off. The high cost of childcare reinforced the identity of the young mum as a mum because they could not afford a childminder while they went out. And, as noted above, some perceived judgement from older mums and others in their community about the choices they had made to have children ‘early’.” | “loneliness is best described as the distressing feeling that individuals experience when a person’s network of social relationships with others is less satisfying than they desire in either  quality or quantity” (p. 12). | The British Red Cross and Co-op Loneliness Quantitative Survey  Questionnaire V10 | “ … the research confirmed that people experiencing life events which can disrupt existing connections were at risk” (for loneliness) (p. 10). “This research has evidenced life transitions – and particularly role transition – as disruptive moments that increase the risk of loneliness amongst individuals. When existing social connections are challenged or severed – for example through a break-up of a relationship, emergence of a serious health issue, or retirement – this can reduce opportunities for ‘easy’ connection and also threaten self-identity. A range of barriers to connection – which vary from person to person and can occur across individual, community and social levels – can then additionally weaken people’s ability to make or sustain connections. Once habits of disconnection have set in, they become hard to break. Loneliness itself can become a barrier to connection. People who are experiencing loneliness can begin to view connection as a vulnerable and anxiety-ridden experience, or even begin to question their own self-worth” (p. 20). Describing alienation: ““It’s still looked down upon young mums most of the time I feel judged and made feel like a less than mum just because of my age often patronised by other older mums. This makes it hard to make friends as people my age have different priorities therefore there is not a lot in common.” (Young new mum, Belfast, 18-24), (p. 27). ““I took him to a play group once and all the mums were a lot older than me and quite cliquey so I have never been back.” (Young new mum, London, 18-24) (p. 28). | “Peer-led support was identified as necessary, encouraging individuals to connect with others who had been in similar circumstances but had managed to overcome them” (p. 9). “Trusted community advisors – Experts and participants saw trusted people in communities, such as GPs, housing associations and local authorities, as being well-placed to signpost to social support and activities, helping individuals to make the first step to accessing services and support” (p. 10). | 32% of parents of young children were always or often lonely. |
| Khan, S., Ion, A., Alyass, A., Greene, S., Kwaramba, G., Smith, S., Carvalhal, A., Kennedy, V. L., Walmsley, S., & Loutfy, M. f. t. H. I. V. M. S. T. (2019). Loneliness and perceived social support in pregnancy and early postpartum of mothers living with HIV in Ontario, Canada. *AIDS Care, 31*(3), 318-325. https://doi.org/10.1080/09540121.2018.1515469 | N/A | Significant associations between marital status, income, racism, and depressive symptoms were associated with perceived social support and loneliness. Marital status was found to have a positive effect, but this change did not persist with time. Income was also significantly associated with loneliness, but this did not persist over time. EDS Racism, EPDS scores were also significantly associated, and this association persisted with the change over time (p. 322). | N/A | UCLA Loneliness Scale | Marital status, income, racism, and depression. "Based on a review of the limited literature, as well as the practice experience of the lead researchers, loneliness and perceived social support were hypothesized to be important psychosocial issues that would contribute to the mental health and well-being of mothers living with HIV" (p. 319). “Understanding the relevant psychosocial variables that can increase the risk of loneliness and perceived social support is important to help target these issues,” (among the greatest risk factors of postpartum depression are loneliness, low perceived social support and impaired bonding.” | N/A | N/A |
| Kjelsvik, M., Sekse, R. J. T., Moi, A. L., Aasen, E. M., Chesla, C. A., & Gjengedal, E. (2018). Women's experiences when unsure about whether or not to have an abortion in the first trimester. *Health Care for Women International, 39*(7), 784-807. https://doi.org/10.1080/07399332.2018.146594 | N/A | They experienced the decision-making process as a lonely journey toward a definite conclusion during which their values were challenged" (p. 790). "A feeling of existential loneliness dominated the decision-making process and the implementation." | N/A | N/A | "Disagreement with the partner or others heightened the women's feeling of loneliness," (p. 793). The women described a tension between the need to openly discuss the situation and not being swayed by the opinions of others. "Health personnel who did not engage with them contributed to their sense of isolation” (p. 793). | Women expressed the need to talk with close family or friends whom they trusted and would not disclose their condition (p. 793). | N/A |
| Klein, T. M. (1998). Adolescent pregnancy and loneliness. *Public Health Nursing, 15*(5), 338-347. | This study investigated the relationships of characterological and situational loneliness. | Results of the ANOVA and Tukey-HSD indicated situational variables were more significant than characterological variables in understanding loneliness in early and middle adolescence. | "Weiss described characterological loneliness as that which stems from certain personality characteristics of an individual that interfere with the establishment or maintenance of relationships, while situational loneliness depends on relationally inadequate social circumstances in which the individual finds himself or herself” (p. 338). | 20-item revised UCLA Loneliness Scale | Shyness, self-esteem, perceived maternal expressiveness, perceived paternal expressiveness, and social support were all significantly related to loneliness. | "The findings suggest parental relationships were powerful in influencing the existence of loneliness as well as self-esteem, shyness, and social support." | N/A |
| Knight, A., Chase, E., & Aggleton, P. (2006). 'Someone of your own love': Experiences of being looked after as influences on teenage pregnancy. *Children and Society, 20*(5), 391-403. https://doi.org/10.1111/j.1099-0860.2006.00014.x | N/A | The young people reported feelings of loneliness, rejection, stigma and not being able to trust others, emotions which seriously influenced their decisions about becoming parents. These findings suggest that such young people may benefit from a greater degree of emotional and practical support throughout their lives in care." A theme that emerged from the interviews was Loneliness, Rejection and Trust, and threads of loneliness were evident in other themes. | N/A | N/A | Early pregnancy is related to the following. Participants said that they felt ostracized from and rejected by their families before going into care, resulting in loneliness and effects on their self-esteem. Participants felt unwanted by their families. Loneliness was also rooted in a sense of upheaval from frequently being replaced with new foster families. | N/A | N/A |
| Korukcu, O., Bulut, O., & Kukulu, K. (2016). Psychometric Evaluation of the Wijma Delivery Expectancy/Experience Questionnaire Version B. *Health Care for Women International, 37*(5), 550-567. https://doi.org/10.1080/07399332.2014.943838 | N/A | N/A | N/A | Wijma Delivery Expectancy/Experience Questionnaire Version B | N/A | N/A | N/A |
| Kroupa, S. E., & Carman, R. S. (1990). *The interpersonal world of the pregnant adolescent: A multiple comparison group approach* (Publication Number 9030609) [Doctoral dissertation.] University of Wyoming. | N/A | The pregnant teenage group, and two delinquent groups had higher average scores on the loneliness scale indicating greater interpersonal isolation as compared to "normal" adolescents and pregnant adults (p. 121). | N/A | 20-item Revised UCLA Loneliness Scale | N/A | N/A | N/A |
| Kruse, J. A., Williams, R. A., & Seng, J. S. (2014). Considering a Relational Model for Depression in Women with Postpartum Depression. *International Journal of Childbirth, 4*(3), 151-168. | N/A | The model explained 35% of the variance in postpartum depression with impaired bonding and loneliness as the strongest indicators. | Loneliness is the 'unpleasant experience that occurs when a person's network of social relations is deficient in some important way, either quantitatively or qualitatively' and occurs as an emotional response to a 'discrepancy between desired and achieved levels of social contact'" (p. 5). | National Women’s Study PTSD Module NWS-PTSD was used to assess loneliness in a  single item — “In the past month have you felt cut off from other people?” | Loneliness in the postpartum period had a direct effect on PPD as well as on impaired bonding. Note that this study found that impaired bonding indirectly affected PPD through loneliness - this is the opposite direction from most theories which consider impaired bonding as an outcome of maternal depression. | N/A | N/A |
| LeDrew, H. M., Moores, P., Read, T., & O'Regan-Hogan, M. (2018). He's here and he's gone; he's here and he's gone ... The experiences of new mothers in rural Newfoundland and Labrador, Canada, whose partners work away from home. *Rural & Remote Health, 18*(3), 4542. https://doi.org/10.22605/RRH4542 | N/A | While many women felt overwhelmed with the responsibilities of balancing family life, and often experienced loneliness, others described a smoother adaptation to an ERM lifestyle. The experiences of the mothers warrant consideration by leaders to be more responsive to the emerging needs of families in these situations. | N/A | N/A | Loneliness was an aspect of being in a 'one-woman' show (due to husbands' employment-related mobility). This loneliness was experienced in relation to the absence of a partner to share child care as well as in the need for companionship and socialization. Seeking help from others and living in a community where many families were in a similar situation reduced the sense of loneliness and solo responsibility. | N/A | N/A |
| Lee, K., Vasileiou, K., & Barnett, J. (2019). 'Lonely within the mother': An exploratory study of first-time mothers' experiences of loneliness. *Journal of Health Psychology, 24*(10), 1334-1344. https://doi.org/10.1177/1359105317723451 | N/A | All participants experienced a passing but sometimes acute loneliness in the transition of motherhood. Feelings of loneliness were related to their dissatisfaction with the quantity and quality of their relationships and interactions and to experiencing increased difficulty and vulnerability exacerbated by perceived deviations from 'norms' of motherhood. Feelings of loneliness derived from partners' perceived lack of empathy were discussed at length. | Young (1982) differentiated between three types of loneliness: transient, occasional and passing lonely moods; situational, triggered by a developmental or unexpected disruption; and chronic, lacking adequate social relations for two or more years" (p. 1334). | N/A | All mothers experienced the transition into motherhood as a challenging reality out of step with expectation. It was unanticipatedly difficult and increased vulnerability gave rise to feelings of loneliness. They felt prepared for childbirth but not for what followed. Breastfeeding alone at night was lonely because the mothers worried by themselves. Bottle feeding also produced feelings of loneliness as the mothers felt that they were failing to meet the prevalent cultural narrative of breast is best. This left them feeling vulnerable and inadequate, and fearing judgement and rejection of others. Mothers compared themselves to the cultural narrative of 'effortless motherhood' and felt isolated in an experience not aligned to the norm. They felt vulnerable to others' judgement for failing to meet this ideal. This vulnerability acted as a barrier to relating to others. Participants felt stuck/trapped in the house due to birth-related changes in normal routines resulting in less control over being able to see people outside of the house. When socializing, they felt separated from the group when they would excuse themselves to breastfeed (but could still hear the conversation continuing without them in the other room). Mothers related their loneliness to not having relationships with a depth of understanding of what they were going through. Mothers had expected parenthood to be a joint responsibility and were surprised to find that they were nearly solely responsible for caring for the infant. Support offered by partners was often perceived as lacking empathy/understanding of the mothers' experiences. pp. 1337 - 1340. Two participants were concerned about perceived hostility and judgement from other mothers, which amplified their feelings of loneliness (these mothers did not breastfeed) p.1341. | For some mothers, relationships with other mothers alleviated their loneliness and gave them 'reassurance of worth,' via a sense of camaraderie born from shared understanding. This might take the form of a middle of the night WhatsApp message when both up caring for their infants at night, or face-to-face discussions about the difficulties associated with having a young baby, which helped them to normalize their own experiences. p. 1340. | N/A |
| Lee, L. C., Yin, T. J., & Yu, S. (2009). Prenatal examination utilization and its determinants for immigrant women in Taiwan: an exploratory study. *Journal of Nursing Research, 17*(1), 73-82. https://doi.org/10.1097/JNR.0b013e3181999ee8 | N/A | Around one third of the women felt lonely (33.7%). Bivariate analysis indicated that the three variables of adaptation to life in Taiwan, perceived importance of having examinations, and feelings of loneliness were related to prenatal examination utilization. Following the schedule recommended by the NHI was also identified as a factor of influence. Participants most likely to follow NHI-scheduled prenatal examinations were those with better life adaptation, who perceived the importance of prenatal examinations, and who expressed no feelings of loneliness. (p. 76-78). | N/A | The Author's own questionnaire developed based on literature review findings, expert opinions, and interviews with pregnant Vietnamese women. Loneliness was measured as an independent variable as a need factor. See page 74. | “…negative emotions (defined as feelings of loneliness in this study) were found to negatively affect the utilization of medical services," (p. 79). Adaptation to life in Taiwan, feelings of loneliness, and perceived importance of prenatal examinations showed significant relationships with prenatal examination utilization. Adaptation to life in Taiwan and perceived importance of prenatal examinations showed positive relationships with such utilization, whereas feelings of loneliness showed a negative relationship” (p. 80). | N/A | N/A |
| Liu, L. L., Slap, G. B., Kinsman, S. B., & Khalid, N. (1994). Pregnancy among American Indian adolescents: reactions and prenatal care. *Journal of Adolescent Health, 15*(4), 336-341. | N/A | Seven of the 20 adolescents interviewed reported loneliness during pregnancy. | N/A | Modification of Kinsman and Slap's questionnaire. | Suicidal youth were more likely to hope for miscarriage, fear disclosure, express loneliness (83% suicidal vs 14% non-suicidal), and view prenatal care as unimportant. | N/A | N/A |
| Lundgren, I., & Berg, M. (2007). Central concepts in the midwife-woman relationship. *Scandinavian Journal of Caring Sciences, 21*(2), 220-228. | N/A | Six pairs of concepts were elucidated; each one describing one aspect from the woman's perspective and one responsive aspect from the midwife. One of these concept pairs was loneliness-confirmation. | N/A | N/A | In pregnancy, loneliness was experienced while coming to terms with the responsibility of having a child. In labor, loneliness was related to the midwife's physical or mental absence. | The midwife offered confirmation as an "anchored companion" during childbirth who helped the women to follow the childbearing process and also to be responsible for their birth through the support of the midwife. | N/A |
| Lundqvist, P., Weis, J., & Sivberg, B. (2019). Parents' journey caring for a preterm infant until discharge from hospital-based neonatal home care-A challenging process to cope with. *Journal of Clinical Nursing, 28*(15-16), 2966-2978. https://doi.org/10.1111/jocn.14891 | N/A | The parents' experiences differed. Mothers experienced more physiological reactions that triggered feelings of existential loneliness and guilt and difficulties in combining the role of mother with partner. | N/A | N/A | “The mothers felt very lonely this new situation despite support from their partner and the professionals” (p. 2972). | N/A | N/A |
| Luoma, I., Korhonen, M., Puura, K., & Salmelin, R. K. (2019). Maternal loneliness: Concurrent and longitudinal associations with depressive symptoms and child adjustment. *Psychology Health & Medicine, 24*(6), 667-679. https://doi.org/10.1080/13548506.2018.1554251 | N/A | “…feeling lonely at least sometimes was common among first time mothers: at each of the three time points, one third of the mothers reported feelings of loneliness” (p. 675). “As concluded in a review of earlier studies (Heinrich & Gullone, 2006), depression and loneliness seem to be overlapping but distinct phenomena” (p. 675). “The hypothesis that maternal loneliness predicts internalizing problems children was supported by the findings, but this association was statistically significant only from a longitudinal perspective” (p. 675). | "...whereas the subjective and qualitative evaluations of social relationships are emphasized in loneliness” (p. 668). | “Mothers’ feelings of loneliness were screened with the item, ‘Do you feel lonely?’ The response options were (1) ‘always,’ (2) ‘often,’ (3) ‘sometimes,’ (4) ‘rarely,’ (5) ‘never,’ and (6) ‘cannot say’ (option six was regarded as missing data). For analysis purposes, a dichotomized variable was created for each time point. The ‘lonely’ category includes options 1-3, whereas ‘not lonely’ includes options 4 and 5,” (p. 669). | “Loneliness during pregnancy was associated with the mothers’ physical health problems. Being a single mother at T2 predicated maternal loneliness at T3. At T2 and T3, loneliness showed concurrent associations with maternal poorer life satisfaction and dissatisfaction with the pair relationship, at T3 even with poorer life satisfaction at T2, and with poorer satisfaction with the pair relationship at T1 and T2. A high level of maternal prenatal depressive symptoms predicted loneliness at the subsequent stages but loneliness was associated with the presence of concurrent depressive symptoms only at T3” (p. 672). | N/A | “The dichotomized prevalence of loneliness (lonely sometimes, often, or always) was 38% during pregnancy and 34% at the subsequent stages,” (p. 672). |
| Lutz, W. J., & Hock, E. (2002). Parental emotions following the birth of the first child: gender differences in depressive symptoms. *American Journal of Orthopsychiatry, 72*(3), 415-421. | N/A | Our data reveal the importance of loneliness, which we hypothesized to be a salient contributor to men's depressive symptoms in this time of transition. In the initial regression utilizing both men and women, a significant Gender X Loneliness interaction was found, indicating that the effect of loneliness on depressive symptoms was greater in men than women. In addition, the beta weight for loneliness in the final model was significant only for men, indicating that fear of loneliness is more salient in the explanation of depressive symptoms in men. Although more studies are needed, our findings suggest that men whose personalities reflect high fear of loneliness may be particularly at risk for depressive symptoms after the birth of the first child because of their wives' increased focus on the infant" (p. 419). | N/A | Loneliness questions were formed using six items from the Depressive Experiences Questionnaire (DEQ) | For men and women, "personality characteristics (fear of abandonment and fear of loneliness) were significantly related to depressive symptoms." | N/A | N/A |
| Mandai, M., Kaso, M., Takahashi, Y., & Nakayama, T. (2018). Loneliness among mothers raising children under the age of 3 years and predictors with special reference to the use of SNS: a community-based cross-sectional study. *BMC Women's Health, 18*(1), 131. https://doi.org/10.1186/s12905-018-0625-x | N/A | "The present study found that low support from SNS friends significantly correlated with high levels of loneliness among mothers raising children, even after adjusting for economic instability, low support from family and friends, low IWMS-S score, and psychological distress” (p. 6). | “Peplau and Perlman defined loneliness as the ‘unpleasant experience that occurs when a person’s network of social relations is deficient in some important way, either quantitatively or qualitatively’” (p. 2). | Revised 20-item UCLA Loneliness Scale | "The degrees of loneliness in mothers raising children was associated with a smaller social network, lower secure attachment style, and a higher possibility of psychological distress." "Even after applying Bonferroni's correction (p < 0.0015), health status (p < 0.001) and subjective economic status (p < 0.001) were significantly associated with loneliness score. Loneliness Scale scores were highest among teenagers, followed by participants in their 40s and 20s, with the lowest scores observed among those in their 30s. Among married women, those with spouses who did not help with childrearing and housework had higher levels of loneliness. With respect to health status, loneliness tended to increase as health status worsened. For subjective economic status, the lowest loneliness scores were observed in those who re- ported that they were economically “somewhat stable,” followed by those who reported that they were “stable” and “somewhat unstable.” The highest level of loneliness was reported among those who were economically “unstable.” For education level, graduates of college/graduate school had the lowest Loneliness Scale scores, followed by graduates of trade school/junior college and graduates of junior high, with high school graduates reporting the highest degree of loneliness,” (p. 4). | "Mothers who spent an average of 1-2 h (daily) on the phone within the last 3 months showed the lowest level of loneliness. On the other hand, mothers who spent more than 2 h or fewer than 0.5 h on phones showed higher levels of loneliness" (p. 5). “Most participants had contacts with “parents” and “friends” as in- formation sources. For these sources, a higher frequency of consultation was correlated with lower levels of loneliness. Relative to those who did not use SNS as an information source, those who did tended to have lower levels of loneliness,” (p. 5).  “We found that loneliness tended to be lower as personal networks created through SNSs as well as traditional net- works of family and friends grew,” (p. 6). | N/A |
| Martin, B. P. (1995). *An analysis of common postpartum problems and adaptation strategies used by women during the first two to eight weeks following delivery of a fullterm healthy newborn* (Publication Number 9536488) [Doctoral dissertation.] University of Mississippi. ProQuest Dissertations & Theses Global. | N/A | The common postpartum problems identified in frequency of descending order were loss of sleep, being tired, feeling tense, increased household chores, feeling tied down, loss of income, concerns for personal appearance, inability to concentrate, feeling lonely, trouble coping with mothering role, and changes in sexual feelings. | N/A | Author's own questionnaire | N/A | N/A | 42% |
| Matos-Rios, A. Y. (1995). *Loneliness and intimacy of friendship among pregnant and nonpregnant adolescents* (Publication Number 9542142) [Doctoral dissertation.] Louisiana State University. ProQuest Dissertations & Theses Global. | N/A | Both pregnant and non-pregnant adolescents experienced loneliness, but no non-pregnant adolescents reported severe loneliness, whereas 4 of the pregnant participants did. | “Loneliness: Conceptual definition — the disruption or absence of ties with attachment figures, including relational deficits. The response to the absence of some particular provision. It is an enduring condition of emotional distress that arises when a person feels misunderstood or rejected by others and/or lacks appropriate social partners for desired activities, particularly activities that provide a sense of social integration and opportunities for emotional intimacy (Weiss, 1973)” (p. 5). “Operational definition — Loneliness is the score achieved on the UCLA Loneliness scale” (p. 5). | 20-item Revised UCLA Loneliness Scale | Questionnaire items significant in distinguishing feelings of loneliness between the two groups mean was higher for pregnant teenagers) were: feeling alone, not feeling close to people, and feeling isolated (p .80). Both groups considered their mother to be an important person in their lives, but the non-pregnant group considered their mother their best friend (p. 81). Non-pregnant participants were good at initiating relationships and considered female friends more meaningful than subjects in the pregnant group. Male friends were perceived more positively by pregnant teenagers than non-pregnant teenagers (p. 82). | N/A | N/A |
| Mauthner, N. S., Stoppard, J. M., & McMullen, L. M. (2003). 'Imprisoned in my own prison': A relational understanding of Sonya's story of postpartum depression. *Situating sadness: Women and depression in social context.*, 88-112. | N/A | N/A | N/A | "Her sense of paralysis, of being locked into a lonely and isolated world, was a recurring theme. Depression, she said, was like being "imprisoned in my own prison." Even though she wanted to confide her feelings, her sense of shame and fear of moral condemnation prevented her from reaching out" (p. 89). "A recurring theme within Sonya's interview was her desire to talk to people about her feelings of depression and her knowledge that this would help her recovery. However, she felt the individuals around her were not prepared to listen. When she tried talking to Johnie at the end of the working day his response was: “I don’t want to hear all this.” Gradually, Sonya withdrew and found it increasingly difficult to confide in people, even in those whom she suspected might have been supportive and sympathetic. For example, although she wanted to confide in her best friend Clare, her fear of rejection prevented her from doing so” (p. 100). “She feared that by being herself she would lose the friendship and respect of others. Paradoxically then, Sonya concealed her thoughts and feelings to gain approval and maintain a semblance of relationships with other people. Yet this also left her feeling alone and depressed” (p. 101). | "While the psychotherapy was helping her, Sonya wanted to go to a postpartum depression support group. She felt she had reached a point where she needed to hear other women's stories, and share her feelings with a wider group of women: ‘The ultimate reason for going is not that we’re all sitting there in tears but it’s like any group where everybody’s got something in common—you feel that there’s no holds barred. You can say “I did this. Isn’t it terrible?” and somebody else will say “But I did *that*,” you know. And it reaffirms the fact that you’re not isolated and you’re not alone and that’s the main point of why I want to go and at this point I feel strong enough to talk’” (p. 103). | N/A | N/A |
| Milner, J. S., & Wimberley, R. C. (1980). Prediction and explanation of child abuse. *Journal of Clinical Psychology, 36*(4), 875-884. | N/A | Through statistical analysis, a 7-factor solution gave rise to essential dimensions of child abuse: Distress, rigidity, child with problems. Problems from family and others, unhappiness, loneliness, negative concept of child and self. | N/A | Child Abuse Potential Inventory | "These experiences are interspersed with reports of sometimes feeling worthless and not being understood by others,” (p. 881). | N/A | N/A |
| Mommersteeg, P. M., Drost, J. T., Ottervanger, J. P., & Maas, A. H. (2016). Long-term follow-up of psychosocial distress after early onset preeclampsia: the Preeclampsia Risk EValuation in FEMales cohort study. *Journal of Psychosomatic Obstetrics & Gynecology, 37*(3), 101-109. https://doi.org/10.3109/0167482X.2016.1168396 | N/A | Having had a stillborn child or early neonatal death during the index pregnancy was associated with higher depressive symptoms, anxiety, fatigue, and loneliness in the PE group, but these factors explained only a small proportion of the variance in these psychosocial distress factors. | N/A | UCLA-Revised short (UCLA-R-S) version | There was an interaction term with the preeclampsia group with hypertension associated with loneliness which remained through regression. | N/A | N/A |
| Monti, F., & Mori, G. F. (2015). The 'times' of maternality. *From pregnancy to motherhood: Psychoanalytic aspects of the beginning of the mother-child relationship.*, 107-119. | N/A | N/A | N/A | N/A | The woman continuously sends out signals through her verbal and non-verbal "complaints" (feelings of loneliness, self-reproaching, anxiety, sleep disorders, somatic disorders such as rashes and backache). | N/A | N/A |
| Mossman, S. L. (1980). How to cure the home-alone blues... a new mother offers suggestions for coping with loneliness. *American Baby, 42*, 38-38. | N/A | N/A | N/A | N/A | Author describes a discrepancy between anticipated and experienced expectations of what it would be like to be at home with a baby after the birth. Because she had not anticipated the frustration, isolation, and even boredom, she recommends that first-time pregnant women spend some time reckoning with the realities of motherhood. "It's a good idea to think about how your life will change if you plan to stay home with your baby." She explains that the transition may be more difficult when family lives far away and friends have been workplace-based. | One of the best friends to have when you are a new mother is another new mother, or someone with a toddler who remembers what it is like to have a new baby." The author recommends that women be proactive during pregnancy to identify potential mother-friend relationships. She suggests work friends who have recently had children, neighbors, or meeting women at childbirth classes. After the baby's birth, she recommends being open to meeting other mothers while outside of the house. | N/A |
| Mugweni, L. (2009). *Exploring prenatal health promotion experiences of recent immigrant women* (Publication Number MR63954) [Master's thesis.] University of Manitoba (Canada). ProQuest Dissertations & Theses Global. | N/A | "Content revealed the following themes; social isolation and the need for support, lack of knowledge about prenatal service available, values and beliefs about pregnancy, language and cultural barriers. Social isolation was identified as a major contributor to immigrant women's feelings of loneliness, helplessness and depression. | N/A | N/A | Social isolation was identified as a major contributor to immigrant women's feelings of loneliness, helplessness and depression. Pregnancy complications, language barriers, being a single mother, and depression were identified as contributors to feelings of loneliness. | Family and friends were mentioned as important in the life of immigrant women during pregnancy. Women specifically mentioned the significance of female relatives such as mothers, sisters and aunts. Faith/spirituality was used to help them "through the difficult time." Social support helped them to adjust to pregnancy and deal with pregnancy complications. | N/A |
| Muller, M. E. (1989). *The development and testing of the Mueller Prenatal Attachment Inventory* (Publication Number 8926411) [Doctoral dissertation.] University of California, San Francisco,. ProQuest Dissertations & Theses Global. | N/A | MPAI scores had a low, but significant negative correlation to loneliness. | "Weiss (1984) pointed out that there are two types of loneliness. One form is experienced by the adult in response to the absence of attachment figures. The other type, feelings of exclusion, occurs when one does not have a place in an accepting community." | 20-item Revised UCLA Loneliness Scale | N/A | N/A | N/A |
| Nadelson, C. C. (1975). The pregnant teenager: Problems of choice in a developmental framework. *Psychiatric Opinion, 12*(2), 6-12. | N/A | N/A (opinion article) | N/A | N/A | N/A | N/A | N/A |
| Nahas, V. L., Hillege, S., & Amasheh, N. (1999). Postpartum depression. The lived experiences of Middle Eastern migrant women in Australia. *Journal of Nurse-Midwifery, 44*(1), 65-74. | N/A | Five themes emerged that illustrated the middle Eastern women's experiences of postpartum depression: 1) loneliness due to feelings of isolation and lack of social support, 2) helplessness due to inability to cope with the overwhelming task of fulfilling her traditional role as mother and wife, 3) fear of failure and being labeled a "bad mother" by in-laws, 4) insufficient knowledge about postpartum depression and available support services, and 5) coming to terms with postpartum depression by undertaking diversional activities and learning new skills. | N/A | N/A | "Most informants expressed a deep sense of loneliness due to isolation and lack of social support. They missed the support that they got from their own families, friends, and even neighbors. In Australia, they are left alone at home to look after the children while their husbands go to work. They explained that they do not have close neighbors whose ethnic backgrounds are similar to theirs and, therefore, missed the interaction with people who share their beliefs, values, and practices” (p. 69). | Arabic community centers where activities may relieve stress and loneliness. They can interact and talk with other moms about "life in general and postpartum depression in particular” (p. 70). | N/A |
| Nasir, R., Ahmad Zamani, Z., Khairudin, R., Wan Sulaiman, W. S., Mohd Sani, M. N., & Amin, A. S. (2016). Depression, loneliness and cognitive distortion among young unwed pregnant women in Malaysia: Counseling implications. *Asian Social Science*, *12*(8):104-109. DOI: [10.5539/ass.v12n8p104](https://dx.doi.org/10.5539/ass.v12n8p104) | N/A | Results of the study showed that there were positive significant correlations between depression and loneliness, depression and cognitive distortion and loneliness and cognitive distortion." "The results therefore indicate that the more depressed the unwed women are the more lonely they get and the higher their cognitive distortion" (p. 106). | N/A | 20-item Revised UCLA Loneliness Scale | In this study, the participants were placed in shelters for unwed mothers, away from their families and communities. Additionally, there is a negative social connotation about premarital sex in Muslim culture. These factors are not based on analysis from this study, but inferred from the author's article. | N/A | N/A |
| Nilsson, C., & Lundgren, I. (2009). Women's lived experience of fear of childbirth. *Midwifery, 25*(2), e1-9. | N/A | Four themes emerged from the essential structure of fear of childbirth, which was described as 'to lose oneself as a woman into loneliness'. The four themes were: a feeling of danger that threatens and appeals, feeling trapped, feeling like an inferior mother to be, and on your own (p. e4). | N/A | N/A | "The women made an effort to fulfil their own expectations and those of others. If this did not prove successful, the women's confidence was shaken, and feelings of failure turned inwards towards the woman herself as she began to feel weaker and inferior to other women. Feelings of guilt and shame increased the woman's vulnerability." "Loneliness meant that the woman was captured by the pregnancy and could not turn back; she was alone facing the birth and giving birth; nobody else could do it for her." “The existential loneliness the woman experienced scared her and shook her confidence in her ability to give birth. The woman also experienced fear of her own reactions during and after the birth, and during the transition to motherhood. The irrevocable fact of not having another alternative other than giving birth was terrifying. The feeling of not receiving support and understanding for the fear from those around increased the feeling of not being a real woman, which caused feelings of guilt and shame and an experience of weakness and vulnerability,” (p. e4). | N/A | N/A |
| Nims, C. L. (1997). *Postpartum depression: The lived experience* (Publication Number 1383715) [Master's thesis.] Medical College of Ohio. ProQuest Dissertations & Theses Global. | N/A | Feelings of isolation and loneliness was one of six themes that emerged from the interviews. | N/A | N/A | Emotional components of theme one (feelings of isolation and loneliness) included: rejection, distancing, confusion, and helplessness. Feelings of isolation and loneliness prohibited women from receiving nurturance and support from traditional support systems which would have enhanced their ability to cope. All four participants perceived that people close to them did not understand what they were experiencing. This led them to feel lonely and isolated, even though they perceived their support as helpful. “I shouldn’t say that men don’t understand it, but men — how can men understand it if we don’t understand it” (p. 39). | "While family was perceived as support, participation at a postpartum depression support group was seen as a relief from the nightmare. Attendance at the group was the first major step in recovery. The isolation and loneliness peeled away, the despair over the situation began to dissolve and hope that recovery was possible began to emerge" (p. 45). | N/A |
| Nystrom, K., & Ohrling, K. (2006). Parental support: Mothers' experience of electronic encounters. *Journal of Telemedicine and Telecare, 12*(4), 194-197. | N/A | The mothers felt that sharing experiences with others was supportive and that having new friends reduced their feeling of loneliness." "They stated that they appreciated the opportunity to talk about anything that bothered them and to be listened to ... Feelings that you can hardly stand become endurable when someone listens," (p. 196). "Becoming friends with others reduces the feeling of loneliness" was a sub-theme that emerged from interviews. | N/A | N/A | "Although all the mothers stated that they had a good social network, they had spent most days alone with their infants. To meet other mothers in the same situation made them feel less alone” (p. 196). | “Becoming friends with others reduced loneliness (p. 196). "In spite of difficult technology, they established such a relationship that they felt like real friends, which reduced the feeling of loneliness" (p. 196). | N/A |
| Olsson, P., Jansson, L., & Norberg, A. (1998). Parenthood as talked about in Swedish ante- and postnatal midwifery consultations. A qualitative study of 58 video-recorded consultations. *Scandinavian Journal of Caring Sciences, 12*(4), 205-214. https://www.ncbi.nlm.nih.gov/pubmed/10067646 | N/A | A phenomenological hermeneutic analysis of the meaning of being a mother revealed a complex and difficult situation of being both needed and dependent. The meaning of being a father revealed a struggle between distancing from and closeness to the child. The mate relationship was indicated as important and under strain. The metaphor of the ‘spiders web’, where the mother is the spider with the child mostly on her back, the father entering the web on her terms, summarizes the understanding. The results from this study could provide a basis for reflection on the status of the topic of parenthood, on the meaning of being a mother and a father disclosed in the consultations, and ultimately on the organization of ante- and postnatal midwifery care. | N/A | N/A | "Fearing isolation. Being a mother meant experiencing feelings of loneliness according to these conversations. Divergent views, experiences and feelings within the couple concerning sexuality, childcare, household work and employment revealed fears of isolation from the father of the child. The importance of a dialogue within the couple was indicated. The mother may fear isolation from the adult community while at home with the small children,” (p. 209). | N/A | N/A |
| Omer-Salim, A., Suri, S., Dadhich, J. P., Faridi, M. M., & Olsson, P. (2014). Theory and social practice of agency in combining breastfeeding and employment: A qualitative study among health workers in New Delhi, India. *Women & Birth: Journal of the Australian College of Midwives, 27*(4), 298-306. https://doi.org/10.1016/j.wombi.2014.07.002 | N/A | "This is a very lonely job" was one of the themes that emerged from the interviews. | N/A | N/A | This seems to reflect mothers' dissatisfaction with the ability of others to empathize with their experience: "There is a perceived lack of family support for the trusted care of the child upon return to work. Expectations from the family are for the mothers to continue doing most of the house work, and mothering work is not acknowledged by the family or the husband. There is not much faith in the extended family, particularly in the mother-in-law who, in turn, does not respect the mother's intentions and her efforts to make plans for the care of her child. Apprehension and loneliness in the situation and her endeavours exist. Upon reflection there may be disappointment in the way that the experience turned out to be.” (p. 303). | N/A | N/A |
| Ornelas, I. J., Perreira, K. M., Beeber, L., & Maxwell, L. (2009). Challenges and strategies to maintaining emotional health: qualitative perspectives of Mexican immigrant mothers. *Journal of Family Issues, 30*(11), 1556-1575. | N/A | "Most of the mothers in our study reported experiencing depressive symptoms after becoming parents. They expressed their symptoms as feelings of sadness, depression, loneliness, shame, and anxiety. economic stressors contributing to their emotional health included financial obligations, work, and child care. Social stressors included family separation, social isolation, and discrimination. To cope with these stressors, mothers relied heavily on social networks and community resources.” | N/A | N/A | “Before coming to the United States, many of the women had either lived with their parents or lived geographically close to them in Mexico. Therefore, their isolation in the United States presented a stark contrast to their socially embedded lives in Mexico. This isolation resulted in stress, anxiety, and loneliness among the women” (p. 1567). “These mothers noted that there were many times when they lacked the social support that they needed and that their isolation prevented them from finding it elsewhere in the community” (p. 1567). This statement reflects actual social isolation, as well as the perceived experience of social isolation/loneliness, as well as a lack of self-efficacy acting as a stressor: “In Mexico, when you have a baby, well your mother is always there, or your family is there. So, they help you to bathe him. They help you like to pick him up, to hold him, to change him. all of that. So, you come here and you find yourself alone and with a little baby that you don’t even know how to pick up” (p. 1568). | “Although not the majority, some women commented on how their husbands provided needed companionship and emotional support. As one woman stated, What makes it easier for me [is] having my husband’s help because, when he is not here and I am alone with my children, it is really difficult for me.” (pp. 1568-1569). See page 1569 for discussion of friends who have an understanding of the mothers’ experiences. “Almost all were aware of programs for new immigrant and low-income families, such as the EHS program, WIC, food banks, Medicaid, and free English classes. As one woman stated, ‘Here it is easier, because if you feel depressed, there are . . . groups which can help you. Or if you feel sad, you can talk with someone and they will help you. If you don’t have anything to feed them, you can go to, like, social services, like WIC, where they help you. I have more help than what I expected’” (p. 1571). | N/A |
| Palmer, L., Carlsson, G., Brunt, D., & Nystrom, M. (2015). Existential security is a necessary condition for continued breastfeeding despite severe initial difficulties: a lifeworld hermeneutical study. *International Breastfeeding Journal, 10*, 17. https://doi.org/10.1186/s13006-015-0042-9 | N/A | "Mothers who experience severe difficulties with initial breastfeeding feel both overtaken and violated not only by their own infants and their own bodies but also by their anger, expectations, loneliness and care from health professionals. These feelings of being overtaken and invaded provoke an existential crisis and place mothers at a turning point in which these feelings are compared and put in relation to one another in the negotiation of the decision to continue or cease breastfeeding. This decision thus depends on the possibility of feeling secure with the breastfeeding relationship. If insecurity dominates, this can, in severe cases, create a feeling of fear of breastfeeding that is so great that there is no alternative but to stop breastfeeding.” | N/A | N/A | "Breastfeeding difficulties can create feelings of loneliness that risk reinforcing feelings of uselessness and difference from other mothers." “The feeling of loneliness may lead to withdrawal because of a fear of being detected as underperforming, useless, and different. Such fear constitutes a barrier to continued breastfeeding. Instead, the mother searches for contact with others who have stopped breastfeeding and thus breastfeeding can be ceased. ‘I didn’t talk to [the nurses] about my problems or about how I felt but I kept face // I didn’t want to cry and collapse there and then, instead I went to them to weigh and measure him and then I went back home (ID04)’” (p. 6). | "In order to escape loneliness, safety is sought in others who can provide support for continued breastfeeding. The connection with others provides a sense of belonging that makes breastfeeding possible. ‘[My friend] had the same troubles and we talked a lot about that. And then I had some other friends who I talked to on the telephone, they have had troubles too and then I was very much at home alone during that time (ID05)’” (p. 6). | N/A |
| Perlman, D., & Milardo, R. M. (1988). Loneliness: A life-span, family perspective. *Families and social networks.*, 190-220. | N/A | N/A | Loneliness is the unpleasant experience that occurs when a person's network of social relationships is deficient in some important way, either qualitatively or quantitatively (p. 191). | N/A | the number of network members decreases, and marital satisfaction declines (p. 204). | N/A | N/A |
| Pletsch, P. K. (1984). *A Description And Comparison Of Health Related Activities Of Pregnant And Nonpregnant High School Students* (Publication Number 8422539) [Doctoral dissertation.] University of Illinois at Chicago. ProQuest Dissertations & Theses Global. | N/A | "Pregnant and non-pregnant subjects were similar in feelings of loneliness, perceptions of social support, and knowledge of risk of harm from substance use. Subjects reported feeling lonely but having someone to go to for help. | “Feelings of loneliness: self-report of one’s loneliness or lack of emotional support from other people.” | Adapted a questionnaire by Bachman and Johnston (1978) developed to assess the  lifestyles, values, and preferences of adolescents. Part D of the questionnaire measures loneliness  in a single item, “A lot of times I feel lonely,” with a five-option Likert response from Agree to  Disagree. | Loneliness was associated with sleep and risk of occasional use of alcohol or marijuana. Subjects who were more lonely were less likely to get adequate sleep and attributed less risk to people from occasional use of alcohol or marijuana" (p. 77). | N/A | N/A |
| Proctor, S. E. (1996). *Loneliness and childbearing in adolescence* (Publication Number 9634290) [Doctoral dissertation.] University of California, San Francisco. ProQuest Dissertations & Theses Global. | N/A | N/A | "Weiss postulates that loneliness of a particular kind, emotional isolation, or referred to in this writing as emotional loneliness, is an outcome of a primary attachment deficit/disorder across the life span. The attachment deficit is a result of inadequate/compromised attachment-seeking on the part of one individual, usually a child, and/or inadequate/compromised nurturance on the part of another individual, usually an adult and usually, the parent. Emotional loneliness is not manifested until adolescence.” See page 53-65 for discussion about definitions of loneliness, and comparison/contrast of theory. “Loneliness is the adolescent respondent’s perception of an unpleasant, distressing experience produced by: 1.) a relational deficit in the form of the absence of a close emotional attachment with an attachment figure or figures, or 2.) the absence of an accessible social network (synthesis of Weiss, 1973, pp. 18-19, 1982b p. 74, and Peplau & Perlman, 1982, p. 3). | 20-item Revised UCLA Loneliness Scale | "Loneliness was strongly tied to perceived relationships with parents. In a few cases, the unhappiness and loneliness resulting from the absence of parental nurturance was sufficiently intense as to propel subjects toward attempts at taking their own lives” (p. 283). | N/A | N/A |
| Ritchie, J. (1980). Social characteristics of a sample of solo mothers. *New Zealand Medical Journal, 91*(659), 349-352. | N/A | Loneliness and responsibility were cited as disadvantages of being a single parent. | N/A | Authors own questionnaire | "No one to talk to or share things with,” (p. 350). | 18 percent of respondents suggested a support group of other solo parents and sympathetic friends and relations. | N/A |
| Robbins, J. M., & DeLamater, J. D. (1985). Support from significant others and loneliness following induced abortion. *Social Psychiatry, 20*(2), 92-99. | N/A | Support from the male partner before, during, and after the procedure was shown to be related to less frequent feelings of loneliness among 228 abortion recipients. Involvement or support of parents before and during the procedure had no effect on loneliness. Women whose relationship with their mothers became closer after the abortion, however, were less likely to feel lonely. Results are discussed in terms of the sharing of responsibilities in pregnancy and abortion that is consistent with the meaning of social support. | N/A | Authors own questionnaire | "Support from the male partner before, during, and after the procedure was shown to be related to less frequent feelings of loneliness. Women who became more close with their mother after the procedure were less likely to feel lonely." "Not only did support from parents generally fail to protect the women from feelings of loneliness, the few women who talked to the fathers after the abortion were more likely to feel alone than those who did not" (p. 97). “Support from other friends and relatives also failed to alter the chances of post-abortion loneliness except in one instance. There was a tendency for the odds of loneliness to increase among women if they were accompanied to the clinic with someone other than parents or partner” (p. 97). | N/A | One-quarter of the women in our sample reported feeling lonely at least half of the time one week after the experience," (p. 97). |
| Rokach, A. (2004). Giving life: Loneliness, pregnancy, and motherhood. *Social Behavior and Personality: An International Journal, 32*(7), 691-702. https://doi.org/10.2224/sbp.2004.32.7.691 | N/A | The general population had consistently higher mean subscale scores than the other two samples who, contrary to expectation, did not score significantly differently. Rokach postulates that they may be due to the fact that the "sources and qualitative aspects of loneliness would be perceived in a similar way since they are occurring in a special and demanding period of a woman's life," (p. 240). | N/A | Self-developed 30-item Loneliness Questionnaire | N/A | N/A | N/A |
| Rokach, A. (2005). Coping with loneliness during pregnancy and motherhood. *Psychology and Education: An Interdisciplinary Journal, 42*(1), 1-12. | N/A | "Results indicated that the three groups cope differently with loneliness." "It may be suggested that, overall, pregnancy and motherhood may not have a significant effect on the manner that women cope with loneliness. A period of a maximum of 9 (pregnancy) + 12 (motherhood) months may not be able to significantly affect life-long strategies that those women used in order to deal with the pain of loneliness." | N/A | Self-developed 34-item Loneliness  Questionnaire | N/A | N/A | N/A |
| Rokach, A. (2007). Self-perception of the antecedents of loneliness among new mothers and pregnant women. *Psychological Reports, 100*(1), 231-243. | N/A | The general population had consistently higher mean subscale scores than the other two samples who, contrary to expectation, did not score significantly differently. Rokach postulates that they may be due to the fact that the "sources and qualitative aspects of loneliness would be perceived in a similar way since they are occurring in a special and demanding period of a woman's life," (p. 240). | N/A | 29-item Loneliness Antecedents Questionnaire | N/A | N/A | N/A |
| Rolls, C., & Hanna, B. (2001). What about the mother and family when an infant doesn't sleep? *Australian Journal of Primary Health, 7*(3), 49-53. | N/A | The women felt alone, isolated, tired and overwhelmed by the endless demands of an infant" (p. 52). "The women saw their situation as never-ending. Loneliness, boredom and a lack of a sense of achievement were key factors in the women's unhappiness with their mothering abilities" (p. 52). | N/A | N/A | "Friends are all at work ... we have only one car ... I feel like my pram is my only release at all to get out ... with two kids I very rarely get to sit on the 'phone and have a chat ... to someone. All I've got to do is housework and that's it ... there's got to be something more to this than just housework" (p. 51). "The loneliness and isolation of motherhood was overwhelming. One woman discussed the comforting presence of her partner on the weekend even though they did not communicate a great deal: You're just stuck at home ... on the weekends my husband - he could go and do things around the house ... I said to him I don't want him to ... sit and do anything with me but just having someone around makes a difference, you just get so lonely (p. 52). | Work outside the home: "I have a job, it's waitressing and cooking at a bed and breakfast and it's a really fun atmosphere and I just love it because even though I'm going to work and it's really hard work and hard on your legs its fun, you've got other grown up conversation ... it's worth it, it's my outing" (p. 52). Participants attended an Early Parenting Center intervention to assist with the sleep problems. The participants stated that being able to talk with other moms going through the same thing was helpful: "It's been really terrific to ... hear that you are not the only one who's having exactly the same problem" (p. 52). "The women found being at an EPC allowed them to gain knowledge, develop confidence, see themselves as "normal" and most importantly feel good about themselves" (p. 53). | N/A |
| Russo, A., Lewis, B., Joyce, A., Crockett, B., & Luchters, S. (2015). A qualitative exploration of the emotional wellbeing and support needs of new mothers from Afghanistan living in Melbourne, Australia. *BMC Pregnancy and Childbirth, 15*, 197. https://doi.org/10.1186/s12884-015-0631-z | N/A | "Participants consistently discussed experiencing emotional challenges following birth, identifying symptoms commonly associated with postnatal depression. Women largely attributed this emotional state to separation from family and culture, leading to loneliness, isolation, and disconnection. Participants expressed resistance towards professional support due to cultural stigma associated with mental illness. Partner support was seen to be positive but difficult to negotiate. Religion, strong relationship with child, forming friendships, education, and utilising childcare were identified as positive influences on the emotional wellbeing of women.” | N/A | N/A | "Separation from family, in particular female kin, was a strong theme within this study, and this was perceived as increasing loneliness and isolation during the postnatal period” (p. 10). | "For some women, religion was considered to be a protective factor against social isolation" (p. 10). | N/A |
| Sable, M. R., Washington, C. C., Schwartz, L. R., & Jorgenson, M. (2007). Social well-being in pregnant women: intended versus unintended pregnancies. *Journal of Psychosocial Nursing and Mental Health Services, 45*(12), 24-31. | N/A | Women whose pregnancies were intended reported higher levels of social support than did those with unintended pregnancies. Women who expressed happiness about having a baby had higher levels of social support and lower levels of loneliness and family relationship problems. Women who thought a baby would fill a void in their lives reported higher loneliness than did women who did not feel this way. Psychosocial interventions with women may help prevent psychosocial correlates of unintended pregnancy. | N/A | 20-item Revised UCLA Loneliness Scale | The extent to which women believed that having a baby would fill an emptiness in their lives was positively correlated with loneliness (r = 0.321, p < 0.01). "Women with unintended pregnancies had significantly lower levels of overall social support, family support, and significant other support compared with those of women with intended pregnancies," (p. 29). "Women who expressed higher levels of agreement that the baby would fill a void in their lives had higher levels of perceived loneliness” (p. 29). | The extent to which women reported being glad to be having a baby was significantly correlated with all three scales; women who were happier about having a baby had lower loneliness scores (r = -0.403, p < 0.01). Happiness was positively correlated with social support (r = 0.341, p < 0.01) and negatively correlated with family relations, (r = -0.307, p < 0.01), indicating that women happier about having a baby reported higher levels of social support and lower levels of family problems" (p. 29). | n/a |
| Samano, R., Martinez-Rojano, H., Robichaux, D., Rodriguez-Ventura, A. L., Sanchez-Jimenez, B., de la Luz Hoyuela, M., Godinez, E., & Segovia, S. (2017). Family context and individual situation of teens before, during and after pregnancy in Mexico City. *BMC Pregnancy and Childbirth, 17*(1), 382. https://doi.org/10.1186/s12884-017-1570-7 | N/A | "The girls disclosed feelings of repression, loneliness and indifference to their parents, leading them to unprotected sexual relations without fear of pregnancy." "Almost all said that they were seeking love outside the family, which revealed a scenario of limited communication and unsatisfactory relations within the family." | N/A | N/A | 21 of the 29 of the mothers stated that they "had sexual relations with the intention of seeking love. In this sense, the teenagers emphasized that their loneliness and the indifference and repression that they perceived from their mother and father led them to secretly go out with a boy, resulting in brief dating periods before sexual relations began" (p. 6). | "Some teen mothers mentioned that their child was an essential element in their life to avoid feeling lonely” (p. 11). | N/A |
| Santos, H. P., Jr., Kossakowski, J. J., Schwartz, T. A., Beeber, L., & Fried, E. I. (2018). Longitudinal network structure of depression symptoms and self-efficacy in low-income mothers. *PloS One, 13*(1), e0191675. https://doi.org/10.1371/journal.pone.0191675 | N/A | The strongest relationships among depression symptoms were lonely - sleep difficulties and inability to get going - crying | N/A | Center for Epidemiological Studies Depression (CES-D) | "Our analysis of the network structure showed that the consistently strongest edges were lonely-sleep disturbance, inability to get going-crying, and concentration difficulty-feeling disliked” (p. 10). | N/A | N/A |
| Saunders, T., & Lawrence, J. (2018). Coming full circle: Building a sustainable community of mothers. *Practising Midwife, 21*(3), 35-38. | N/A | "The unexpected experience of social isolation and loneliness for new mothers is a growing issue in caring for postnatal women in the UK. Inadequate support as a result of fragmented communities and extended family, can leave women at risk of poor mental health with a lasting impact on the woman and her family.” | N/A | N/A | "Families needed to talk to someone who understood, and were awaiting a visit in anticipation” (p. 36). | The author created a mum's circle to support new moms. It was a 2-hour weekly gathering with breastfeeding support and various family support. The primary goal of the group was for the mothers to be able to build self-sustaining relationships. | N/A |
| Schuez-Havupalo, L., Lahti, E., Junttila, N., Toivonen, L., Aromaa, M., Rautava, P., Peltola, V., & Raiha, H. (2018). Parents' depression and loneliness during pregnancy and respiratory infections in the offspring: A prospective birth cohort study. *PloS One, 13*(9), e0203650. https://doi.org/10.1371/journal.pone.0203650 | N/A | "Maternal depressive symptoms during pregnancy predicted higher rates of acute otitis media in the infant and maternal emotional loneliness predicted higher rates of physician visits. Acute otitis media, physician visits and antibiotic consumption in the infant were slightly less frequent for families who reported social loneliness in the father or mother." "Comparing mothers and fathers, mothers had more depressive feelings (Cohen's d 0.948) and fathers more emotional loneliness (Cohen's d 0.282) at gestational week 20" (p. 5). | “Loneliness is subjective anxiety causing feelings of being without the type of relationships the person desires, i.e. a discrepancy between one's real and desired relationships. Social loneliness refers to the absence of a social network, or to the feeling that one is not part of a group. Emotional loneliness, in turn, refers to the lack of a close, intimate attachment to another person [18,20]" (p. 3). | Finnish shortened version of the UCLA Loneliness Scale | "Maternal prenatal depression and emotional loneliness predicted a higher burden of respiratory tract infections in the offspring. The protective influence of parental social loneliness on the burden of respiratory tract infections in infants was not in line with our study hypothesis, but could be explained by reduced use of healthcare services in these socially isolated families." "Feelings of emotional loneliness in the mother predicted more physician visits for the child," (p. 8). "The effect of social loneliness was contradictory to others, i.e. more feelings of social loneliness predicted lesser numbers of AOM (P = 0.003/ mother, P = 0.01/ father), antibiotic consumption (P = 0.04/ mother, P = 0.009/ father), and physician visits (P = 0.001/ mother, P = 0.02/ father),” (p. 8). The authors relate this finding to possible reduced use of healthcare in social isolated families.” | N/A | N/A |
| Shorey, S., Chee, C. Y. I., Ng, E. D., Lau, Y., Dennis, C. L., & Chan, Y. H. (2019). Evaluation of a Technology-Based Peer-Support Intervention Program for Preventing Postnatal Depression (Part 1): Randomized Controlled Trial. *Journal of Medical Internet Research, 21*(8), e12410. https://doi.org/10.2196/12410 | N/A | The technology-based peer-support intervention program was found to be effective in reducing the risk of postnatal depression among new mothers and showed a generally positive trend in reducing postnatal anxiety and loneliness and increasing perceived social support. | N/A | UCLA Loneliness Scale (ULS) 10-item | N/A | “In terms of loneliness, there was an increase in loneliness scores for both groups from baseline to 1 month postpartum, with the control group having a steeper increase than the intervention group. Although loneliness scores continue to increase for mothers in the control group from 1 month to 3 months, loneliness scores for mothers in the intervention group decreased. This is evident that although the PIP was not able to fully relieve the sense of loneliness among mothers during the postpartum period, it still buffered mothers against loneliness compared with those who did not receive the PIP” (p. 10). | N/A |
| Smith, J. E. (2007). *Prenatal maternal stress and coping among vulnerable rural young women* (Publication Number 3296690) [Doctoral dissertation.] University of South Carolina. ProQuest Dissertations & Theses Global. | N/A | Results of this study contribute to the understanding of selected psychosocial variables, vulnerability variables, stress and coping, and health behaviors pf adolescent women during pregnancy. Findings provide bases for innovative health care interventions with potential for improved client health, enhanced nursing education and continued research specific to health care needs of adolescents during pregnancy. | N/A | UCLA Loneliness Scale (version 3) | Pearson correlations demonstrated that depressive symptoms and loneliness were positively associated with prenatal stress and perceived stress (p. 83). | N/A | N/A |
| Sorenson, D. S. (2003). Healing traumatizing provider interactions among women through short-term group therapy. *Archives of Psychiatric Nursing, 17*(6), 259-269. | N/A | Birth Perception; Posttraumatic Childbirth Stress; and Traumatizing Provider Interaction scores among 19 births confirmed perinatal psychological trauma. Descriptive and statistical comparisons for UCLA Loneliness Scale III, Coo- persmith Self-Esteem Scale, Spielberger’s State-Trait Anxiety Index and Beck Depression Inventory II, revealed marked psychological improvement in postintervention measurements. | N/A | UCLA Loneliness Scale (version 3) | They described that as time post birth progressed, people became increasingly intolerant of birth experience discussions, leading to further relational isolation, loneliness and the belief that there was "something wrong with them" (p. 265). | N/A | N/A |
| Spinetta, J. J. (1978). Parental personality factors in child abuse. *Journal of Consulting and Clinical Psychology, 46*(6), 1409-1414. https://doi.org/10.1037//0022-006X.46.6.1409 | N/A | "The empirically derived set of abuse-potential categories proved useful in significantly differentiating between abusing and nonabusing mothers within the same socioeconomic level in three areas: the tendency to becoming upset and angry, feel- ings of isolation and loneliness, and the fear of external threat and control" (p. 1413). | N/A | A child-abuse questionnaire called Michigan Screening Profile of Parenting | N/A | N/A | N/A |
| Stewart, M., Dennis, C. L., Kariwo, M., Kushner, K. E., Letourneau, N., Makumbe, K., Makwarimba, E., & Shizha, E. (2015). Challenges Faced by Refugee New Parents from Africa in Canada. *Journal of Immigrant & Minority Health, 17*(4), 1146-1156. https://doi.org/10.1007/s10903-014-0062-3 | N/A | Female refugee participants reported loneliness before and following the birth of their child. They felt alone due to diminished social networks. Many new mothers did not have any supporters as most family members were in Sudan or Zimbabwe. Support provided by friends in the local community was minimal (see Table 3 for exemplar quotations). These qualitative findings are reinforced by quantitative results” (p. 1149). | N/A | UCLA Loneliness Scale (version 3) | Not having a family present. Not having made any friends. Additional stressors related to relocation. Cultural ignorance/incompatibility issues with Canadian healthcare workers/care workers. | N/A | N/A |
| Stewart, M., Kushner, K. E., Dennis, C., Kariwo, M., Letourneau, N., Makumbe, K., Makwarimba, E., & Shizha, E. (2017). Social support needs of Sudanese and Zimbabwean refugee new parents in Canada. *International Journal of Migration, Health & Social Care, 13*(2), 234-252. https://doi.org/10.1108/IJMHSC-07-2014-0028 | N/A | Separated from their traditional family and cultural supports, refugee new parents reported isolation and loneliness. They lacked support during pregnancy, birth, and postpartum and had limited interactions with people from similar cultural backgrounds. Refugees required support to access services and overcome barriers such as language, complex systems, and limited financial resources. Support preferences included emotional and information support from peers from their cultural community and culturally sensitive service providers. | N/A | UCLA Loneliness Scale (version 3) | N/A | N/A | N/A |
| Stewart, M., Makwarimba, E., Letourneau, N. L., Kushner, K. E., Spitzer, D. L., Dennis, C. L., & Shizha, E. (2015). Impacts of a Support Intervention for Zimbabwean and Sudanese Refugee Parents: "I Am Not Alone". *Canadian Journal of Nursing Research, 47*(4), 113-140. https://doi.org/10.1177/084456211504700407 | N/A | Participants reported feeling less lonely after joining the support group. Some female participants noted that before the intervention they did not make time for meeting their personal support needs, as their time was consumed with household chores and family needs. However, the support group provided an opportunity for them to connect with others outside their homes," (p. 127). "Moreover, the statistically non-significant decrease in loneliness was illuminated by the qualitative data, which indicated that refugees felt less isolated following the intervention," (p. 133). | N/A | 20-item Revised UCLA Loneliness Scale | Feelings of loneliness related to the social losses of relocation, such as family support and culture (p. 128). | One male participant who described feelings of loneliness as a single parent prior to joining the support group said that following the intervention group members became like his ‘uncles’ or ‘brothers,’ providing timely support," (p. 128). Being able to share experiences of racism, discrimination, and exclusion and knowing that others have had similar experiences provided a sense of community and reassurance that they could do better for their children by being aware and talking with their kids. | N/A |
| Tuominen, M., Junttila, N., Ahonen, P., & Rautava, P. (2016). The effect of relational continuity of care in maternity and child health clinics on parenting self-efficacy of mothers and fathers with loneliness and depressive symptoms. *Scandinavian Journal of Psychology, 57*(3), 193-200. https://doi.org/10.1111/sjop.12284 | Emotional and Social | “As the key finding of our study can be raised that a parent's depressive symptoms might affect less on her/his PSE when the family has been taken care of by the same PHN during pregnancy in MHC and after the baby's birth in CHC," (p. 197). "Our study indicates that relational continuity of care provided by the same PHN in the MHC and CHC may be associated with ... higher levels of mothers' emotional loneliness, (p. 197). The authors have several possible explanations for this finding, including issues related to vulnerable parents, low socioeconomic background, less education, rural living etc. (see page 197). The authors' recommendation to address this: "it is important to clarify how universal MHCs and CHCs should be organized thus that parents' psycho-social and parenting problems can be detected as early as possible and individual support and help for families best enabled" (p. 197). | “The subjective feeling of loneliness, which can be experienced not only in aloneness but also in the company of others, is a distressing emotional response to the discrepancy between desired and achieved levels of social relationships" (p. 193). "The commonly accepted definition of social loneliness is that it refers to the absence of a social network or to the feeling that one is not part of a group, whereas emotional loneliness, in turn, refers to the lack of close, intimate attachment to another person" (p. 193). | Finnish shortened version of the UCLA Loneliness Scale | "We also found out that parents' emotional loneliness significantly influenced several PSE factors, regardless whether the family had experienced relational continuity of care in their MHC and CHC or not” (p. 198). | N/A | N/A |
| Van der Gucht, N., & Lewis, K. (2015). Women's experiences of coping with pain during childbirth: a critical review of qualitative research. *Midwifery, 31*(3), 349-358. https://doi.org/10.1016/j.midw.2014.12.005 | N/A | Many women felt the need for effective support throughout childbirth and described the potential implications where this support failed to be provided. Feeling safe through the concept of continuous support was a key element of care to enhance the coping ability and avoid feelings of loneliness and fear." | N/A | N/A | N/A | The women expressed an increased perception of vulnerability and loneliness during childbirth, relieved by the continued presence of the care provider” (p. 352). | N/A |
| Vicary, J. R., & Corneal, D. A. (2001). A comparison of young women's psychosocial status based on age of their first childbirth. *Family and Community Health, 24*(2), 73-84. | N/A | “In the first domain, individual psychological status, only one statistically significant difference emerged: mothers in the mid group were more likely than women in the later group to feel alone," (p. 79). Mid refers to the time-point in the study. "Relationship status (whether the mother was in a romantic relationship or not) and previous family relationships were also not predictors of loneliness" (p. 79). | NA | The revised UCLA Loneliness Scale was adapted for this study to a 9-item tool | “More specifically, these results support the argument that, in their early young adulthood, women who gave birth as teenagers are not different in terms of psychological health than women who delay childbearing. Where differences do exist, such as greater loneliness for those who were adolescent mothers, psychological status prior to pregnancy explains the deficits. This finding is important as it suggests that it is not teen motherhood itself which necessarily has negative psychological consequences for women, but rather individual characteristics, in place before first childbirth, which affect women's later psychological well-being" (p. 81). | N/A | N/A |
| Webber, G., & Wilson, R. (1993). Childbirth in the north. A qualitative study in the Moose Factory zone. *Canadian Family Physician, 39*, 781-788. | N/A | Significant concerns cited were separation from children, loneliness, boredom, and the hospital accommodations." Loneliness was the second highest rated concern from the women, behind separation for their children (50% of them voiced this concern). | N/A | N/A | “The unfamiliarity of the location con- tributes to their unhappiness- the people, food, accommodation, and activities all differ from their experience at home. One young woman found it particularly difficult to be the only border on the ward, especially because she was a single mother, pregnant for the first time, and miles from her family and partner. 'I was homesick. One time when I was down there, I was on ward 5 [the ward where prenatal patients are boarded]; I ended up being there by myself onward 5. There was hardly anybody else there. Everybody else came home. So I didn't like it staying there. I wanted one of my family members to come with me.... They phoned me every day....They called everyday to find out how I was feeling, and my mother used to ask me,"Do you know how you would feel to be going into labour?" And I said,"No ,I don't," and she used to tell me what to watch out for'" (p. 784). | N/A | N/A |
| Yang, Y. O., Peden-McAlpine, C., & Chen, C. H. (2007). A qualitative study of the experiences of Taiwanese women having their first baby after the age of 35 years. *Midwifery, 23*(4), 343-349. | N/A | “Five subcategories were expressed among pregnant women over 35 years of age. These were 'surprise and worry about childbirth outcomes;' 'embarrassment about being outside the societal age norm for pregnancy;' 'ambivalence about impending lifestyle changes;' 'loneliness and lack of support;' and 'concern about the safety of pregnancy and childbirth'." | N/A | N/A | “The pregnant women felt extremely lonely, as they did not have peers to share their experience" (p. 348). Many women aged 35 or older have children in their teens. These pregnant women aged 35 or greater found it difficult to connect with their peers who were concerned with different development stages of their children. See page 347. "All of my classmates and friends have grown kids already, and they have forgotten their pregnancy experiences. I can't share my feelings with my coworkers who are that much younger! There were many tests that had to be done, for example, amniocentesis had to be done by the 16th week and a diabetes test by the 20th week. I didn't know if amniocentesis would be dangerous or not. I can't relate to my friends since their last pregnancy was years ago. (Mei-Lang)" (p. 347). | N/A | N/A |
| Zaidi, F., Nigam, A., Anjum, R., & Agarwalla, R. (2017). Postpartum Depression in Women: A Risk Factor Analysis. *Journal of Clinical and Diagnostic Research JCDR, 11*(8), QC13-QC16. https://doi.org/10.7860/JCDR/2017/25480.10479 | N/A | “In the present study, it was also observed that the percentage of postnatal women with loneliness was significantly higher in the depressed group of postnatal women than the non-depressed ones. (26.3% vs 6.9%, Chi-square=7.323, p-value= 0.007)” (p. 14). “Previous stressful life events and loneliness were found to be associated more with PPD” (p. 14). | N/A | 6-item De Jong Gierveld Loneliness Scale | "The feeling of loneliness itself implicates that these women are in desire of more social support during this time period” (p. 15). | N/A | N/A |
